# Supplementary figures and images for: Post-phagocytosis activation of NLRP3 inflammasome by two novel T6SS effectors
Source: eLife. 2022 Sep 26;11:e82766. doi: 10.7554/eLife.82766 (PMC9545535; doi:10.7554/eLife.82766)

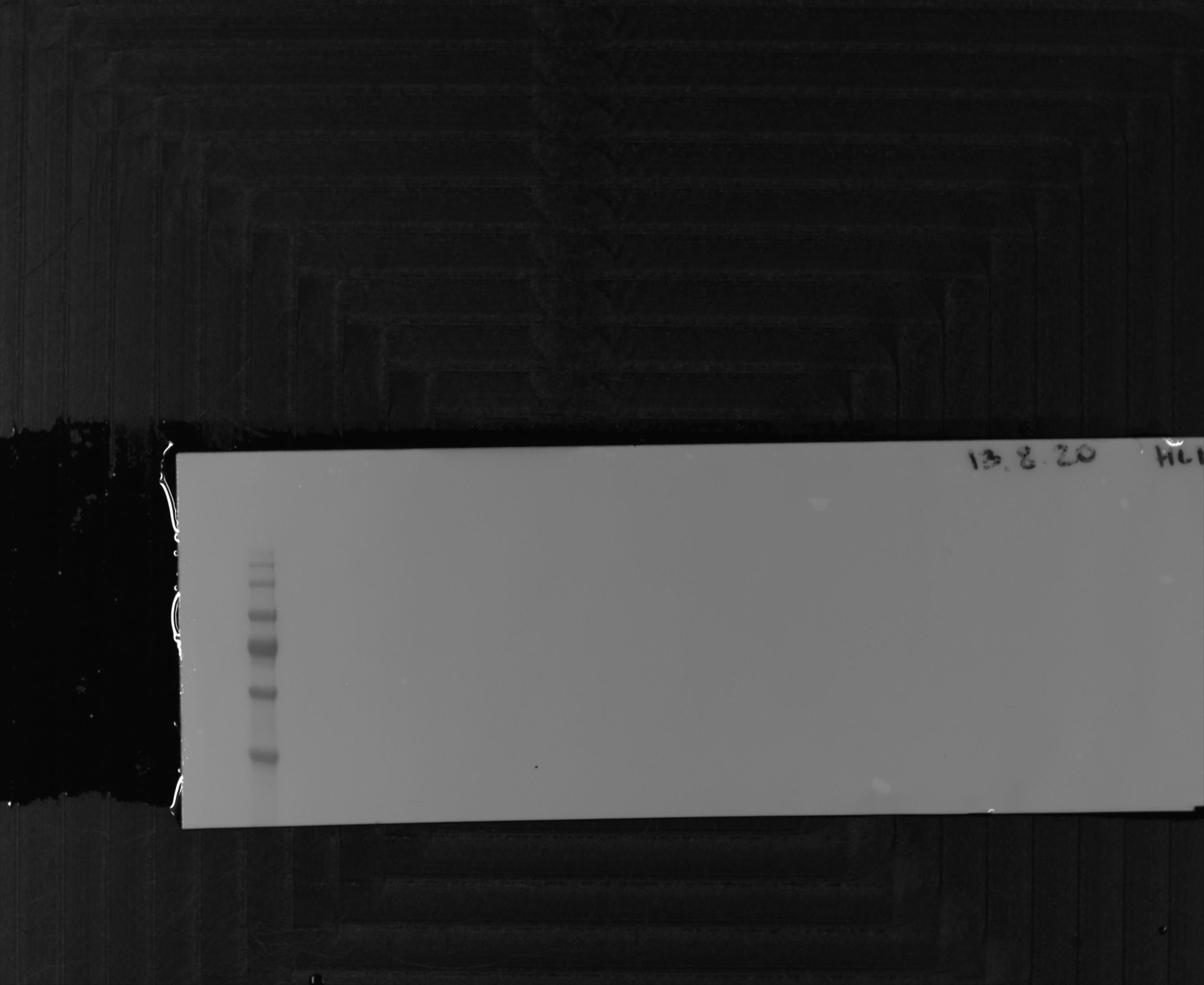

Supplement: Figure 1—source data 1. [file elife-82766-fig1-data1.zip › Figure 1a-source data 1/a-VgrG1/pellet/HC131 pellet 13082020 westar nova VgrG1 ladder.Tif]

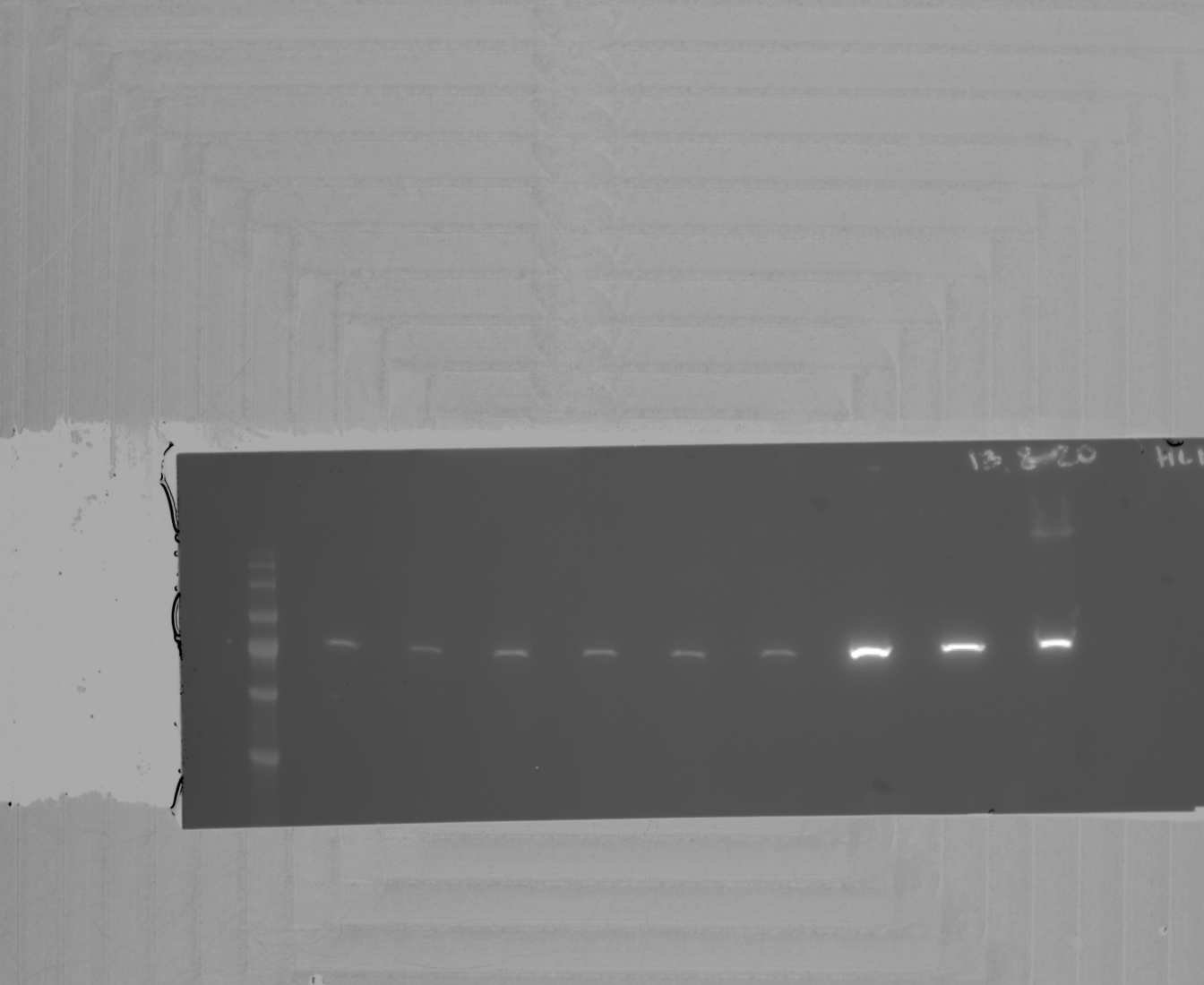

Supplement: Figure 1—source data 1. [file elife-82766-fig1-data1.zip › Figure 1a-source data 1/a-VgrG1/pellet/HC131 pellet 13082020 westar nova VgrG1 merge.Tif]

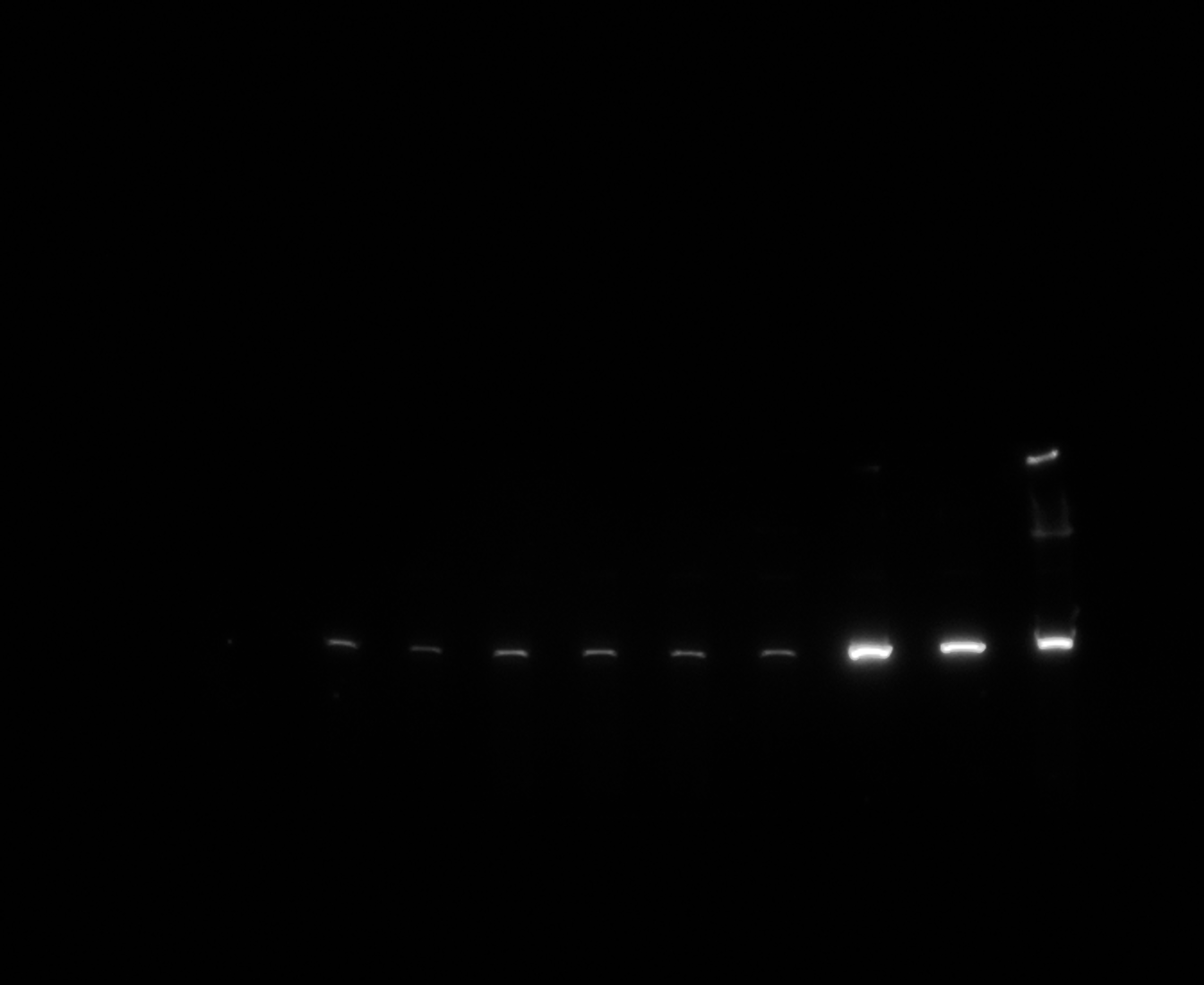

Supplement: Figure 1—source data 1. [file elife-82766-fig1-data1.zip › Figure 1a-source data 1/a-VgrG1/pellet/HC131 pellet 13082020 westar nova VgrG1.Tif]

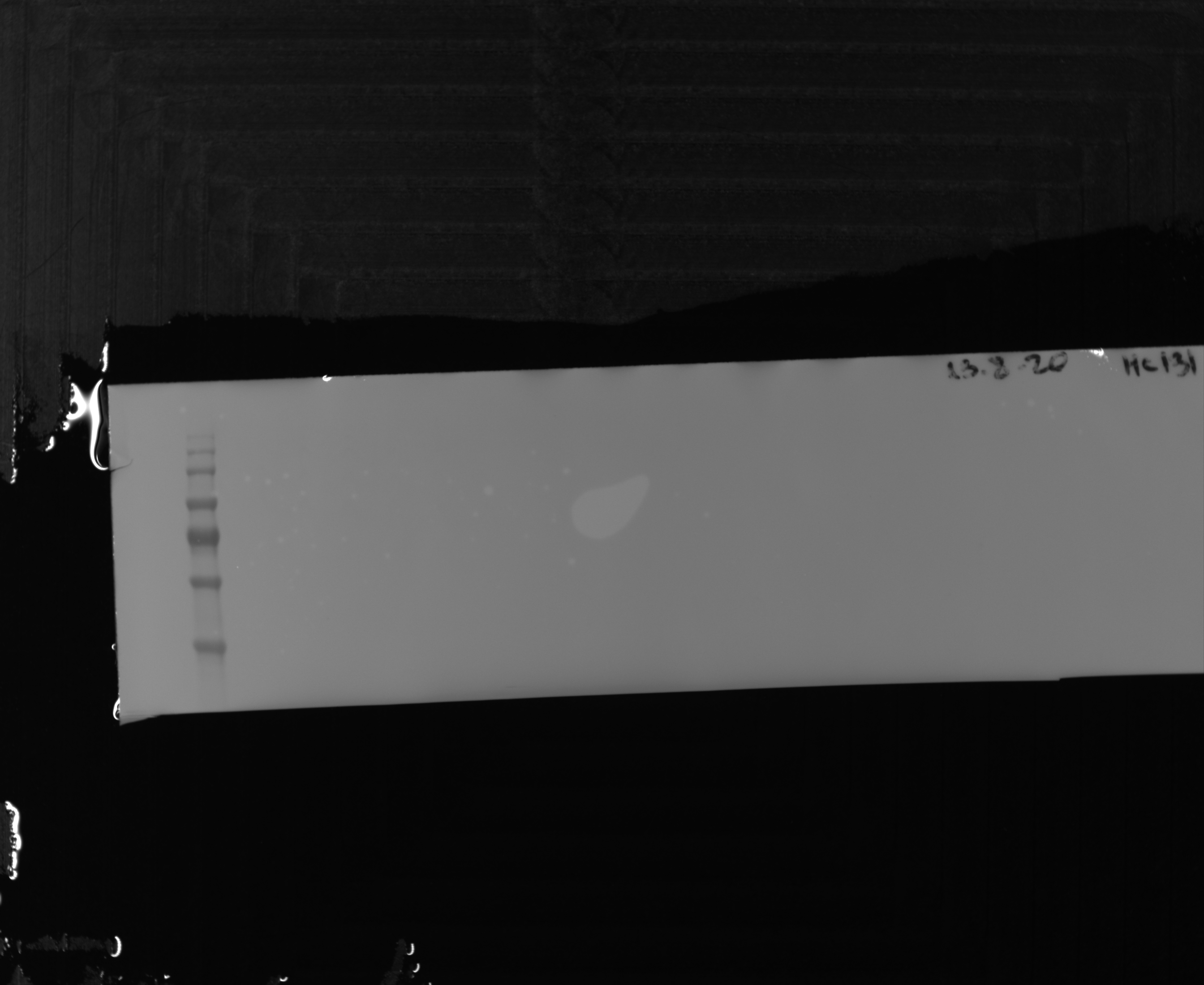

Supplement: Figure 1—source data 1. [file elife-82766-fig1-data1.zip › Figure 1a-source data 1/a-VgrG1/supernatant/HC131 sup 13082020 westar nova VgrG1 ladder.Tif]

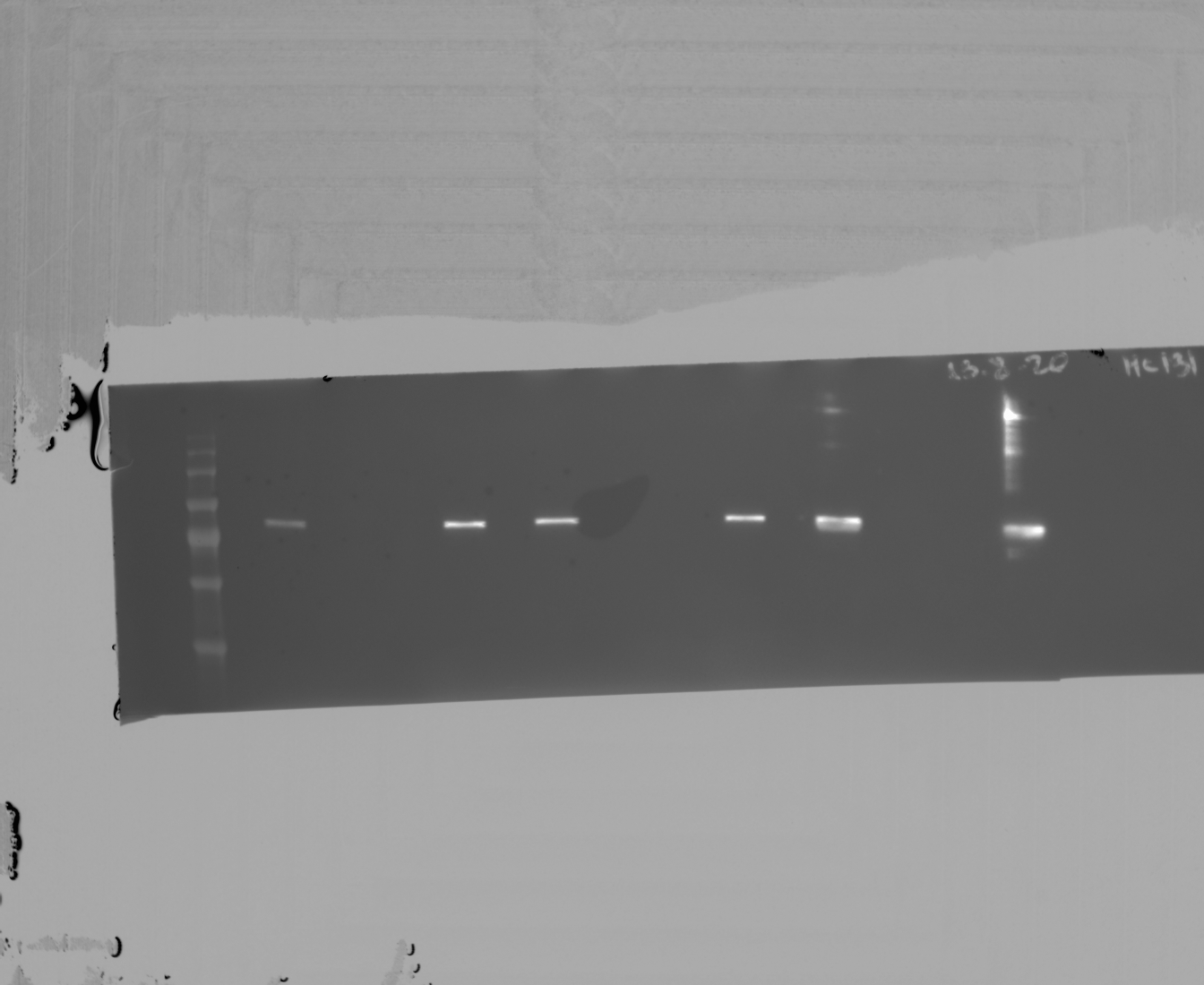

Supplement: Figure 1—source data 1. [file elife-82766-fig1-data1.zip › Figure 1a-source data 1/a-VgrG1/supernatant/HC131 sup 13082020 westar nova VgrG1 merge.Tif]

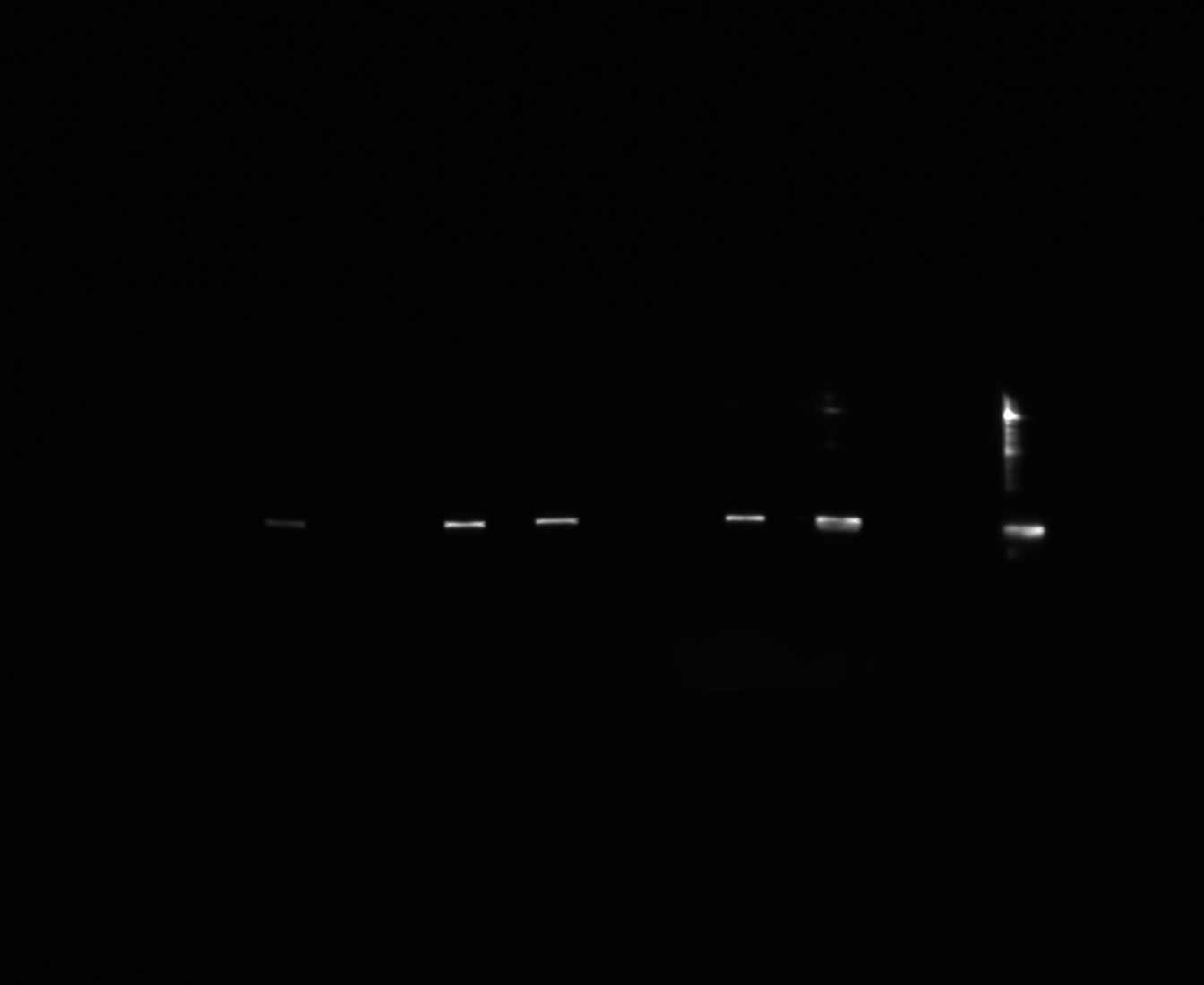

Supplement: Figure 1—source data 1. [file elife-82766-fig1-data1.zip › Figure 1a-source data 1/a-VgrG1/supernatant/HC131 sup 13082020 westar nova VgrG1.Tif]

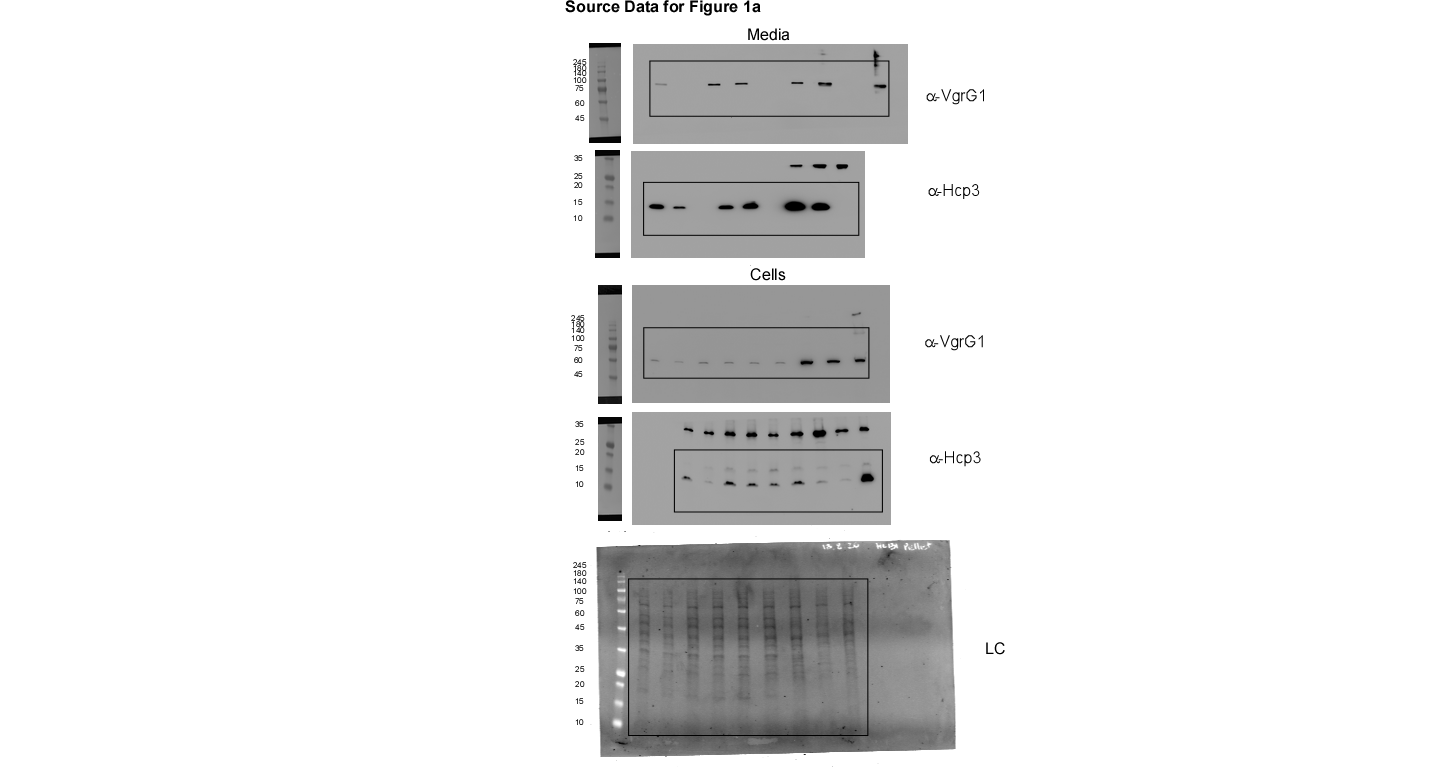

Supplement: Figure 1—source data 1. [file elife-82766-fig1-data1.zip › Figure 1a-source data 1/Figure 1a.tif]

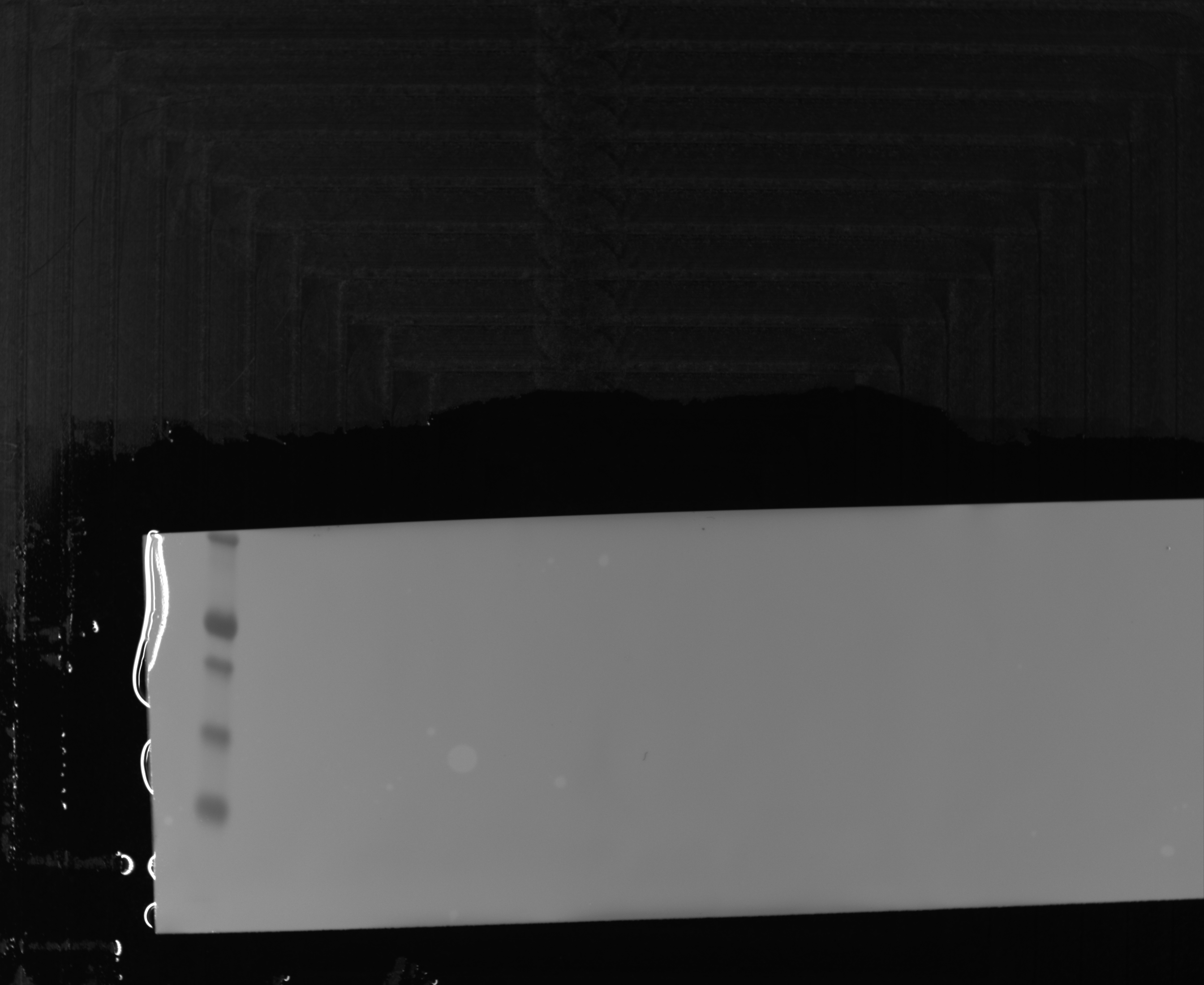

Supplement: Figure 1—source data 1. [file elife-82766-fig1-data1.zip › Figure 1a-source data 1/Hcp3/pellet/HC131 pellet 13082020 westar nova hcp3 ladder.Tif]

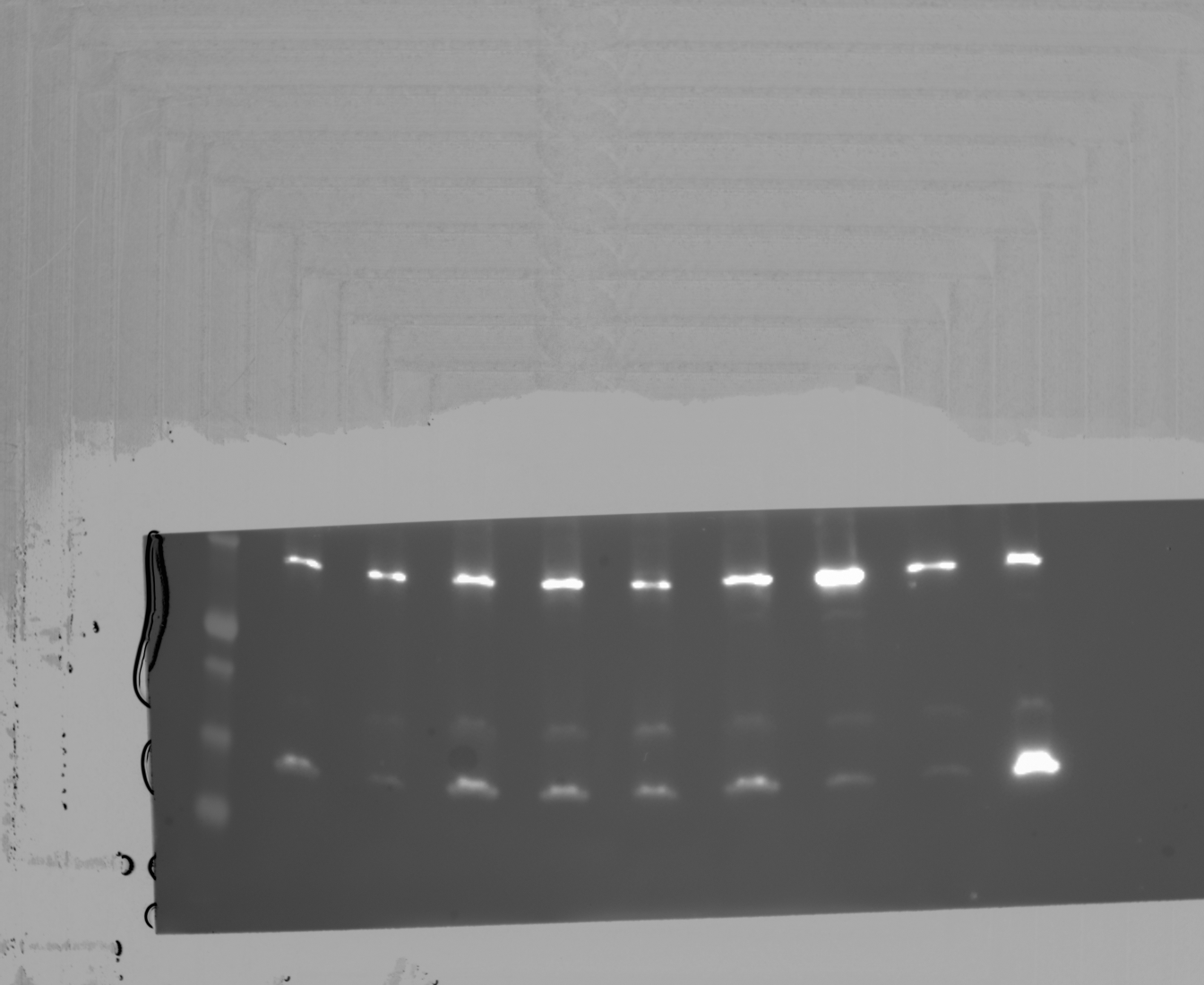

Supplement: Figure 1—source data 1. [file elife-82766-fig1-data1.zip › Figure 1a-source data 1/Hcp3/pellet/HC131 pellet 13082020 westar nova hcp3 merge.Tif]

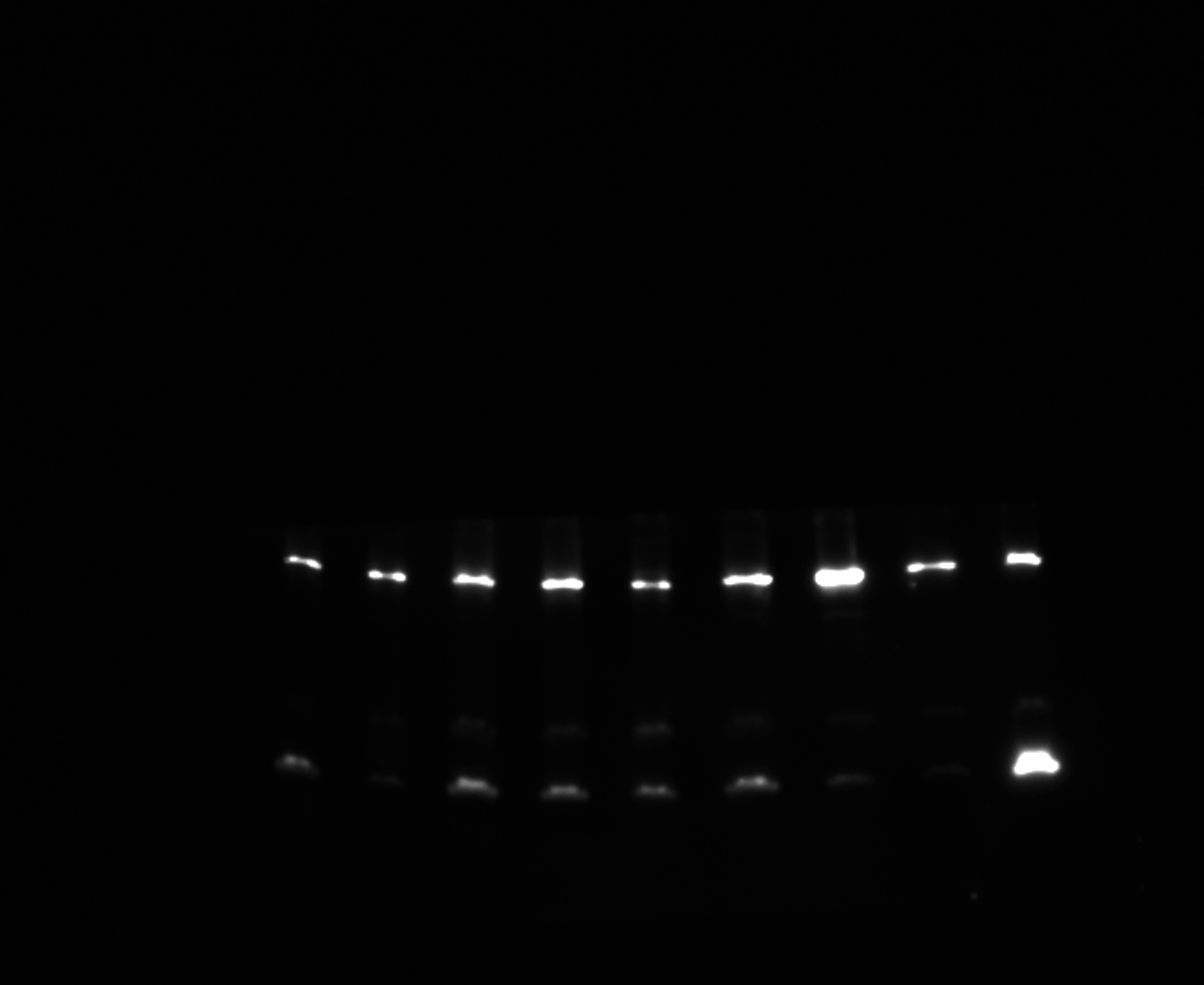

Supplement: Figure 1—source data 1. [file elife-82766-fig1-data1.zip › Figure 1a-source data 1/Hcp3/pellet/HC131 pellet 13082020 westar nova hcp3.Tif]

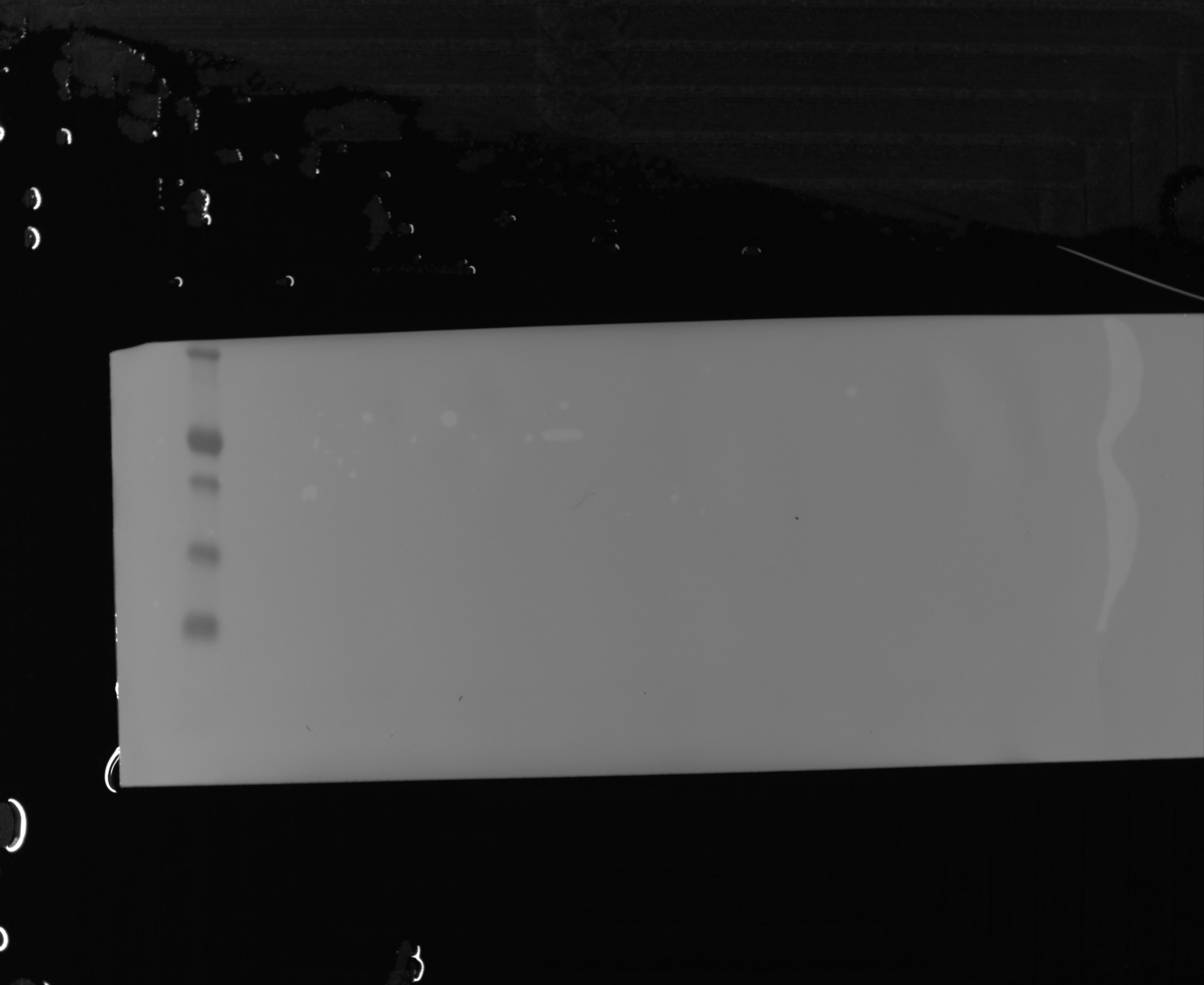

Supplement: Figure 1—source data 1. [file elife-82766-fig1-data1.zip › Figure 1a-source data 1/Hcp3/supernatant/HC131 sup 13082020 westar nova hcp3 ladder.Tif]

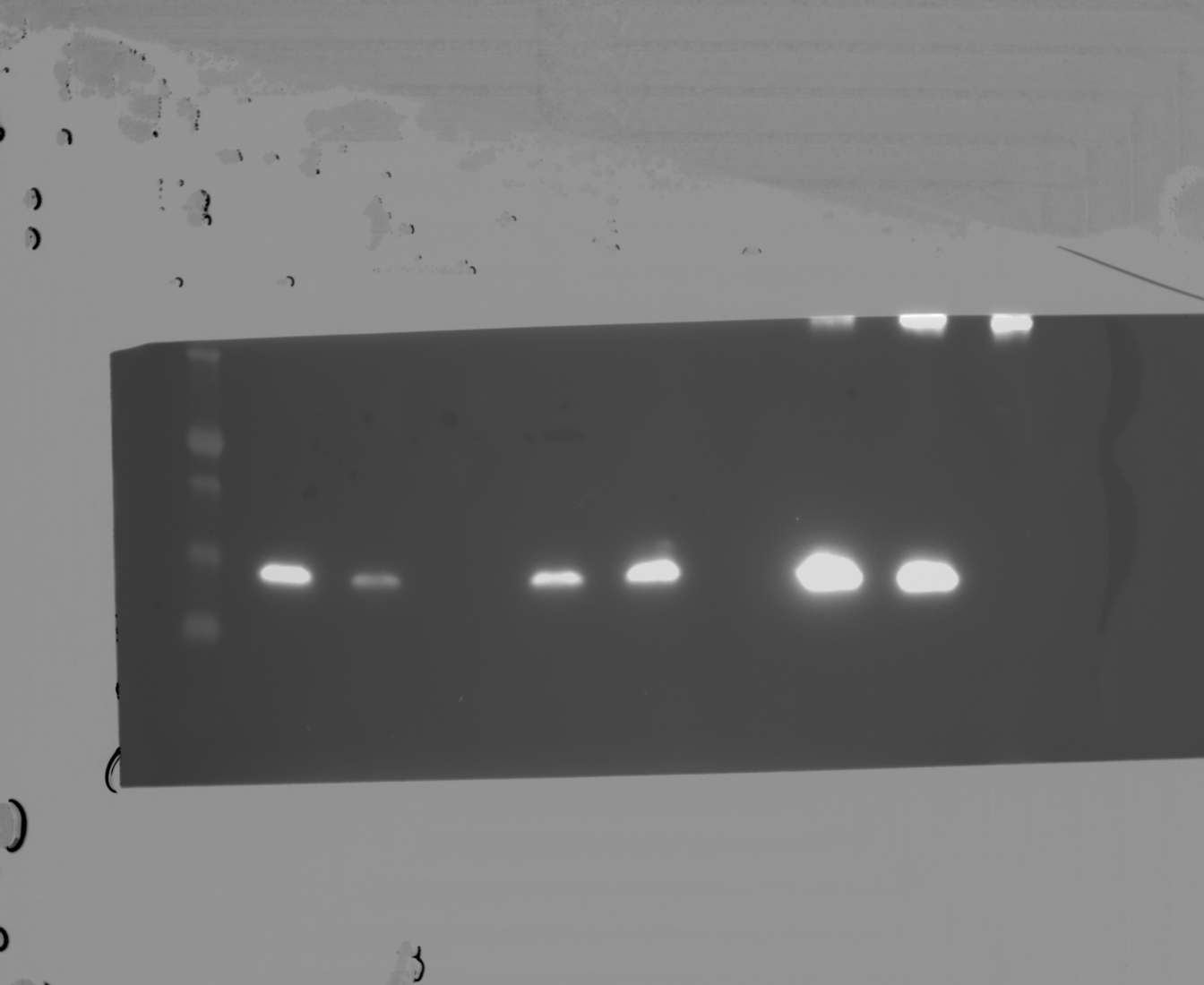

Supplement: Figure 1—source data 1. [file elife-82766-fig1-data1.zip › Figure 1a-source data 1/Hcp3/supernatant/HC131 sup 13082020 westar nova hcp3 merge.Tif]

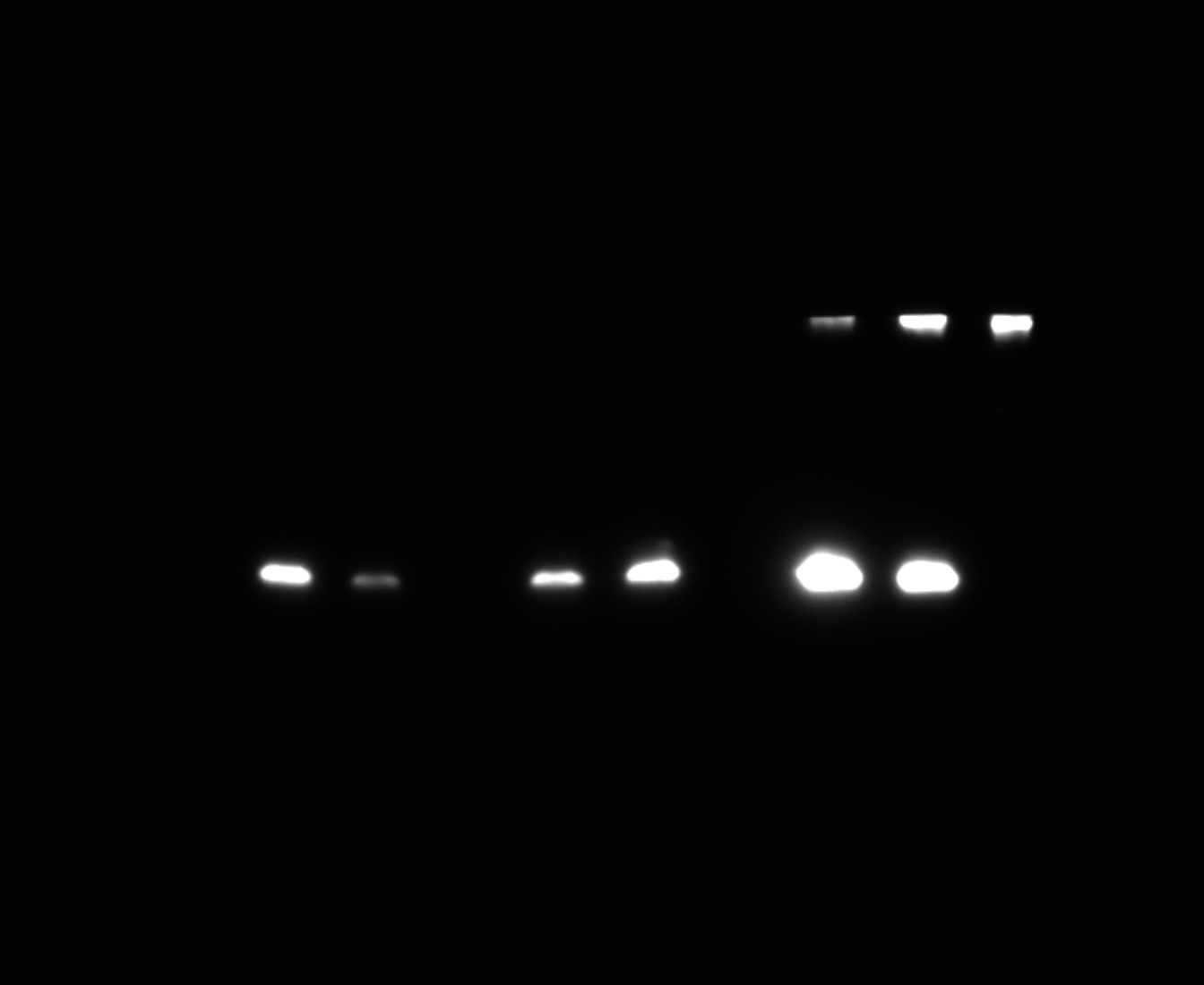

Supplement: Figure 1—source data 1. [file elife-82766-fig1-data1.zip › Figure 1a-source data 1/Hcp3/supernatant/HC131 sup 13082020 westar nova hcp3.Tif]

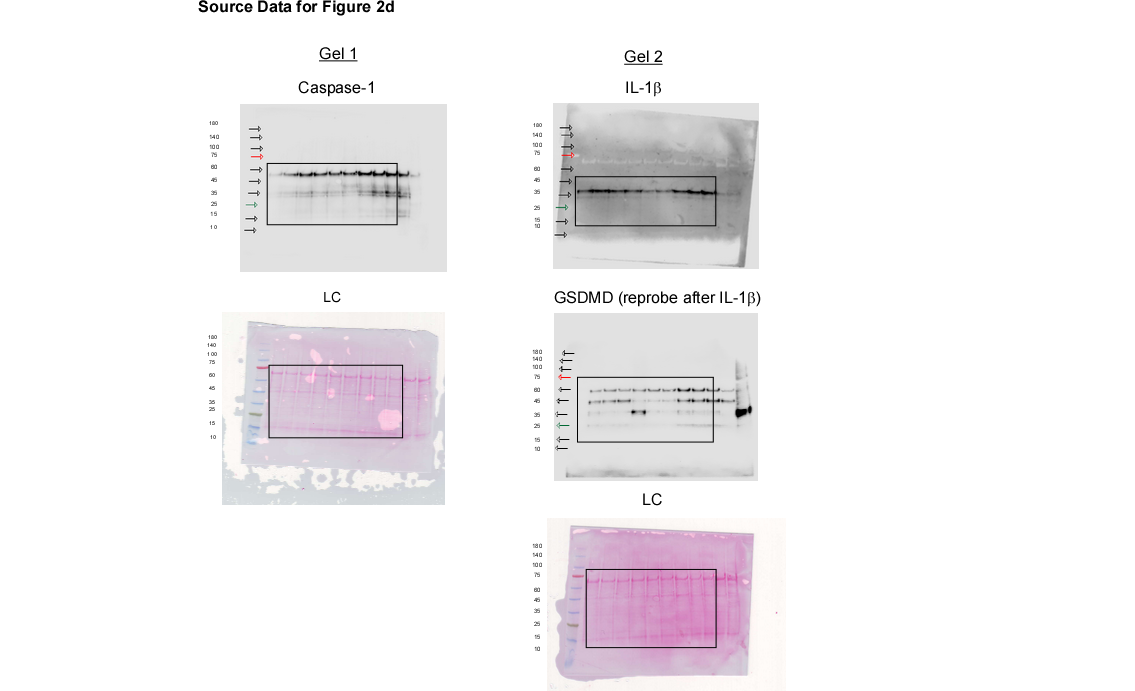

Supplement: Figure 2—source data 1. [file elife-82766-fig2-data1.zip › Figure 2d-source data 1/Figure 2d.tif]

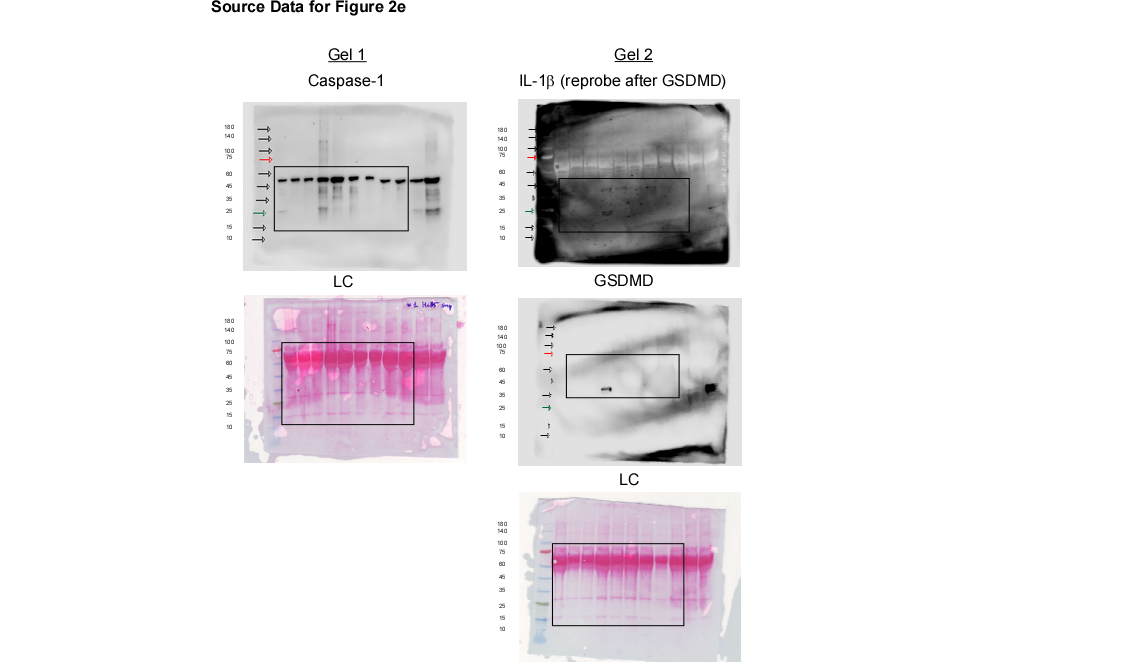

Supplement: Figure 2—source data 2. [file elife-82766-fig2-data2.zip › Figure 2e-source data 1/Figure 2e.tif]

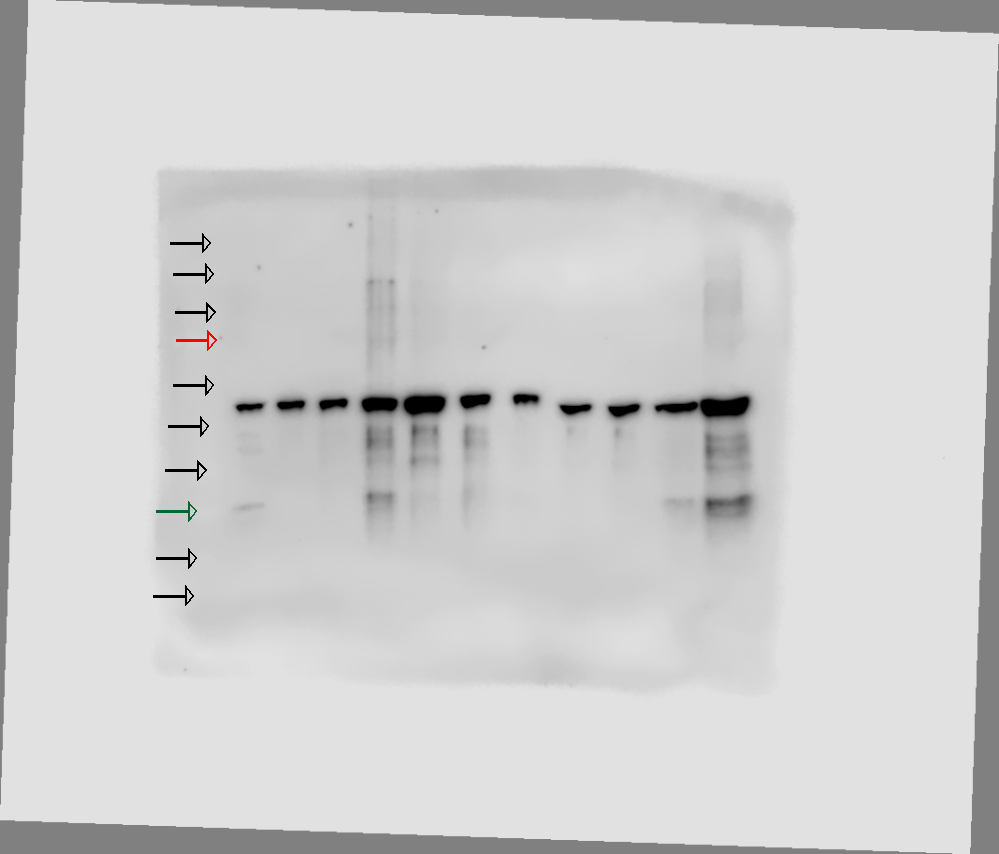

Supplement: Figure 2—source data 2. [file elife-82766-fig2-data2.zip › Figure 2e-source data 1/supernatant caspase1.png]

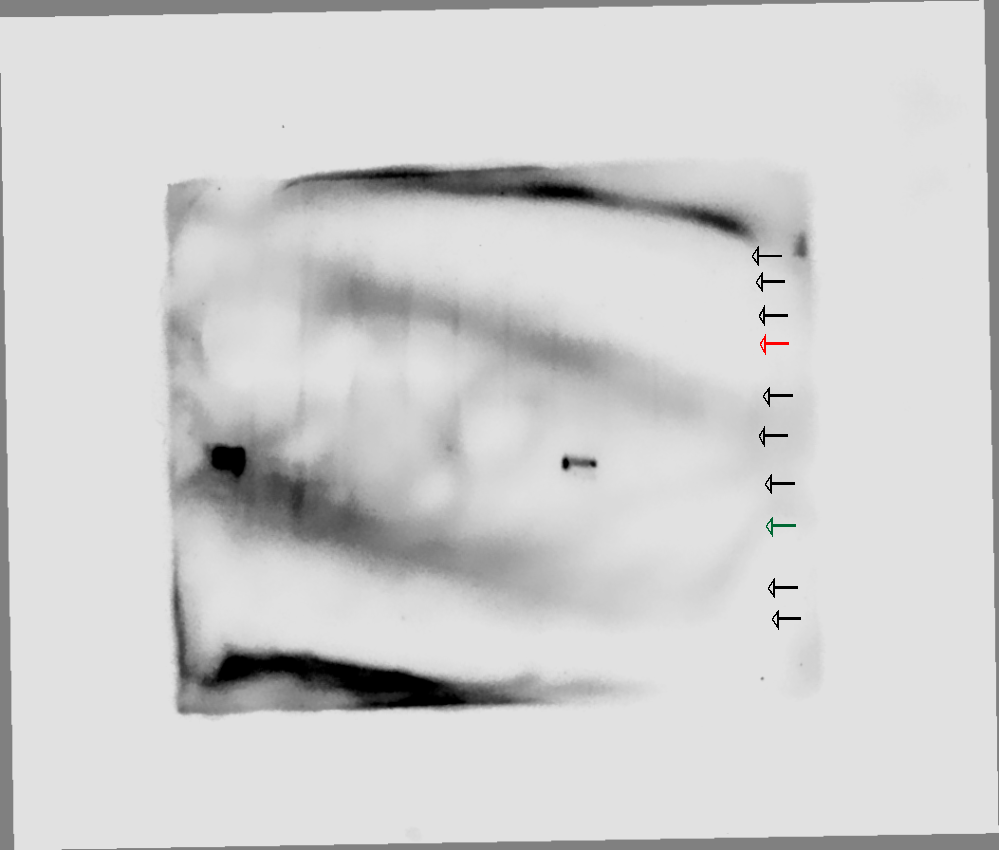

Supplement: Figure 2—source data 2. [file elife-82766-fig2-data2.zip › Figure 2e-source data 1/supernatant GSDMD.png]

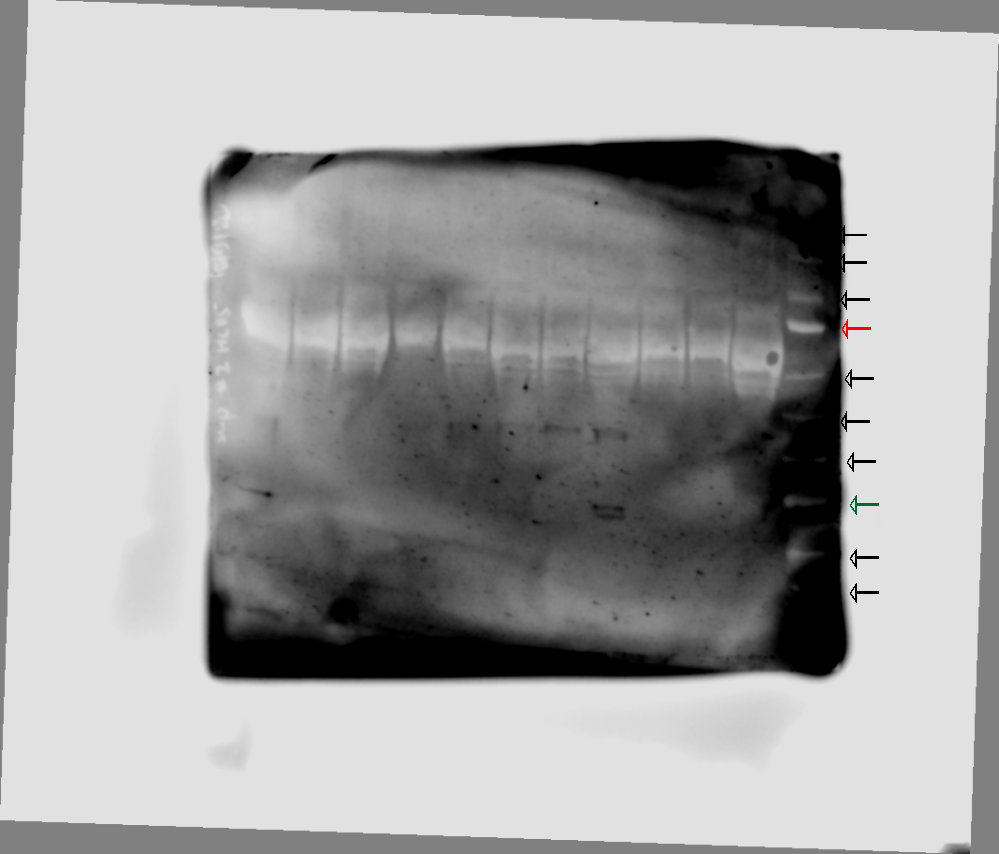

Supplement: Figure 2—source data 2. [file elife-82766-fig2-data2.zip › Figure 2e-source data 1/supernatant IL1b.png]

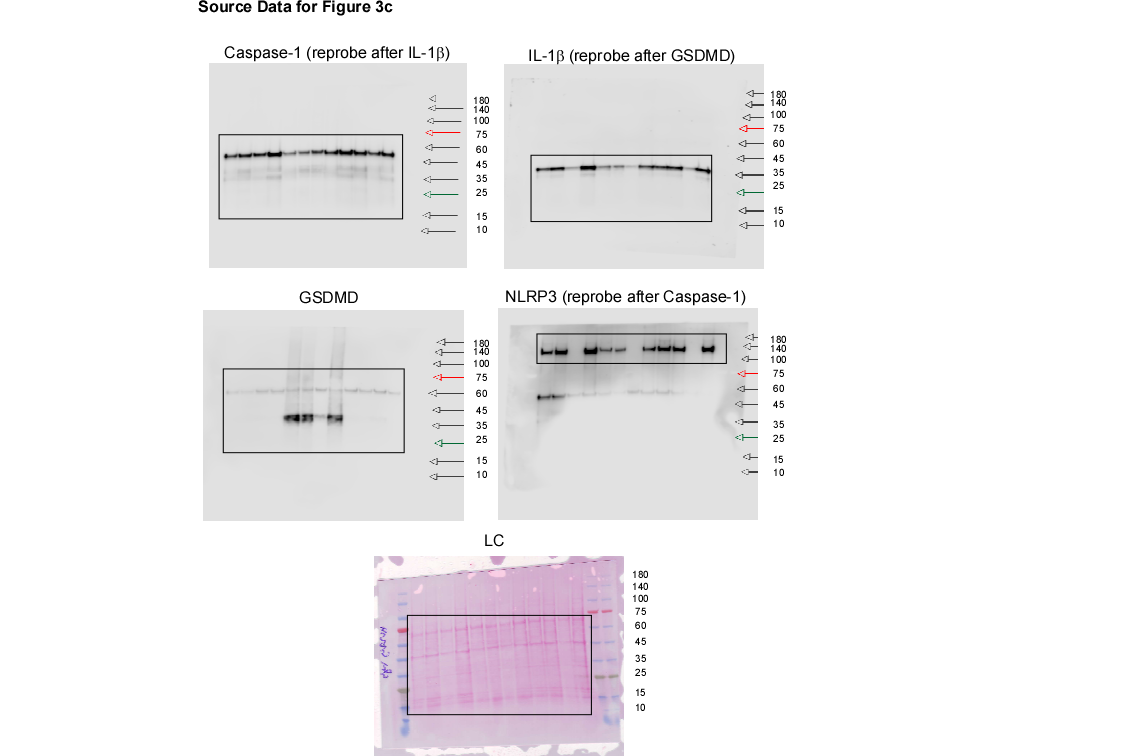

Supplement: Figure 3—source data 1. [file elife-82766-fig3-data1.zip › Figure 3c-source data 1/Figure 3c.tif]

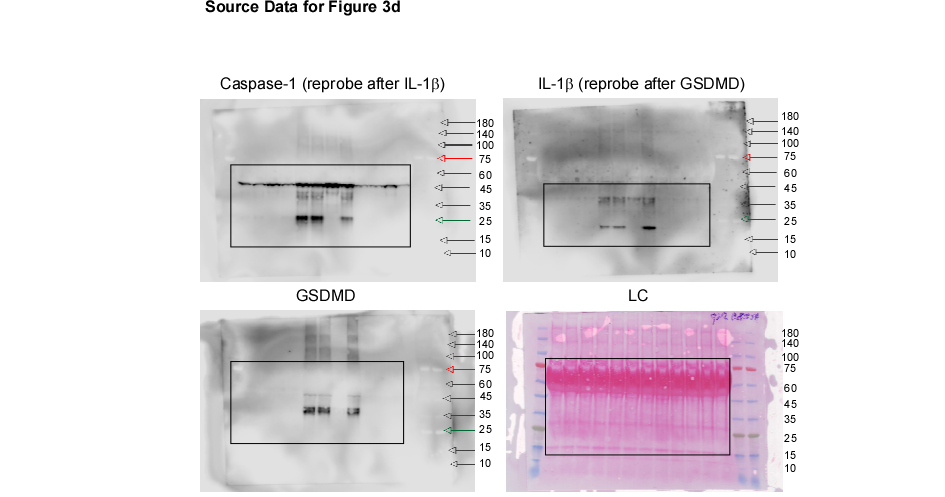

Supplement: Figure 3—source data 2. [file elife-82766-fig3-data2.zip › Figure 3d-source data 1/Figure 3d.tif]

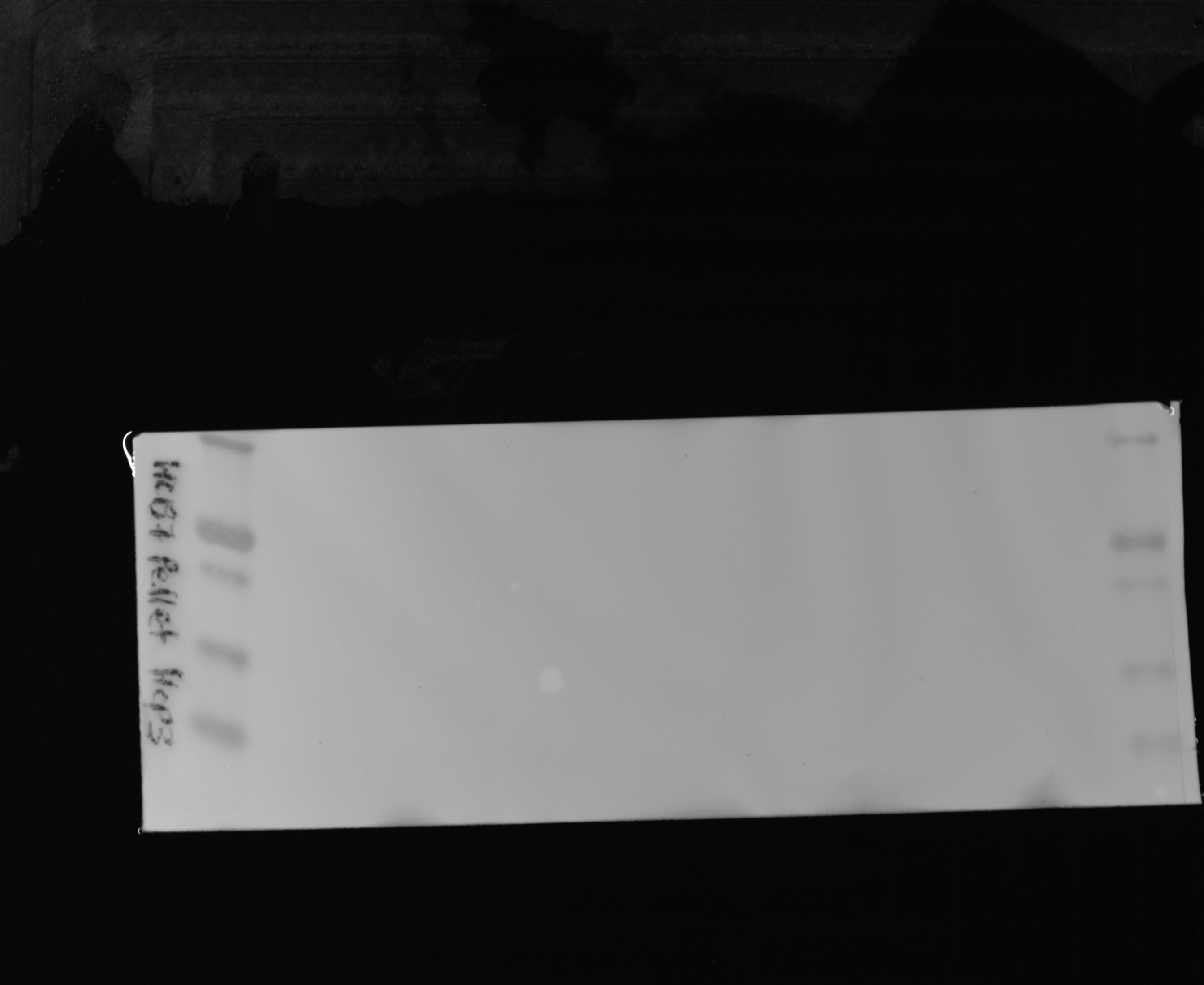

Supplement: Figure 3—figure supplement 1—source data 1. [file elife-82766-fig3-figsupp1-data1.zip › Figure 3-figure supplement 1b-source data 1/a-Hcp3/pellet/06012021 a-Hcp3 sup CM sec pellet ladder.Tif]

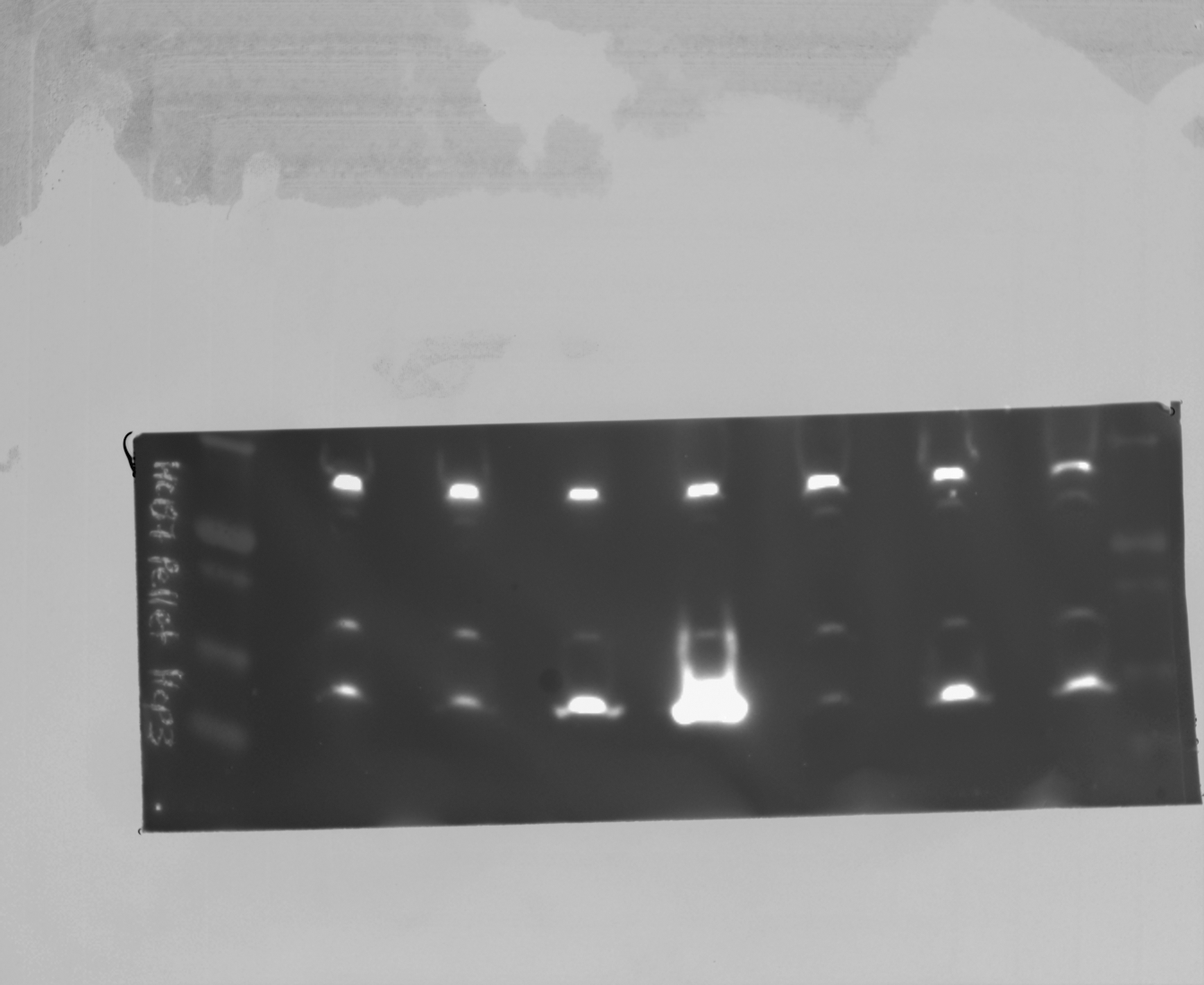

Supplement: Figure 3—figure supplement 1—source data 1. [file elife-82766-fig3-figsupp1-data1.zip › Figure 3-figure supplement 1b-source data 1/a-Hcp3/pellet/06012021 a-Hcp3 sup CM sec pellet merge.Tif]

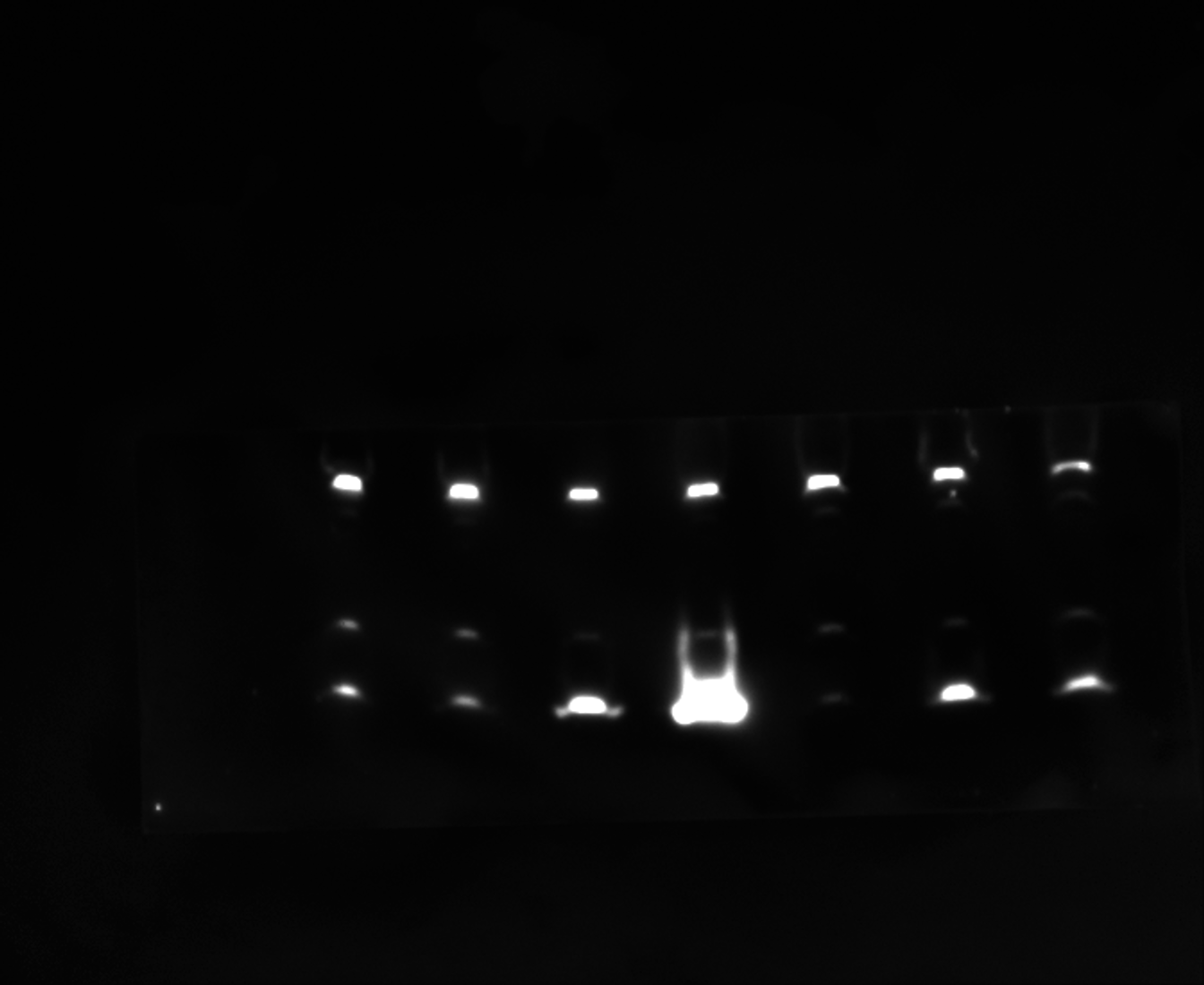

Supplement: Figure 3—figure supplement 1—source data 1. [file elife-82766-fig3-figsupp1-data1.zip › Figure 3-figure supplement 1b-source data 1/a-Hcp3/pellet/06012021 a-Hcp3 sup CM sec pellet.Tif]

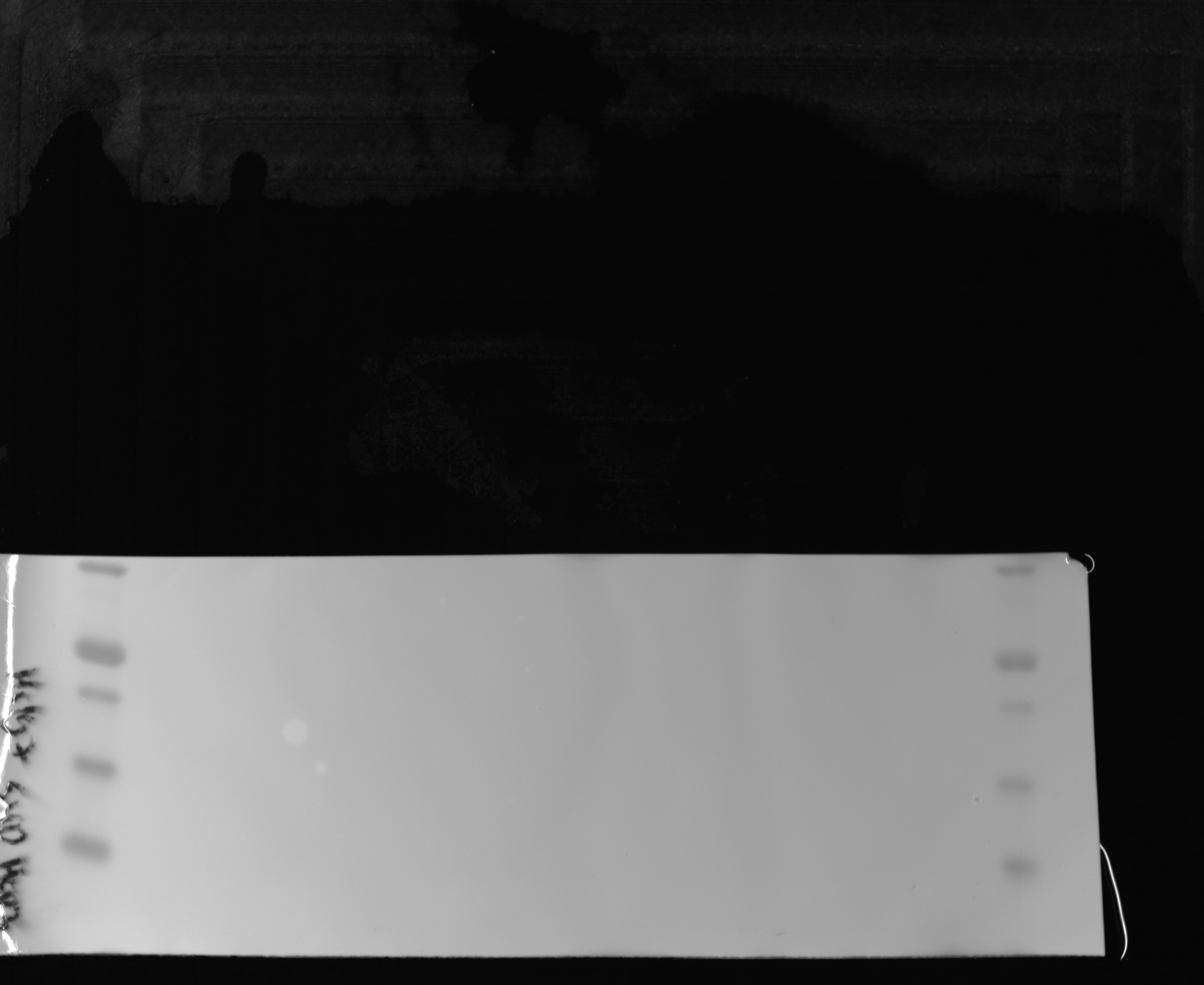

Supplement: Figure 3—figure supplement 1—source data 1. [file elife-82766-fig3-figsupp1-data1.zip › Figure 3-figure supplement 1b-source data 1/a-Hcp3/supernatant/06012021 a-Hcp3 sup WN 2 sec sup ladder.Tif]

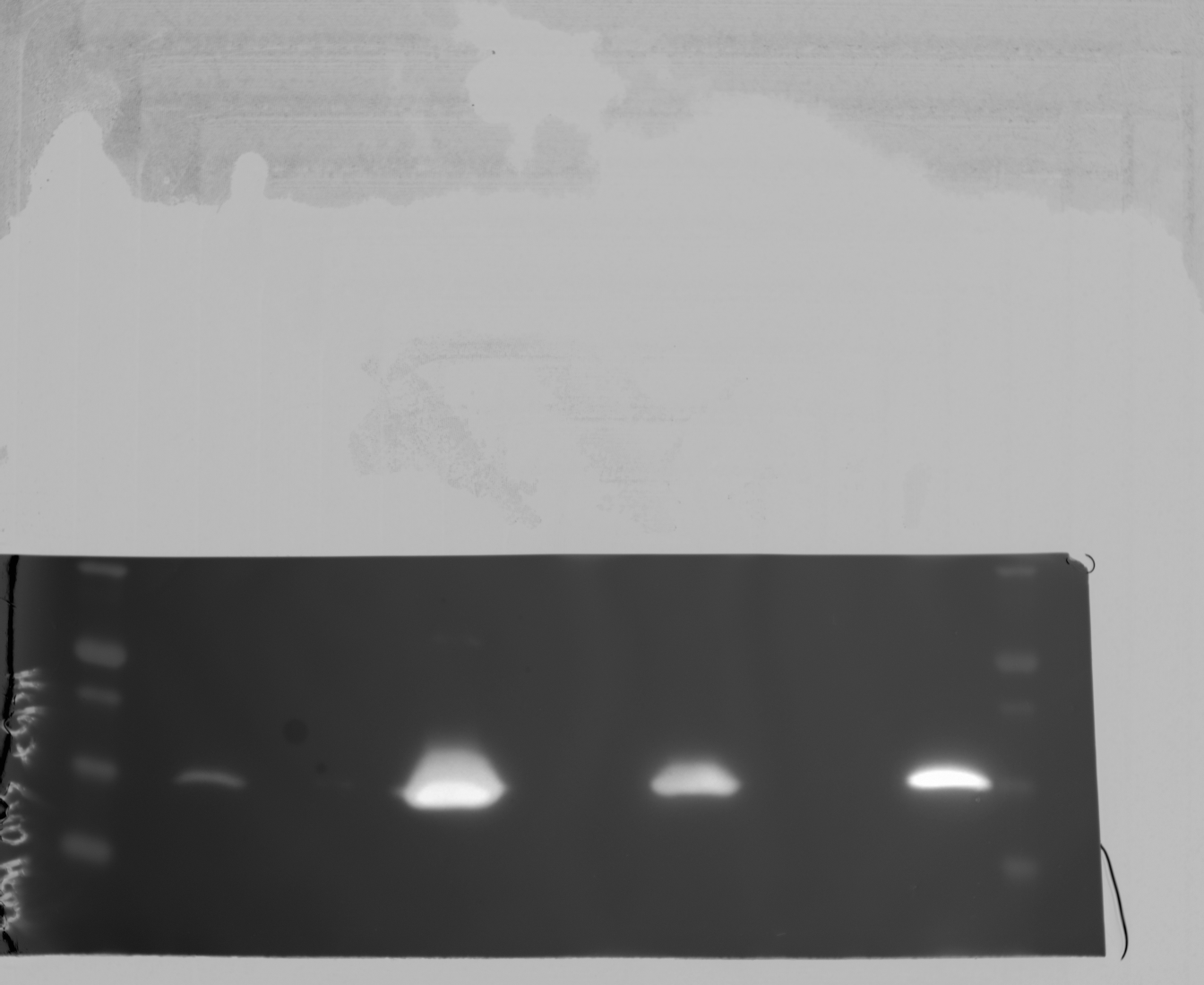

Supplement: Figure 3—figure supplement 1—source data 1. [file elife-82766-fig3-figsupp1-data1.zip › Figure 3-figure supplement 1b-source data 1/a-Hcp3/supernatant/06012021 a-Hcp3 sup WN 2 sec sup merge.Tif]

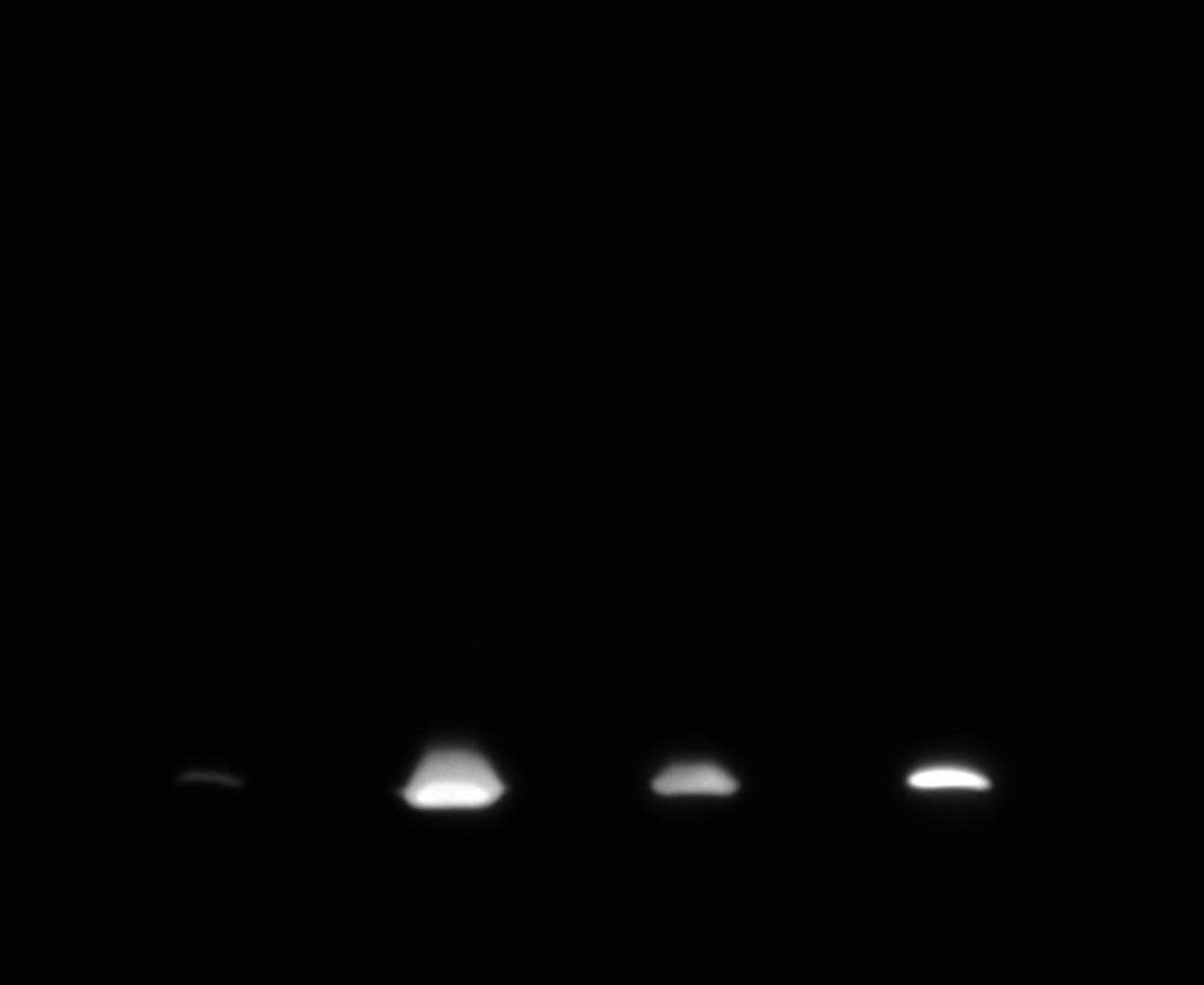

Supplement: Figure 3—figure supplement 1—source data 1. [file elife-82766-fig3-figsupp1-data1.zip › Figure 3-figure supplement 1b-source data 1/a-Hcp3/supernatant/06012021 a-Hcp3 sup WN 2 sec sup.Tif]

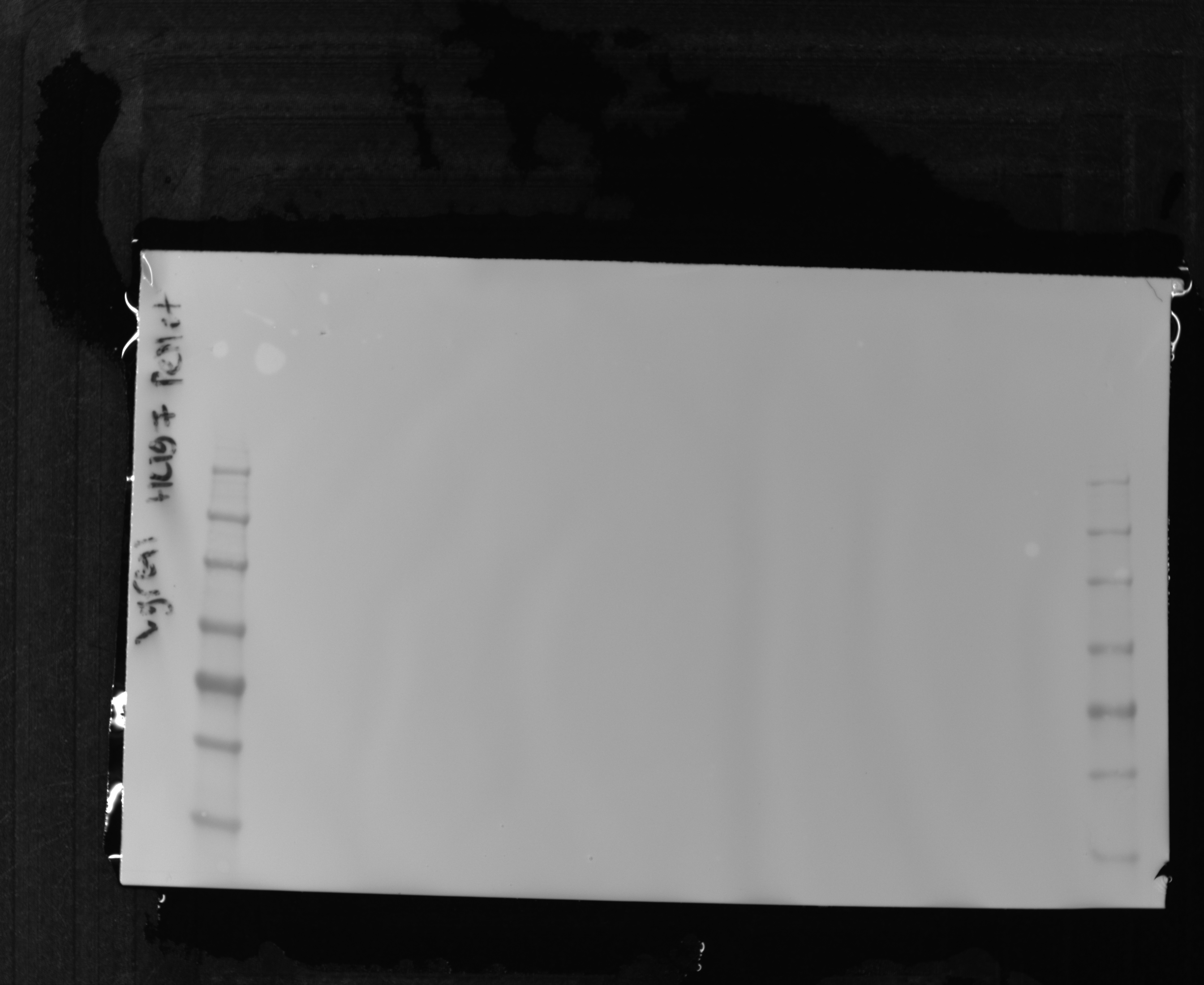

Supplement: Figure 3—figure supplement 1—source data 1. [file elife-82766-fig3-figsupp1-data1.zip › Figure 3-figure supplement 1b-source data 1/a-VgrG1/pellet/06012021 a-VgrG1 sup WN 2 sec pellet ladder.Tif]

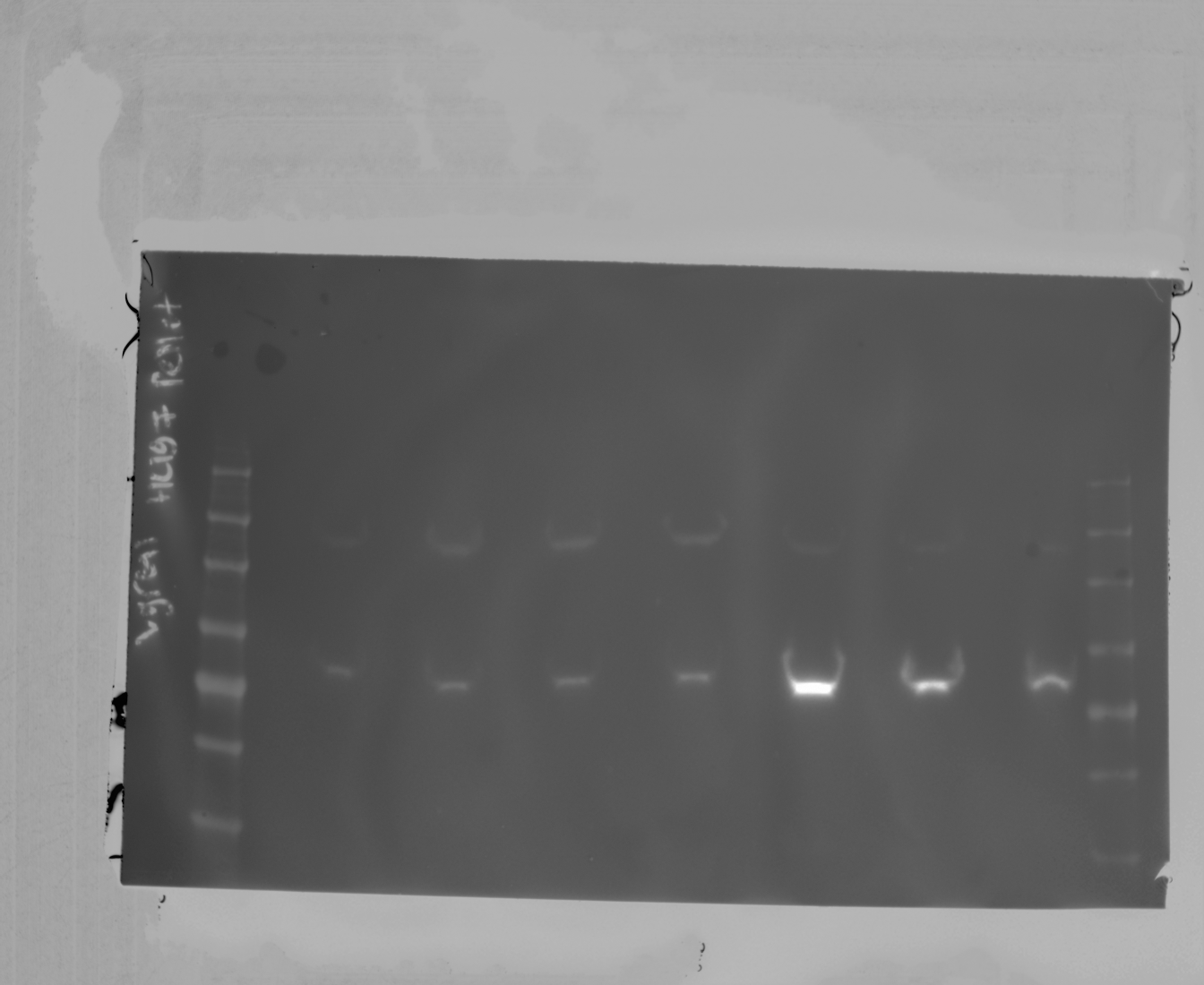

Supplement: Figure 3—figure supplement 1—source data 1. [file elife-82766-fig3-figsupp1-data1.zip › Figure 3-figure supplement 1b-source data 1/a-VgrG1/pellet/06012021 a-VgrG1 sup WN 2 sec pellet merge.Tif]

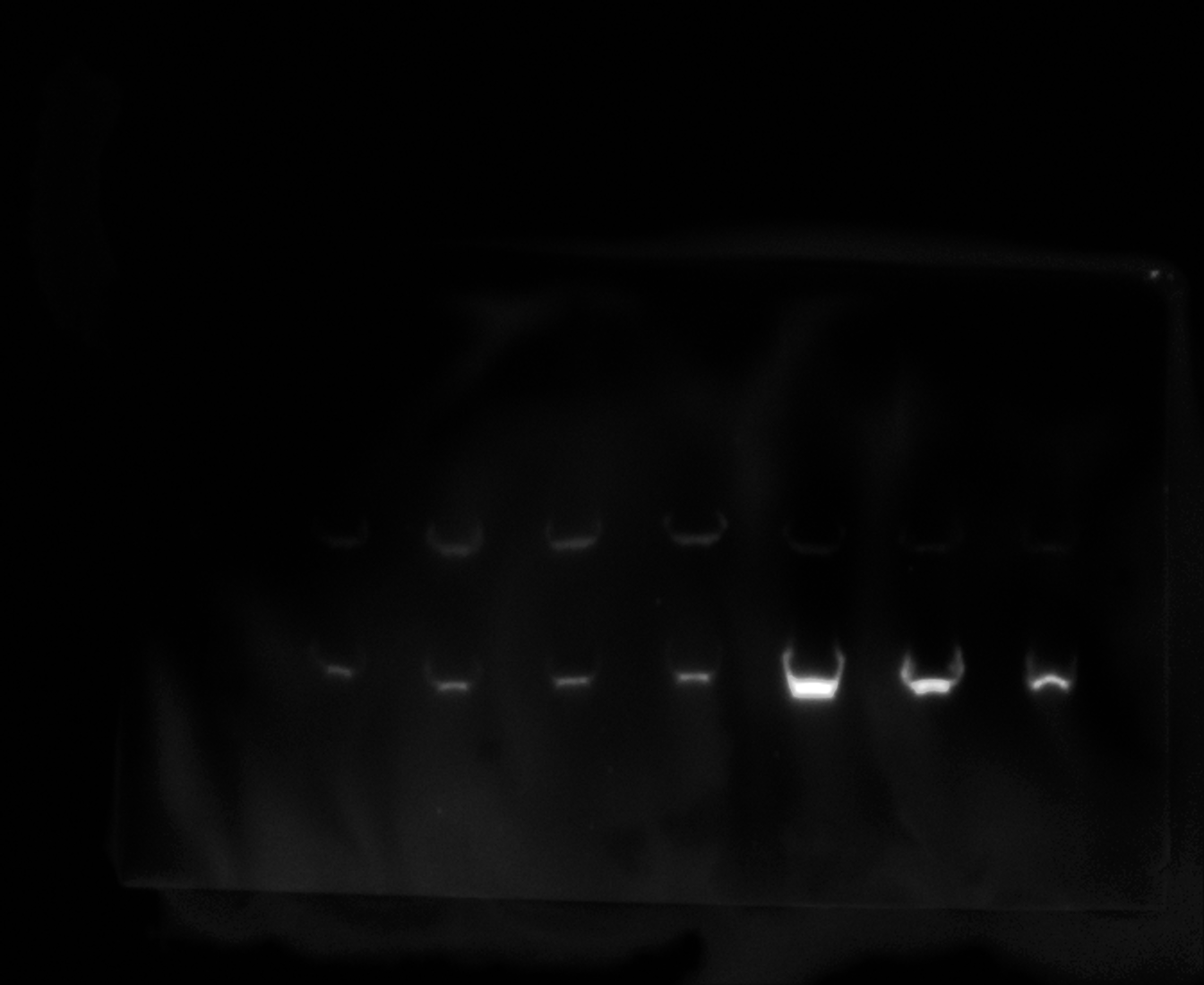

Supplement: Figure 3—figure supplement 1—source data 1. [file elife-82766-fig3-figsupp1-data1.zip › Figure 3-figure supplement 1b-source data 1/a-VgrG1/pellet/06012021 a-VgrG1 sup WN 2 sec pellet.Tif]

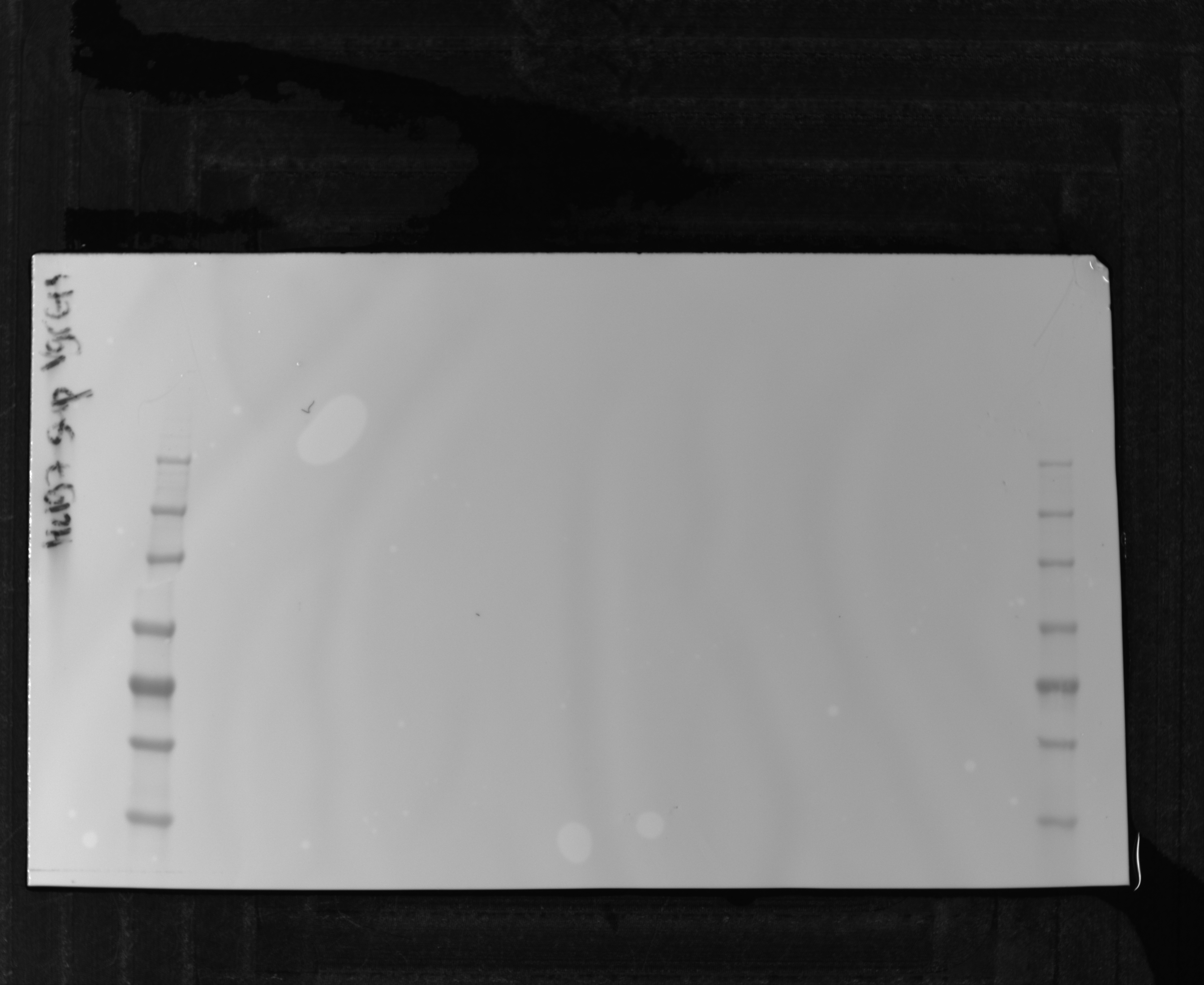

Supplement: Figure 3—figure supplement 1—source data 1. [file elife-82766-fig3-figsupp1-data1.zip › Figure 3-figure supplement 1b-source data 1/a-VgrG1/supernatant/06012021 a-VgrG1 sup WN 2 sec sup ladder.Tif]

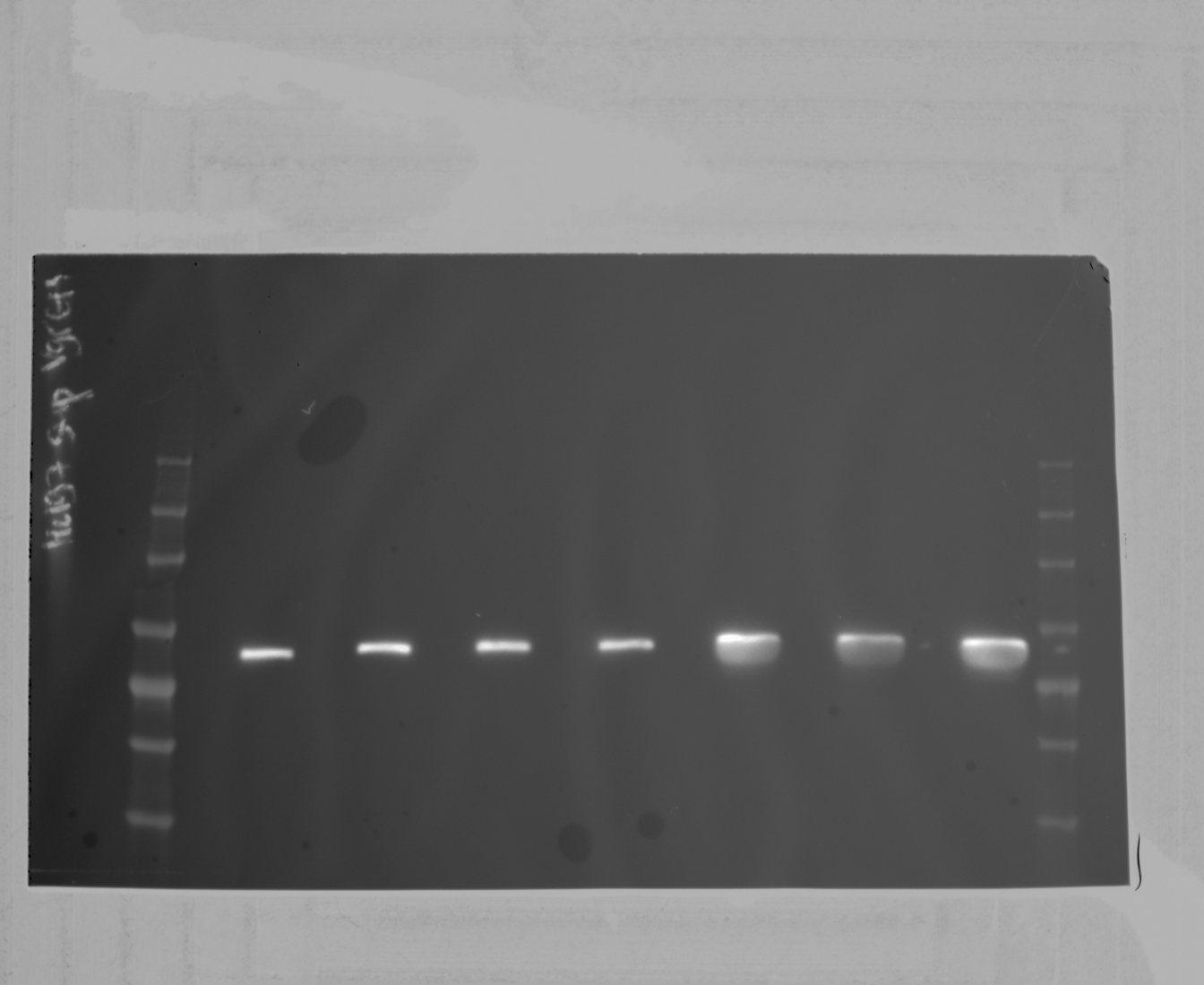

Supplement: Figure 3—figure supplement 1—source data 1. [file elife-82766-fig3-figsupp1-data1.zip › Figure 3-figure supplement 1b-source data 1/a-VgrG1/supernatant/06012021 a-VgrG1 sup WN 2 sec sup merge.Tif]

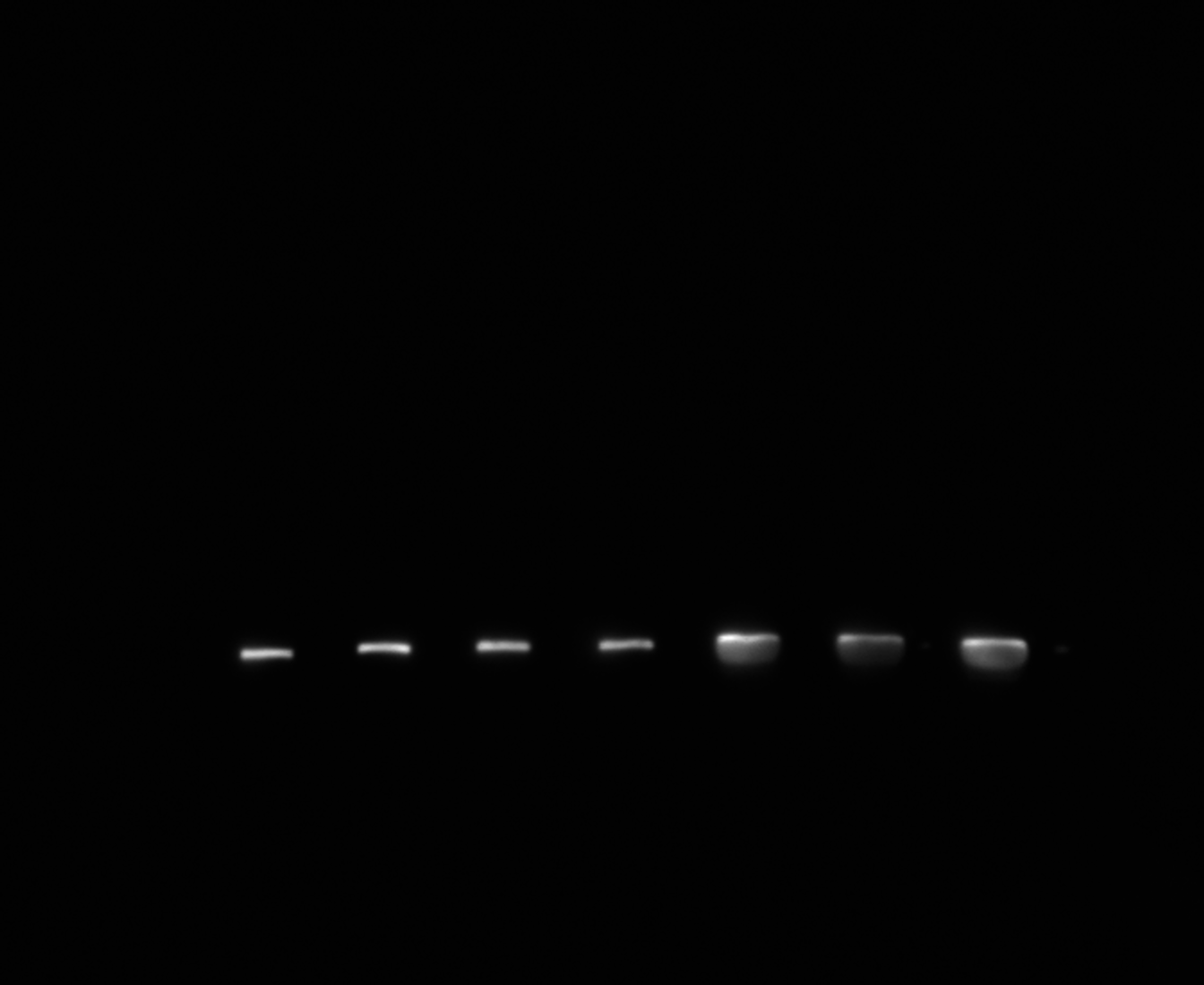

Supplement: Figure 3—figure supplement 1—source data 1. [file elife-82766-fig3-figsupp1-data1.zip › Figure 3-figure supplement 1b-source data 1/a-VgrG1/supernatant/06012021 a-VgrG1 sup WN 2 sec sup.Tif]

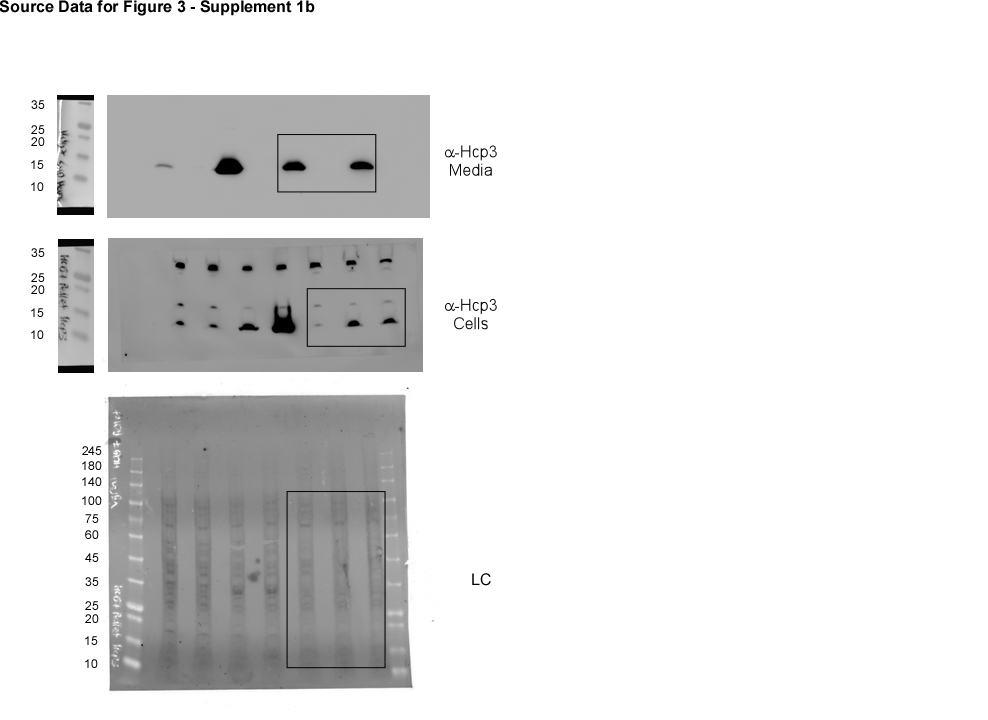

Supplement: Figure 3—figure supplement 1—source data 1. [file elife-82766-fig3-figsupp1-data1.zip › Figure 3-figure supplement 1b-source data 1/figure 3 - Supplement 1b.tif]

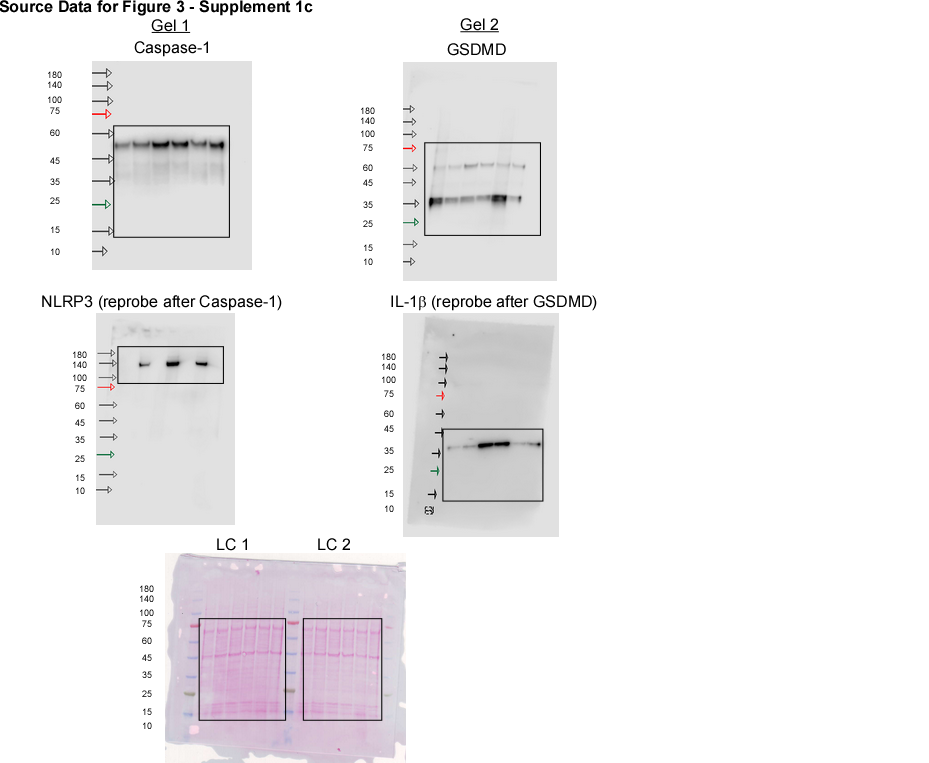

Supplement: Figure 3—figure supplement 1—source data 2. [file elife-82766-fig3-figsupp1-data2.zip › Figure 3-figure supplement 1c-source data 1/figure 3 - Supplement 1c.tif]

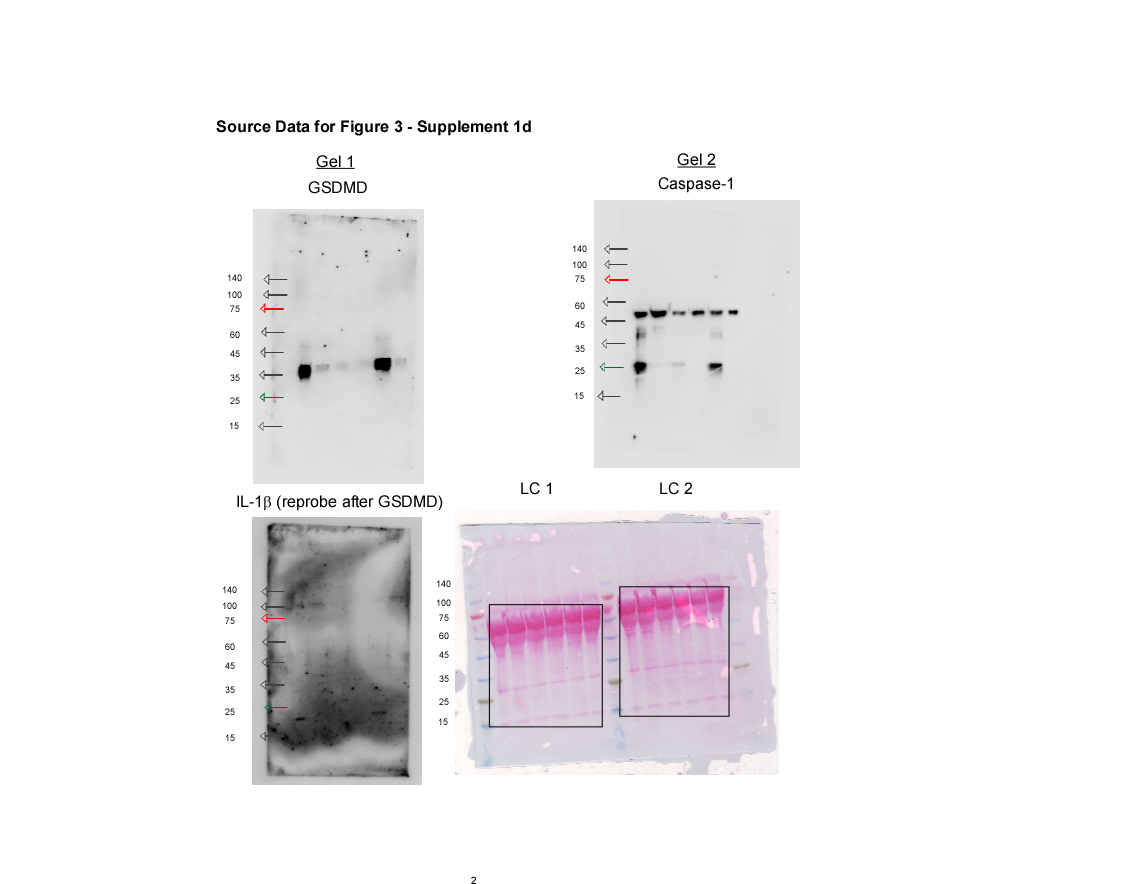

Supplement: Figure 3—figure supplement 1—source data 3. [file elife-82766-fig3-figsupp1-data3.zip › Figure 3-figure supplement 1d-source data 1/figure 3- supplement 1d.tif]

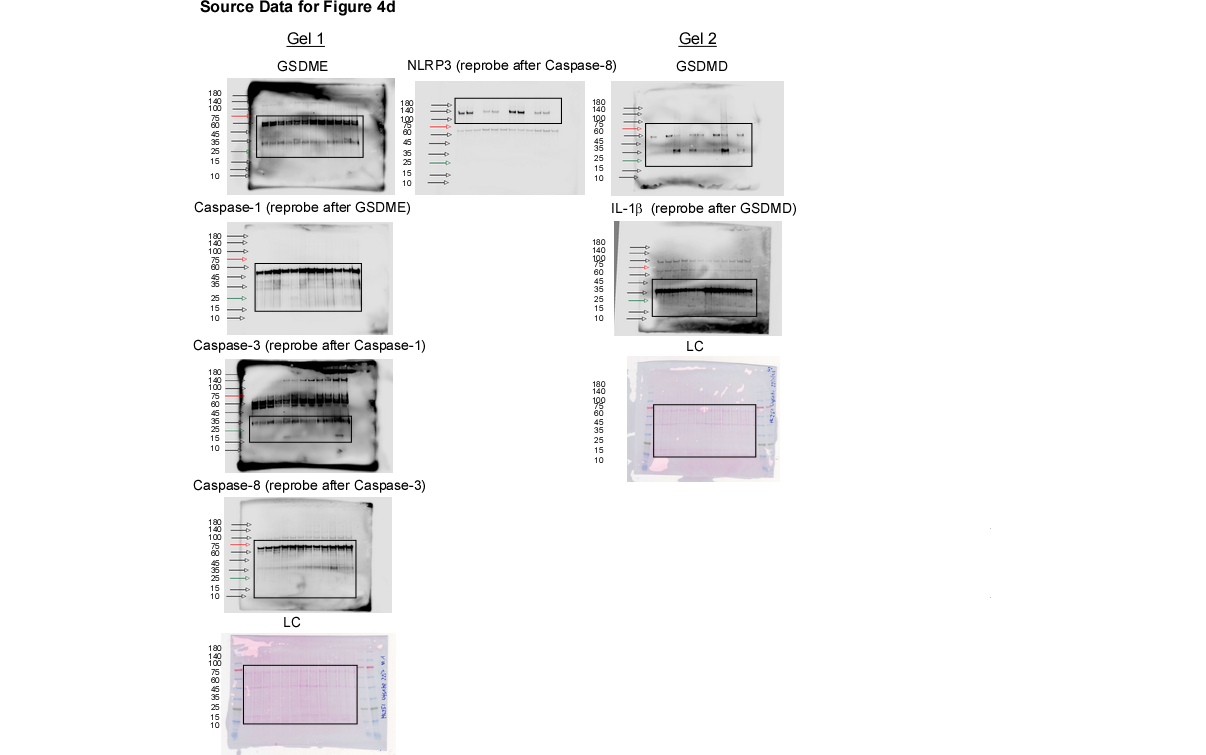

Supplement: Figure 4—source data 1. [file elife-82766-fig4-data1.zip › Figure 4d-source data 1/Figure 4d.tif]

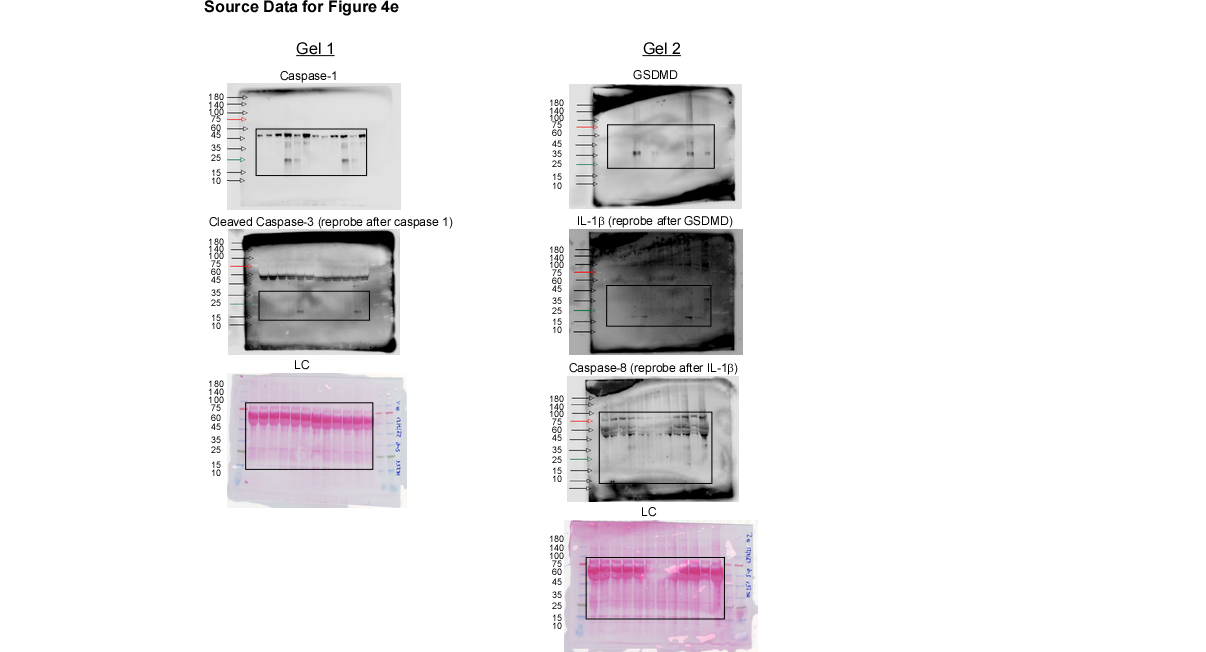

Supplement: Figure 4—source data 2. [file elife-82766-fig4-data2.zip › Figure 4e-source data 1/Figure 4e.tif]

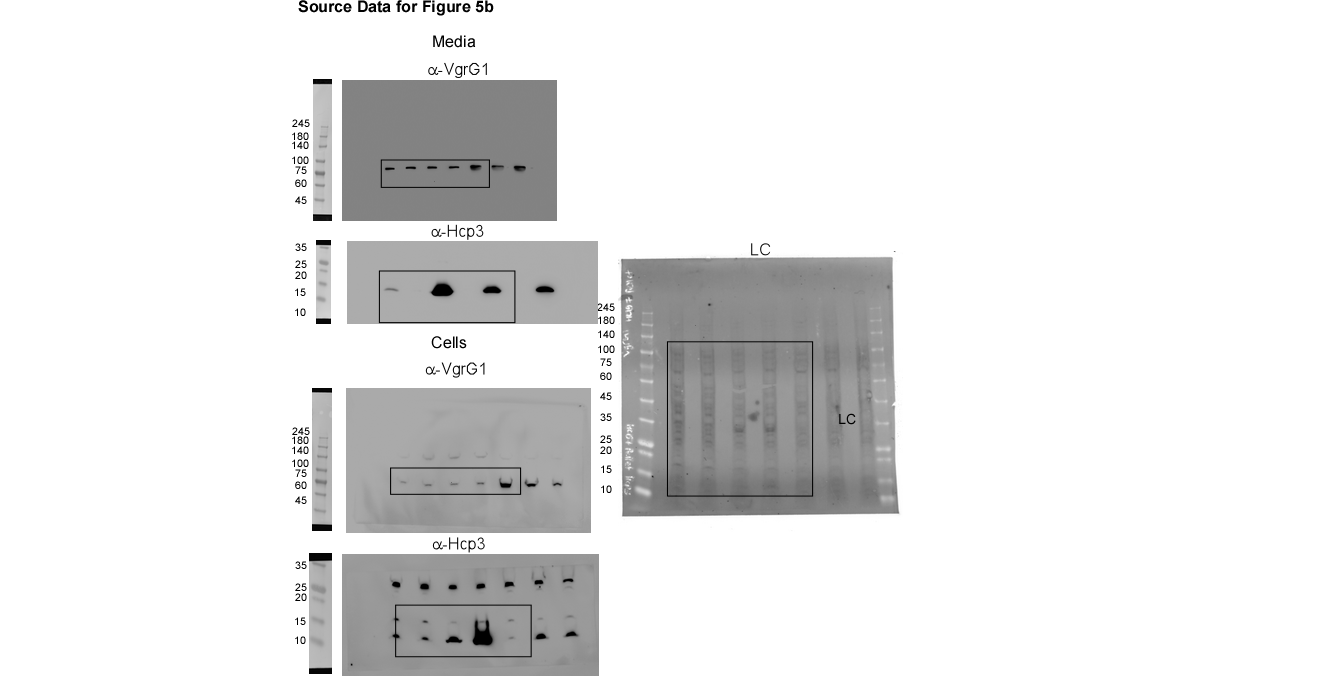

Supplement: Figure 5—source data 1. [file elife-82766-fig5-data1.zip › Figure 5b-source data 1/Figure 5b.tif]

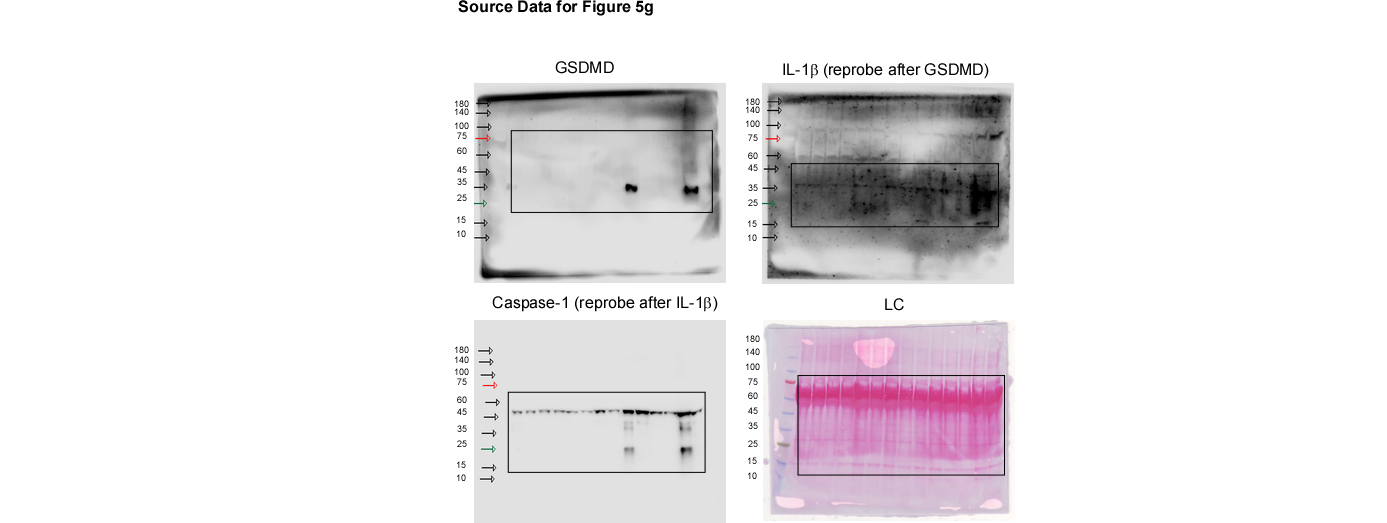

Supplement: Figure 5—source data 2. [file elife-82766-fig5-data2.zip › Figure 5g-source data 1/Figure 5g.tif]

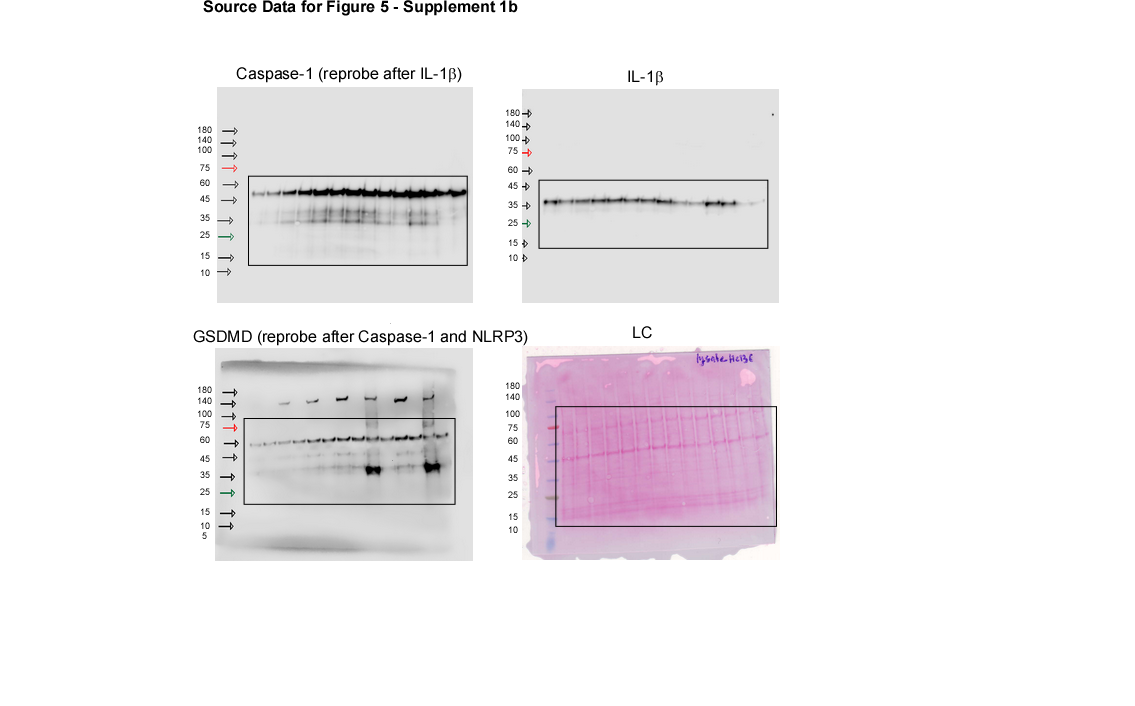

Supplement: Figure 5—figure supplement 1—source data 1. [file elife-82766-fig5-figsupp1-data1.zip › Figure 5-figure supplement 1b-source data 1/Figure 5 - supplement 1b.tif]

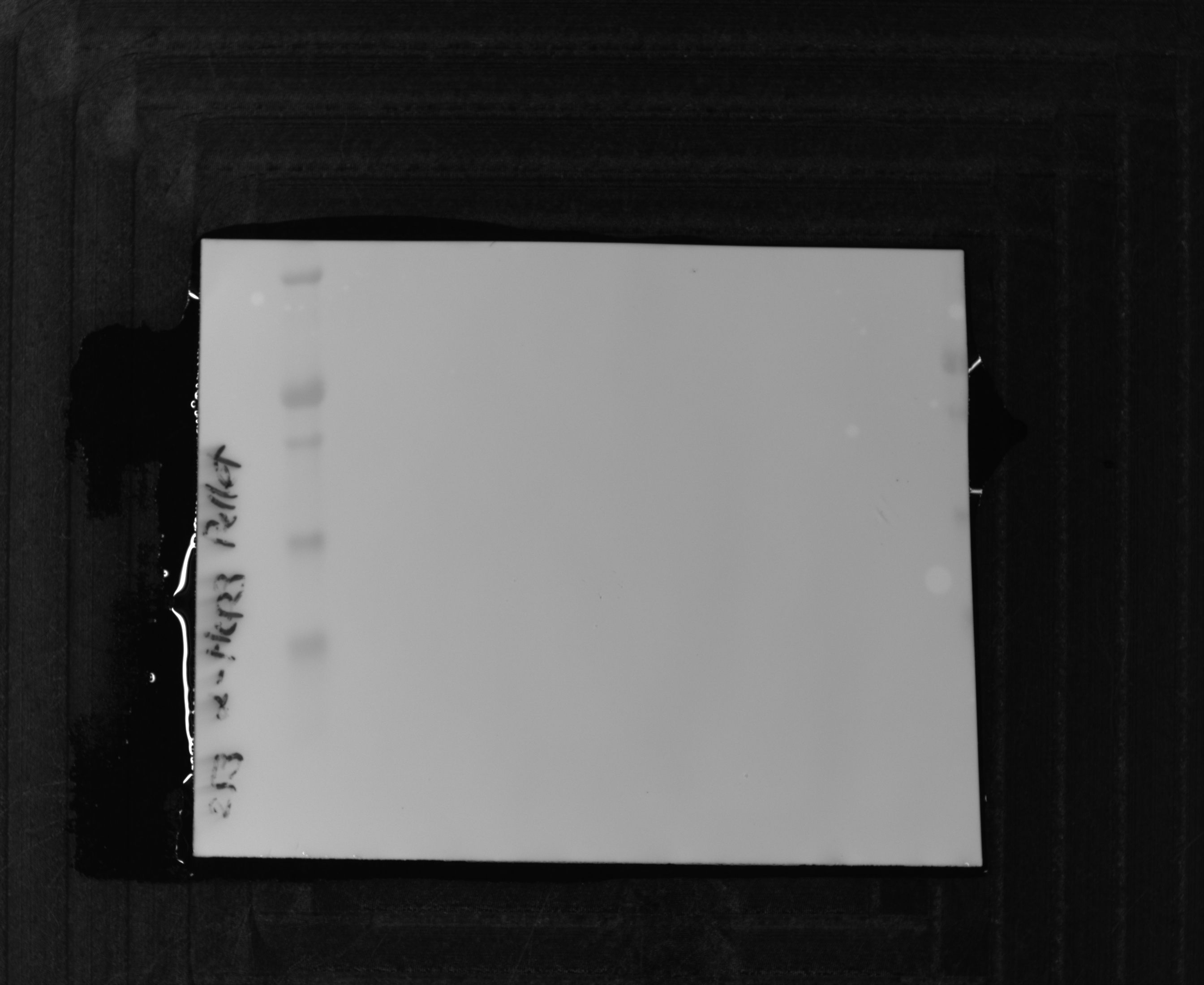

Supplement: Figure 7—source data 1. [file elife-82766-fig7-data1.zip › a-Hcp3/pellet/3-8-21 a-Hcp3#2 pellet CM 20 sec ladder.Tif]

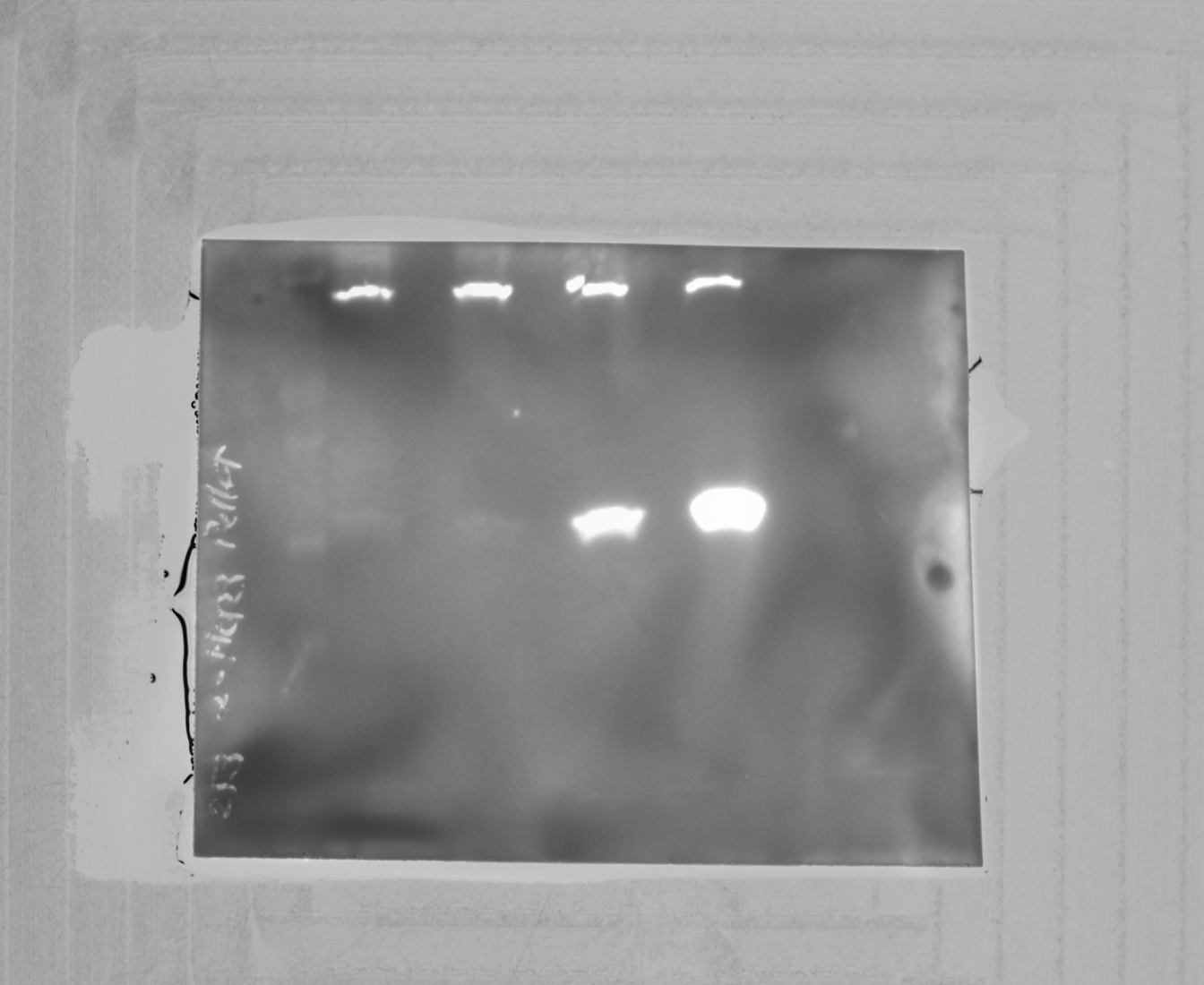

Supplement: Figure 7—source data 1. [file elife-82766-fig7-data1.zip › a-Hcp3/pellet/3-8-21 a-Hcp3#2 pellet CM 20 sec merge.Tif]

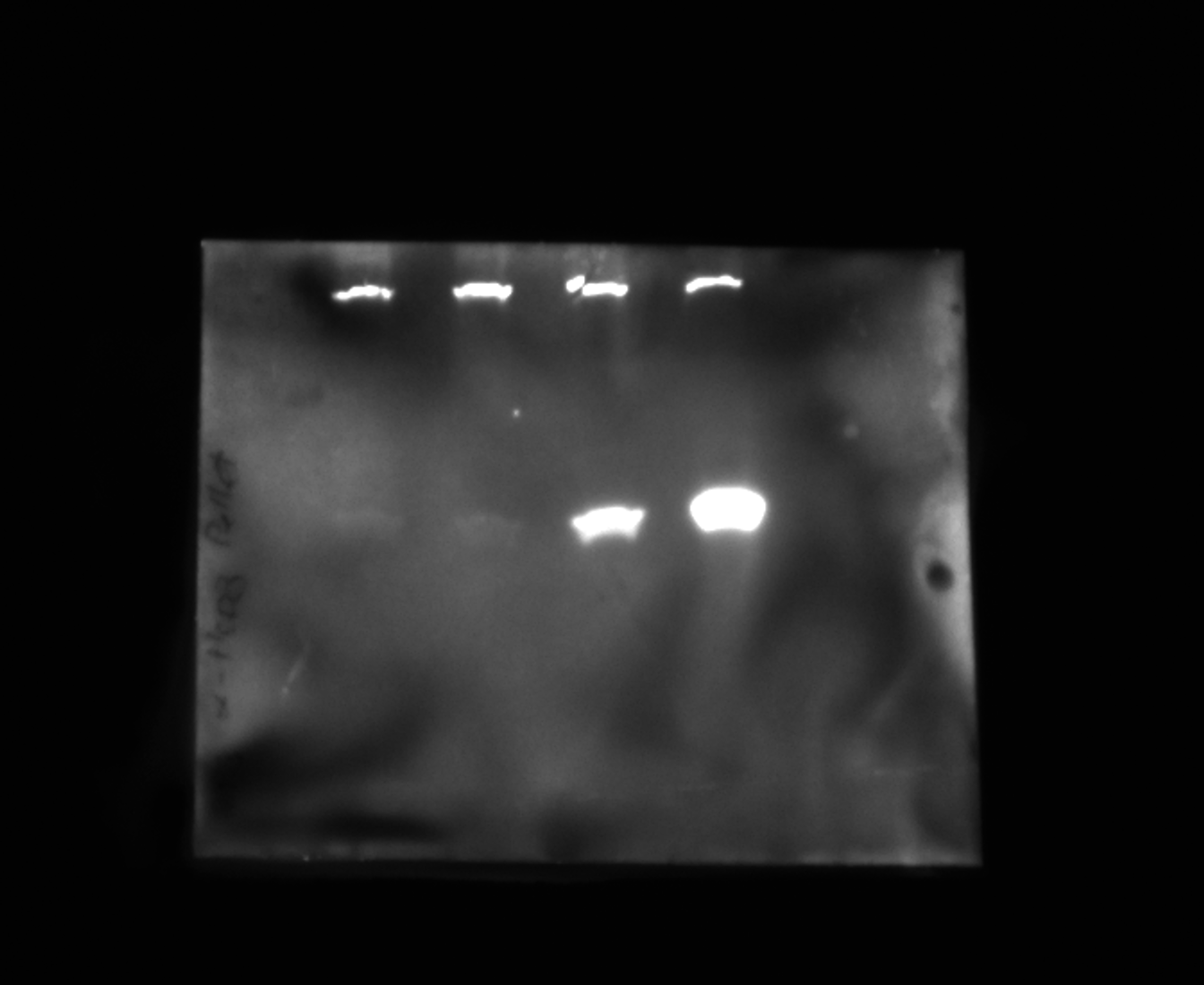

Supplement: Figure 7—source data 1. [file elife-82766-fig7-data1.zip › a-Hcp3/pellet/3-8-21 a-Hcp3#2 pellet CM 20 sec.Tif]

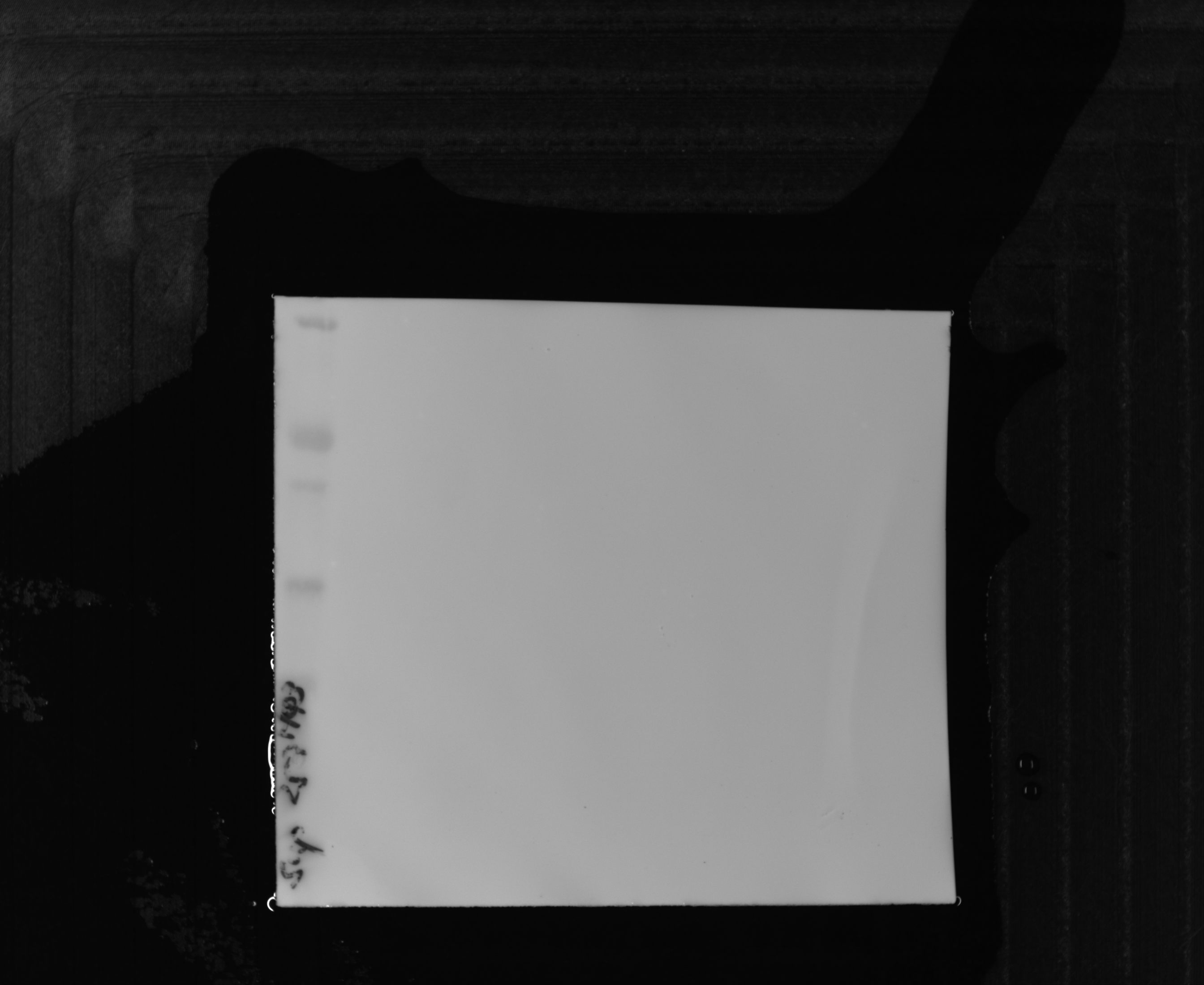

Supplement: Figure 7—source data 1. [file elife-82766-fig7-data1.zip › a-Hcp3/supernatant/3-8-21 a-Hcp3#2 sup WN 5 sec ladder.Tif]

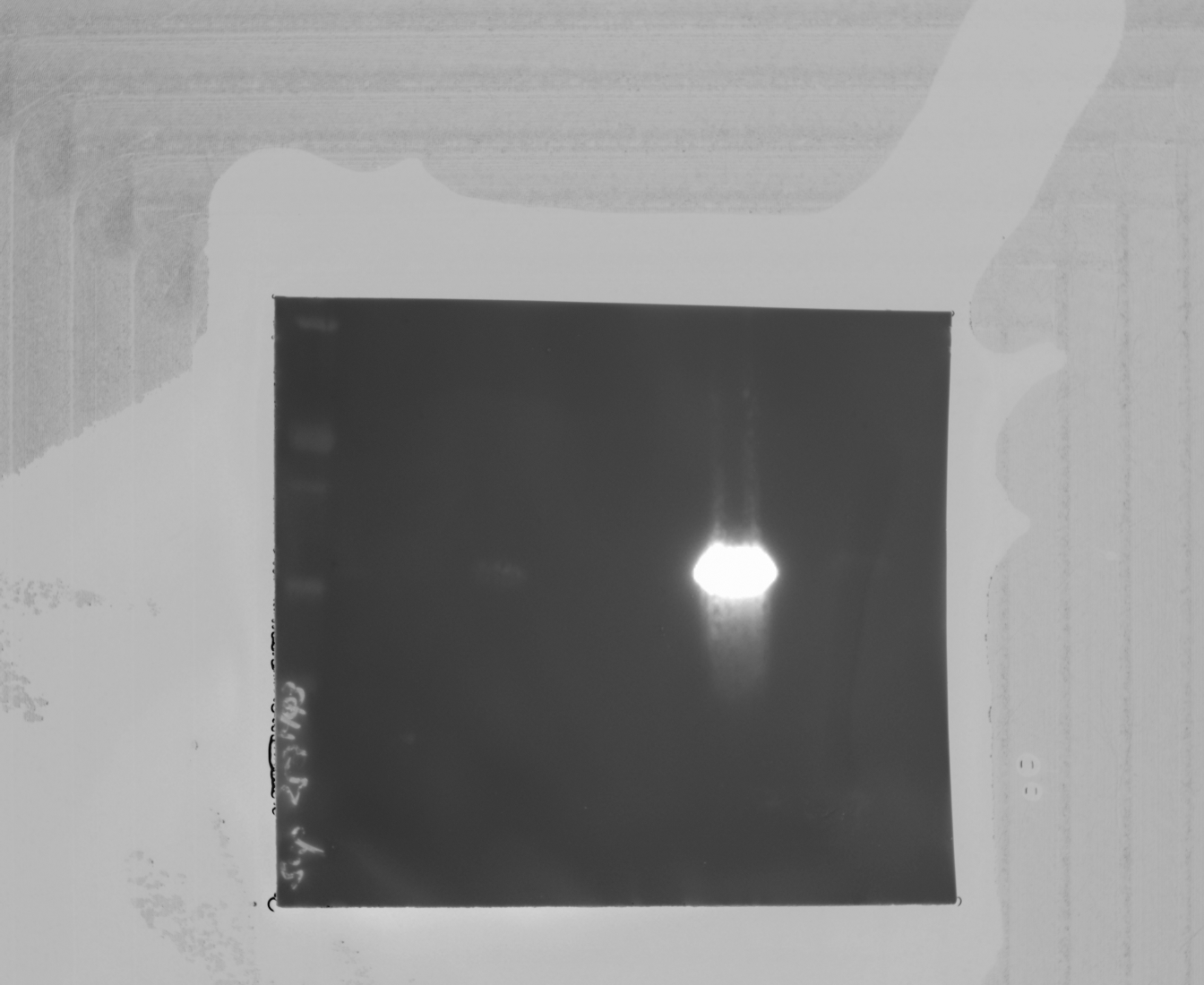

Supplement: Figure 7—source data 1. [file elife-82766-fig7-data1.zip › a-Hcp3/supernatant/3-8-21 a-Hcp3#2 sup WN 5 sec merge.Tif]

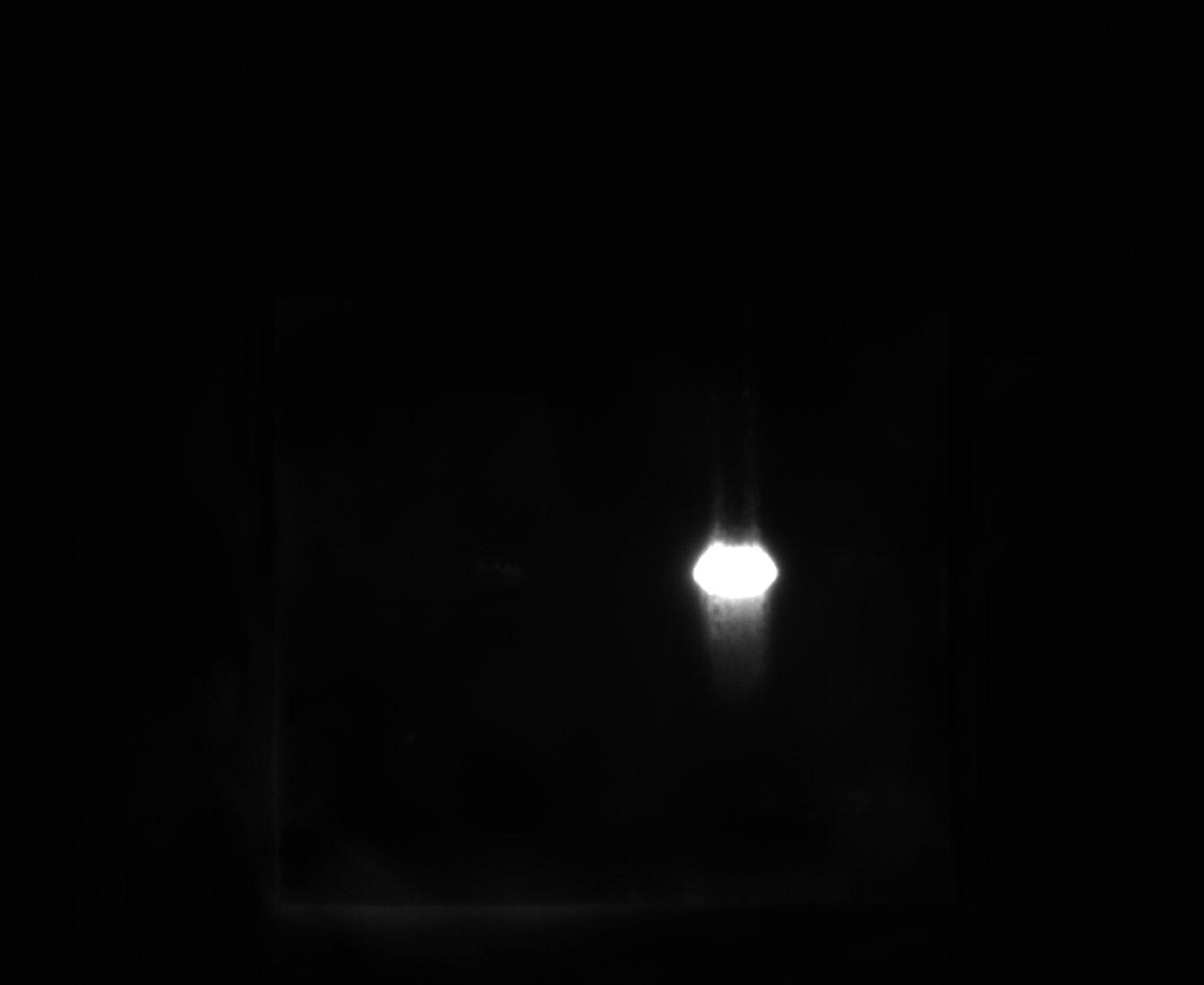

Supplement: Figure 7—source data 1. [file elife-82766-fig7-data1.zip › a-Hcp3/supernatant/3-8-21 a-Hcp3#2 sup WN 5 sec.Tif]

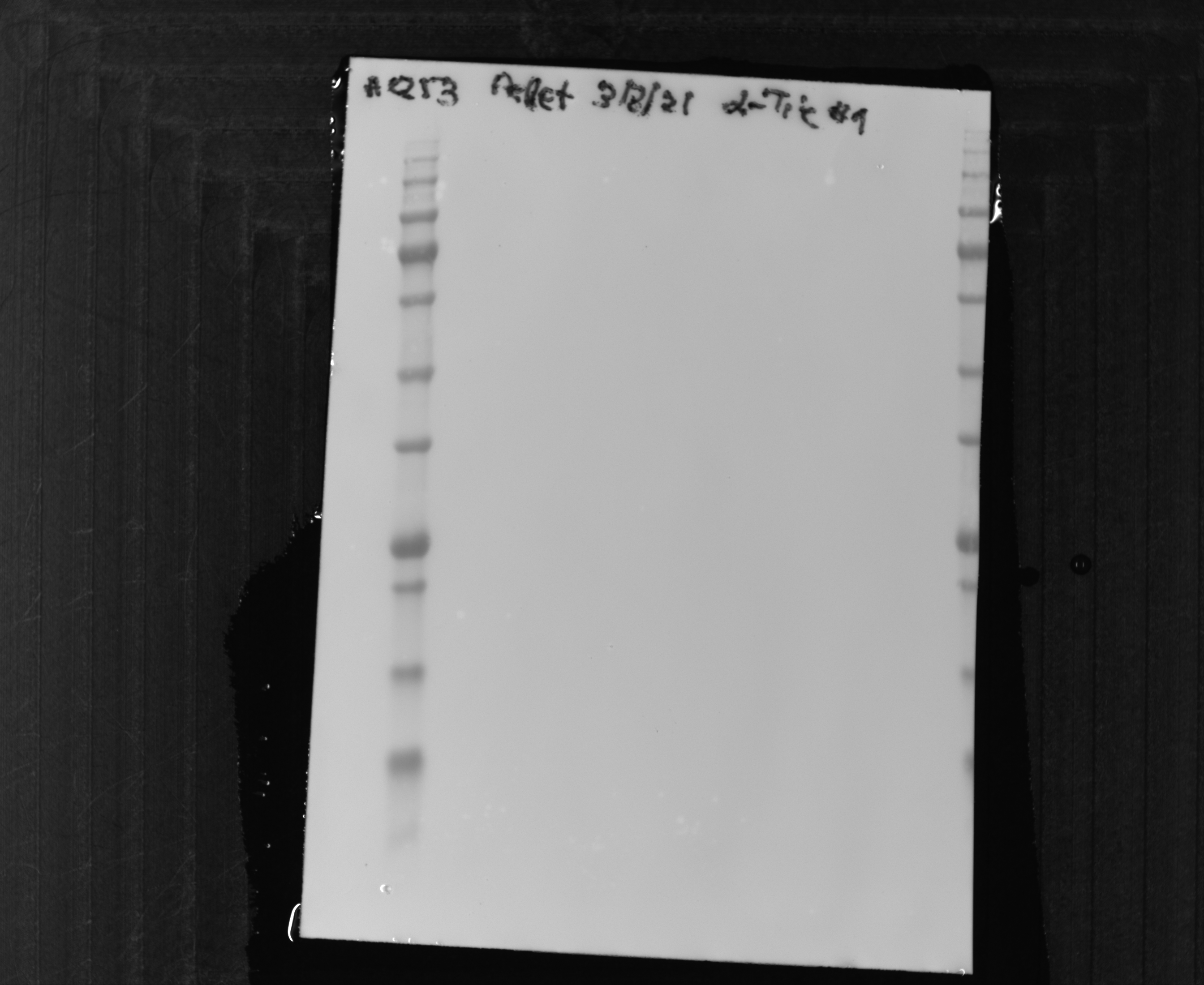

Supplement: Figure 7—source data 2. [file elife-82766-fig7-data2.zip › a-Tie1/pellet/3-8-21 a-Tie1 #2 pellet CM 10 sec ladder.Tif]

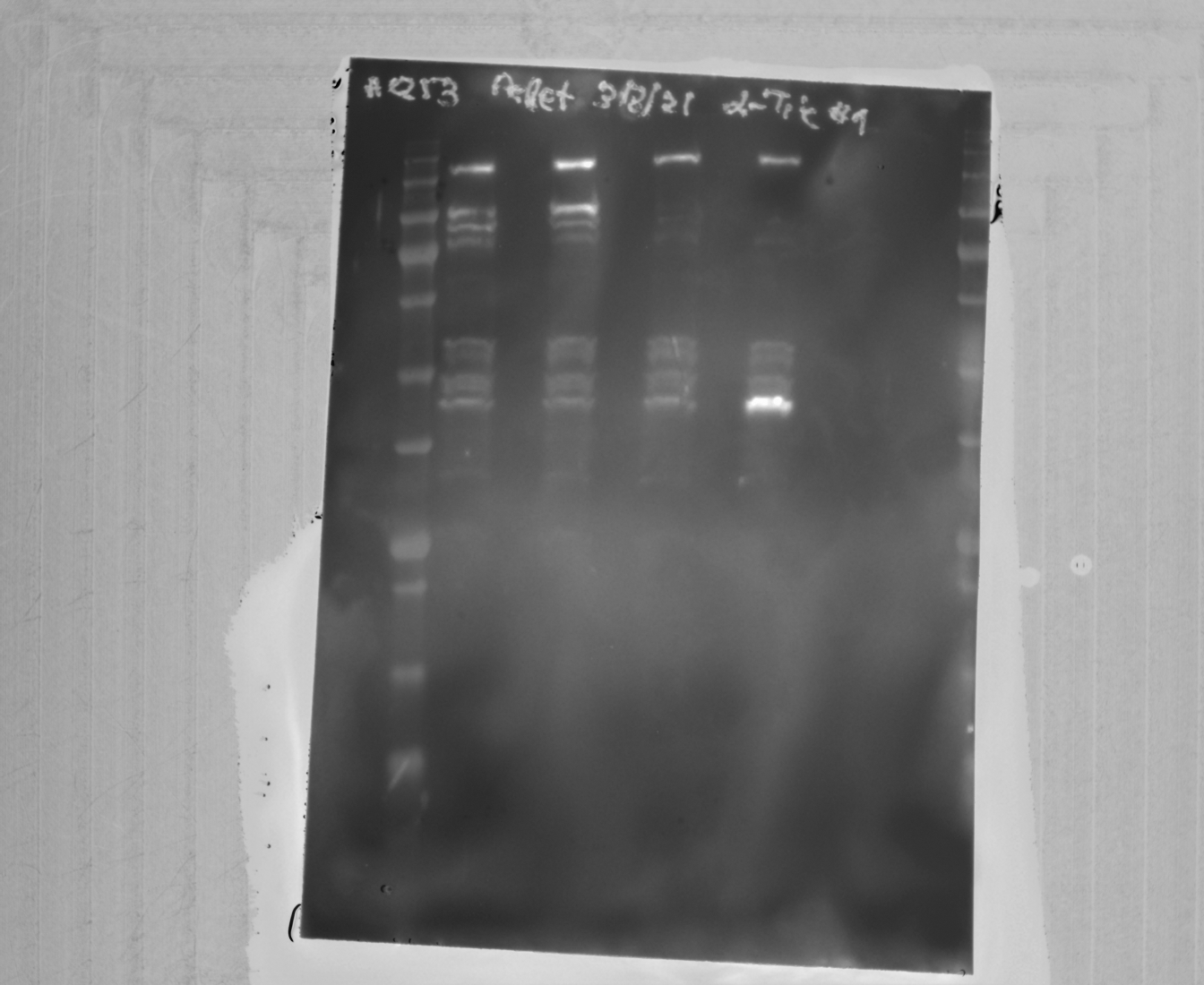

Supplement: Figure 7—source data 2. [file elife-82766-fig7-data2.zip › a-Tie1/pellet/3-8-21 a-Tie1 #2 pellet CM 10 sec merge.Tif]

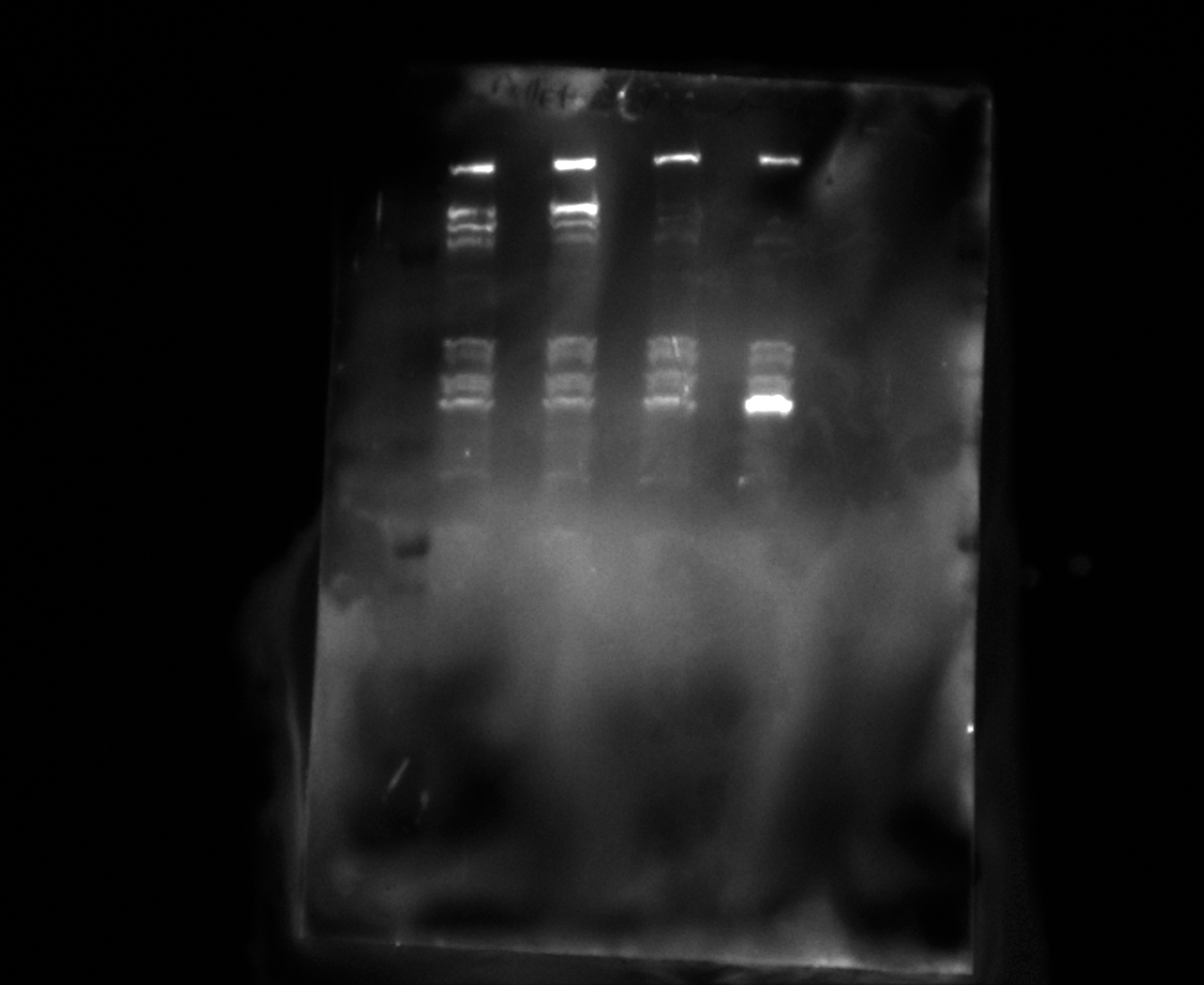

Supplement: Figure 7—source data 2. [file elife-82766-fig7-data2.zip › a-Tie1/pellet/3-8-21 a-Tie1 #2 pellet CM 10 sec.Tif]

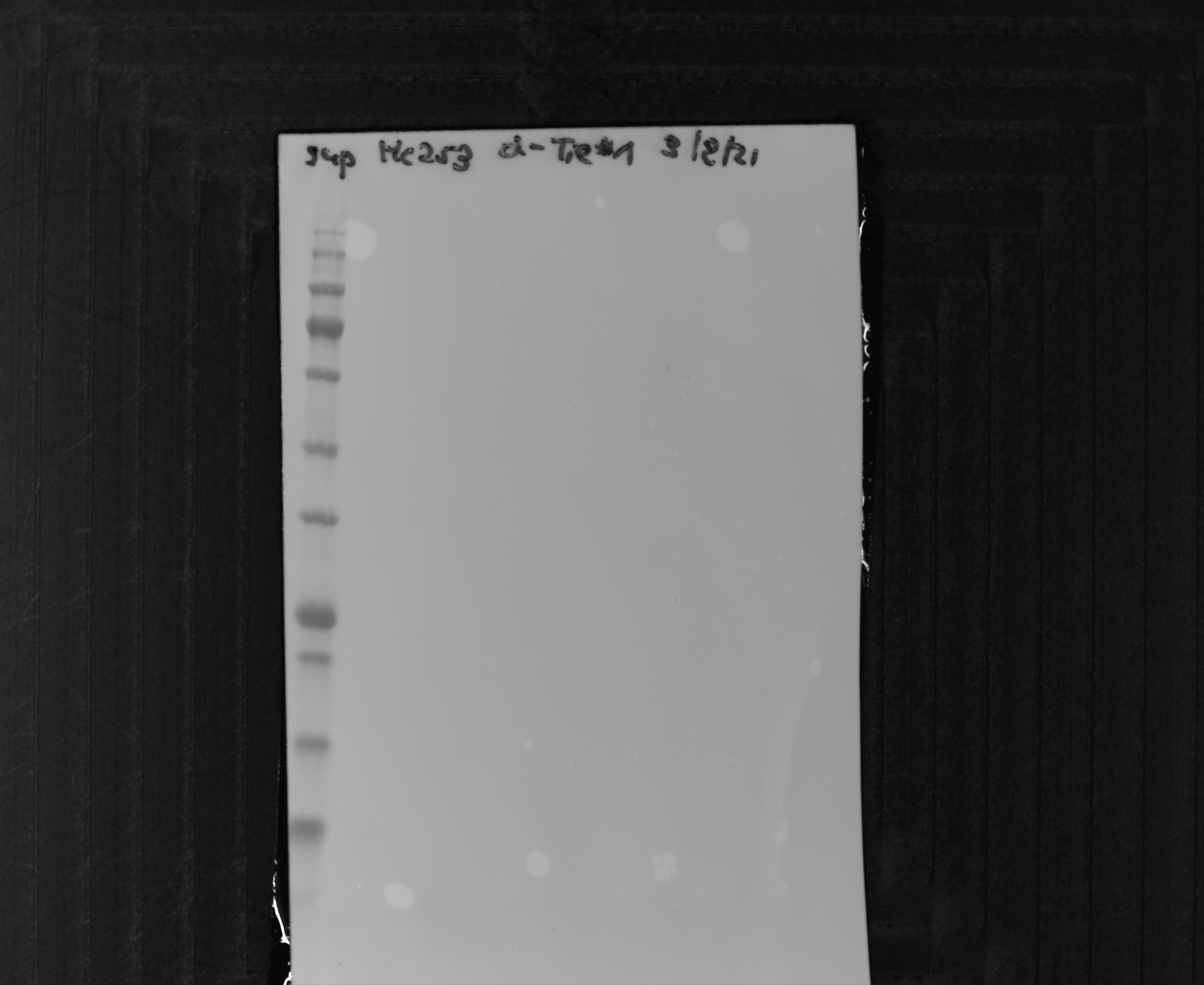

Supplement: Figure 7—source data 2. [file elife-82766-fig7-data2.zip › a-Tie1/supernatant/3-8-21 a-Tie1 #2 sup CM 4 sec ladder.Tif]

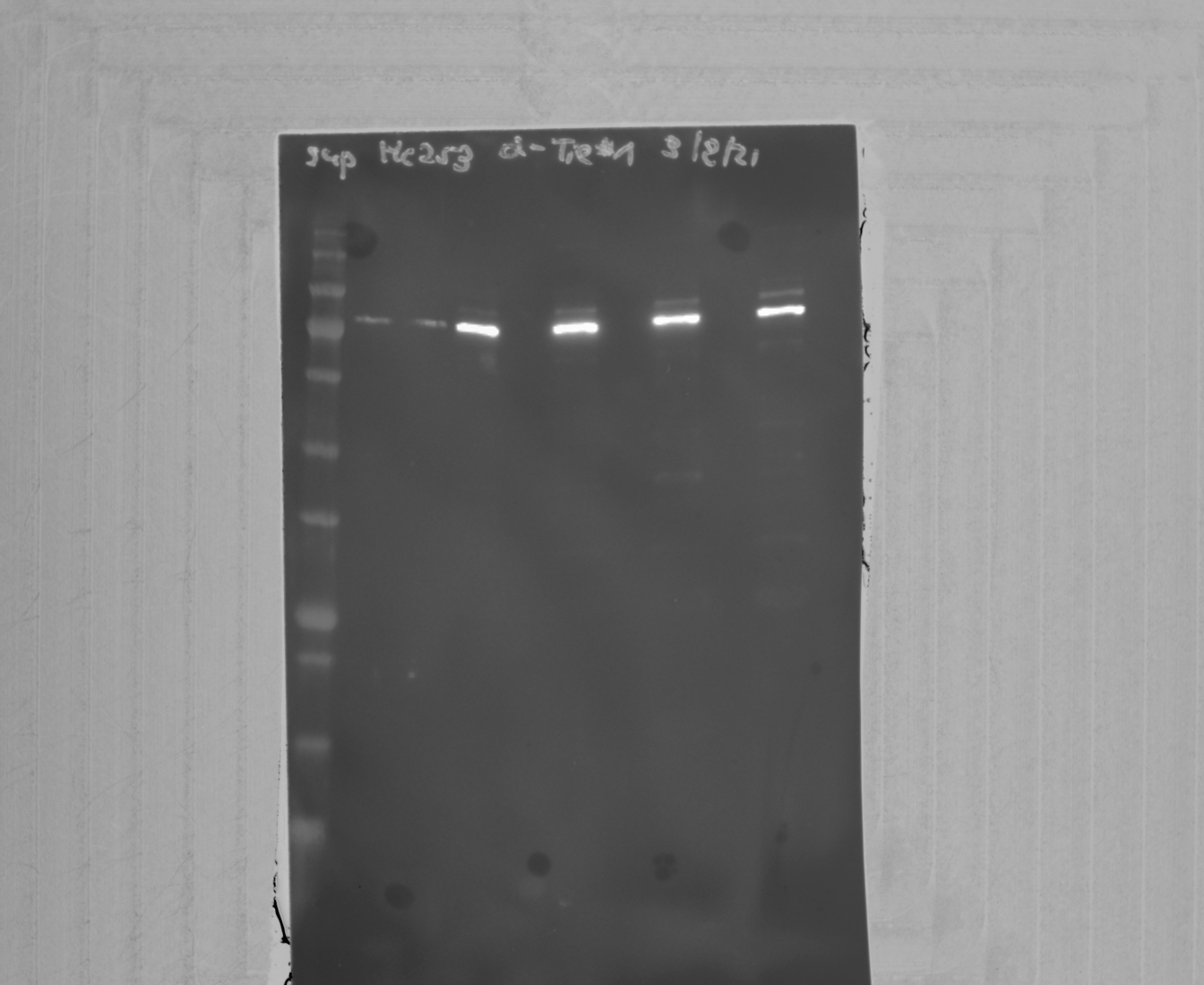

Supplement: Figure 7—source data 2. [file elife-82766-fig7-data2.zip › a-Tie1/supernatant/3-8-21 a-Tie1 #2 sup CM 4 sec merge.Tif]

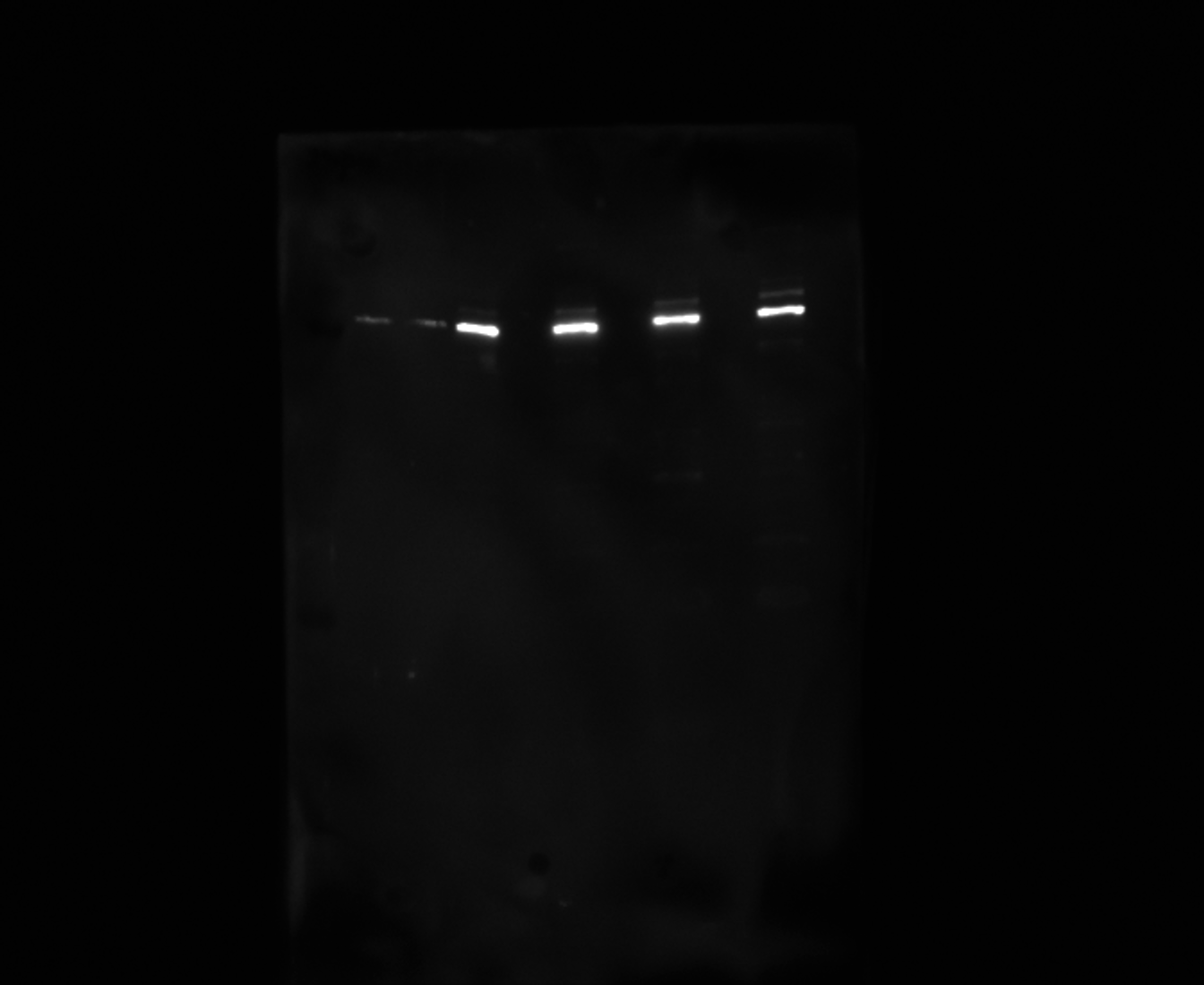

Supplement: Figure 7—source data 2. [file elife-82766-fig7-data2.zip › a-Tie1/supernatant/3-8-21 a-Tie1 #2 sup CM 4 sec.Tif]

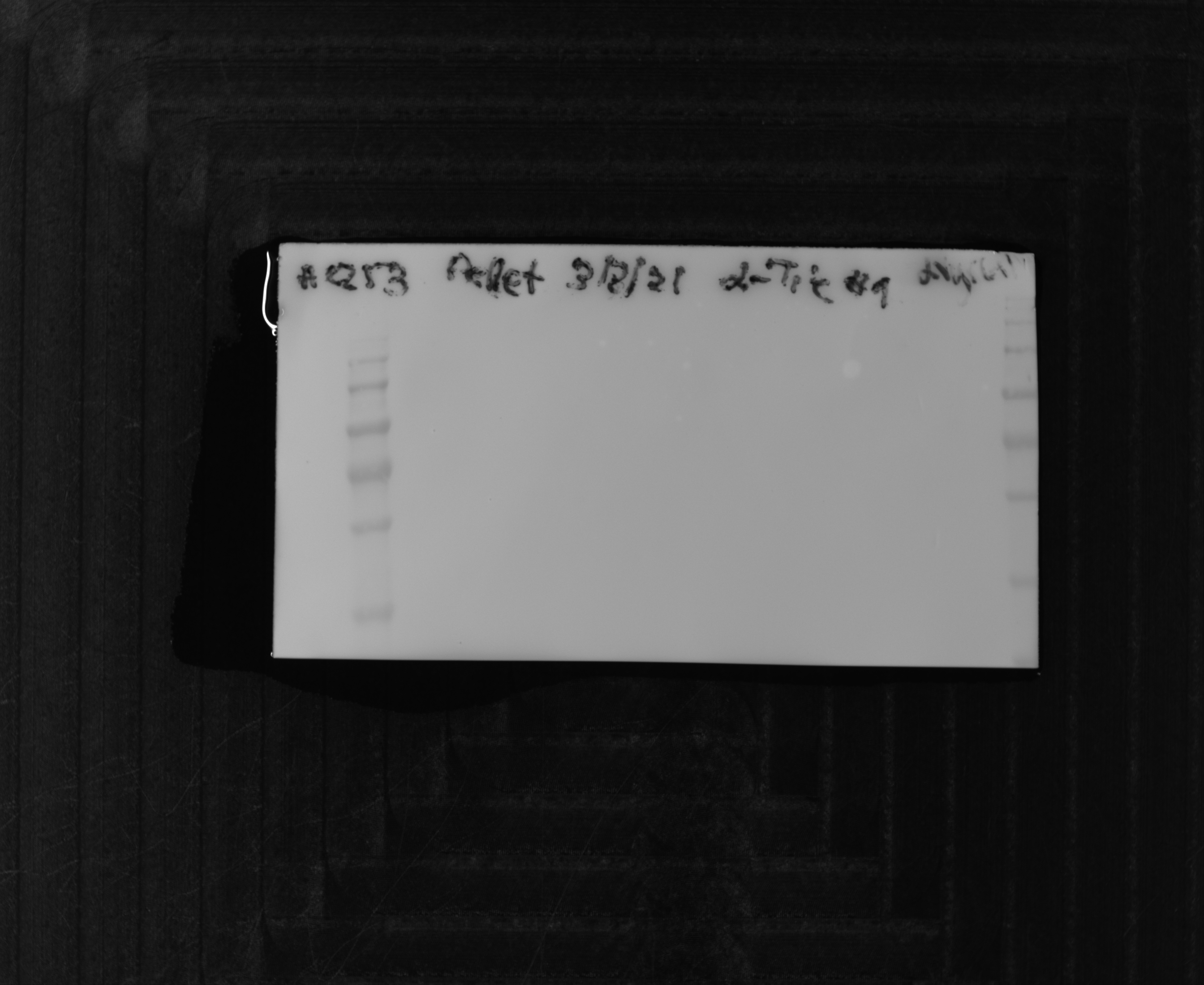

Supplement: Figure 7—source data 3. [file elife-82766-fig7-data3.zip › a-Tie2/pellet/30-11-21 a-Tie2 pellet wn1 2 sec ladder.Tif]

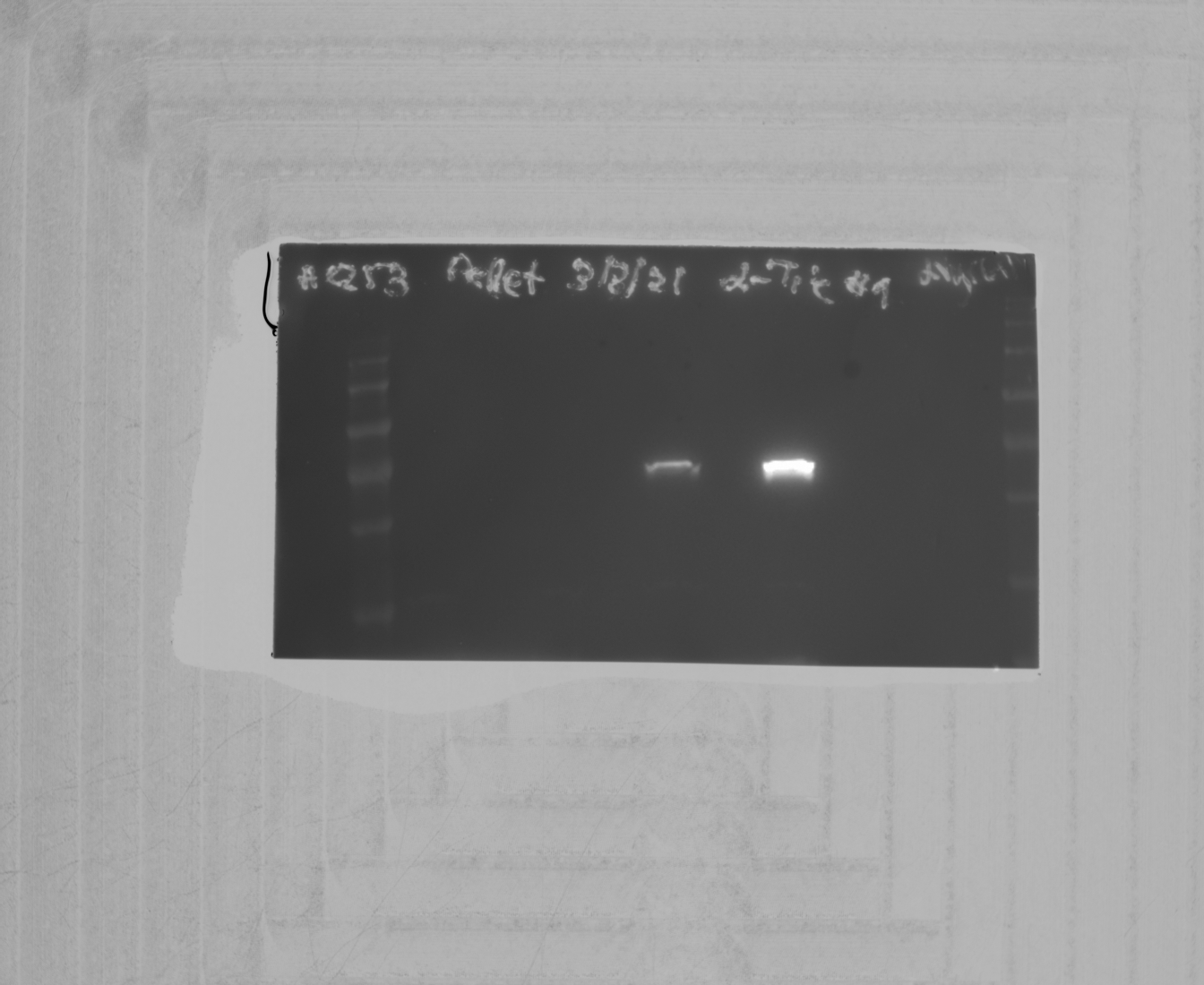

Supplement: Figure 7—source data 3. [file elife-82766-fig7-data3.zip › a-Tie2/pellet/30-11-21 a-Tie2 pellet wn1 2 sec merge.Tif]

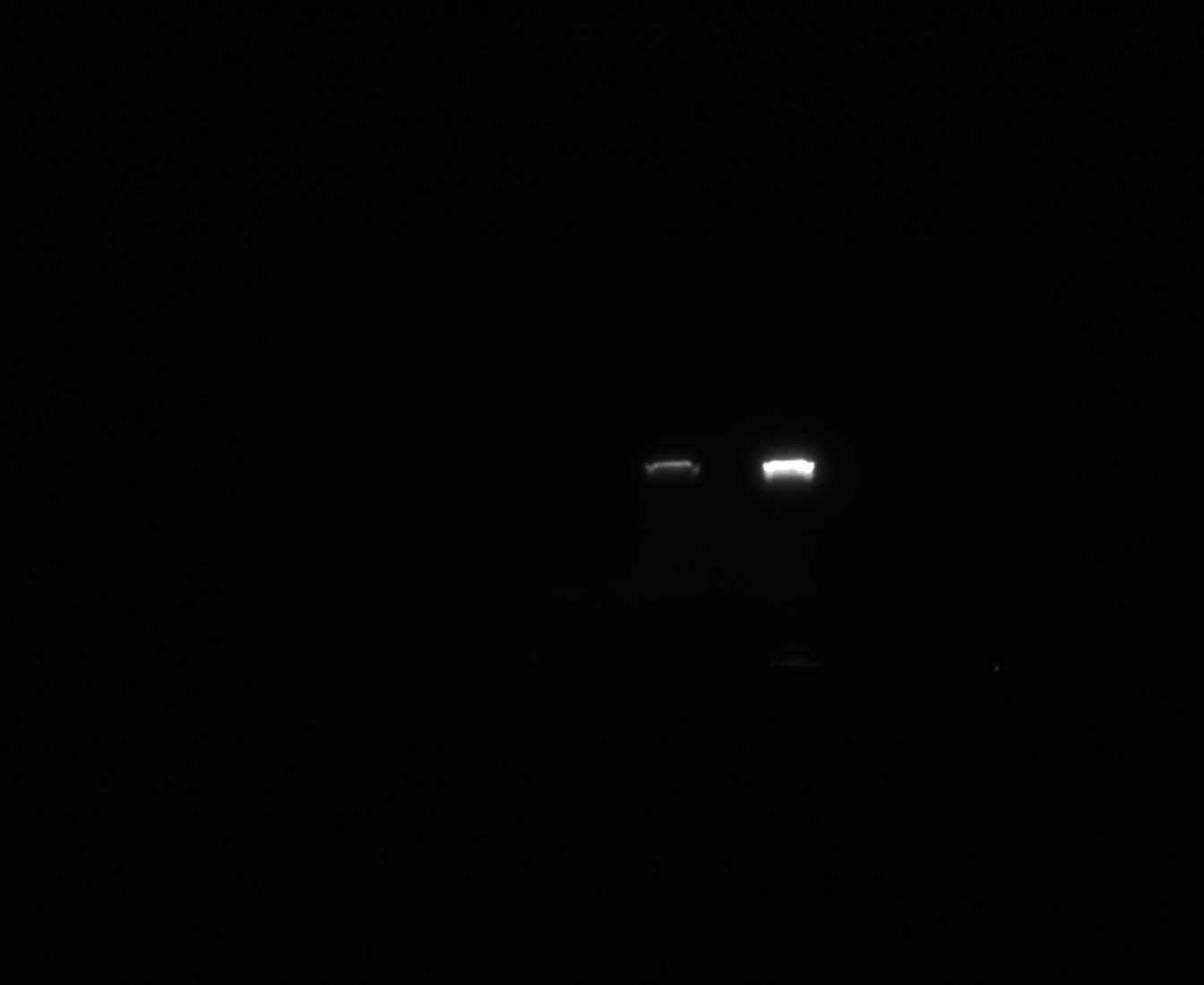

Supplement: Figure 7—source data 3. [file elife-82766-fig7-data3.zip › a-Tie2/pellet/30-11-21 a-Tie2 pellet wn1 2 sec.Tif]

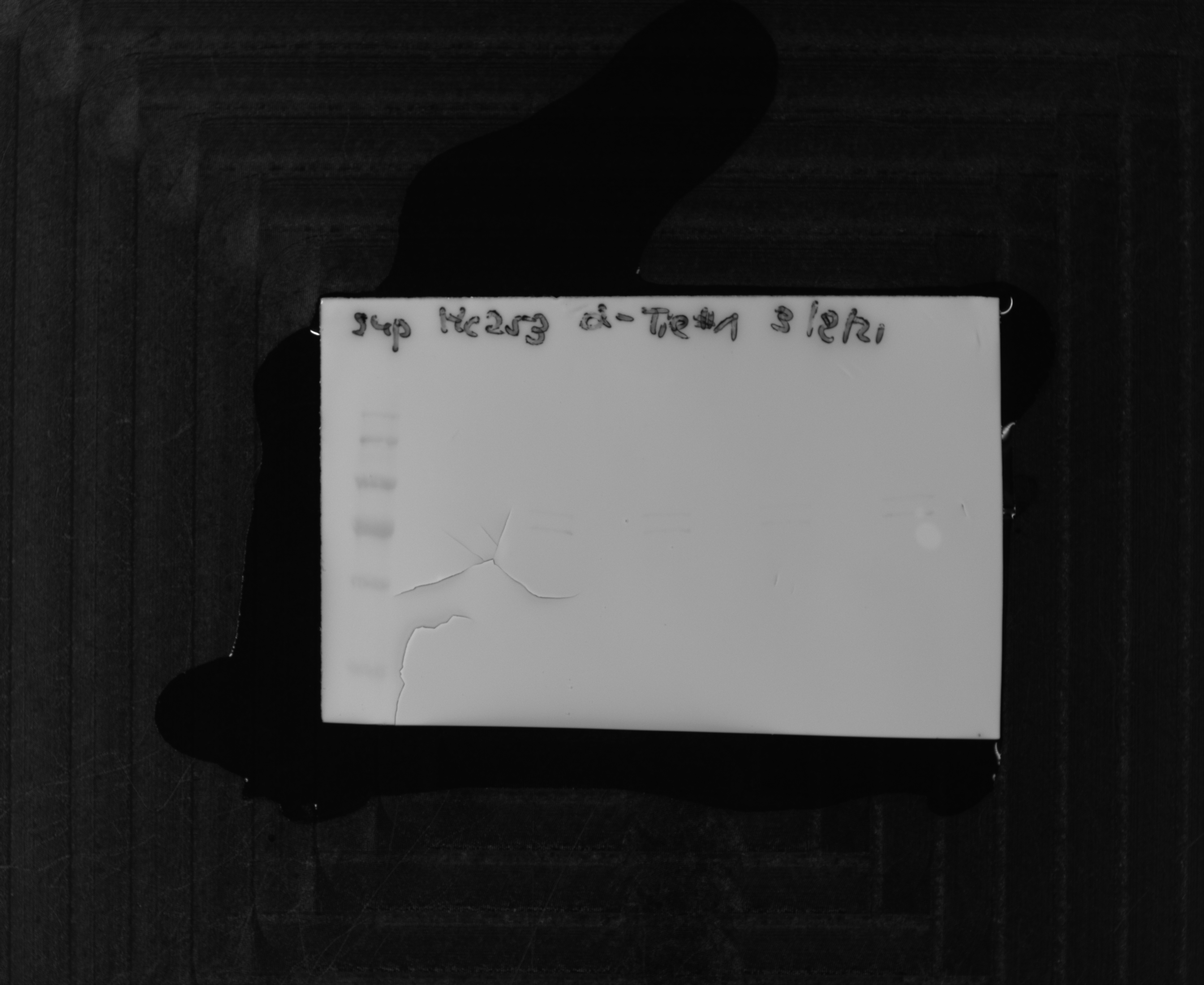

Supplement: Figure 7—source data 3. [file elife-82766-fig7-data3.zip › a-Tie2/supernatant/30-11-21 a-Tie2 sup wn 2 sec ladder.Tif]

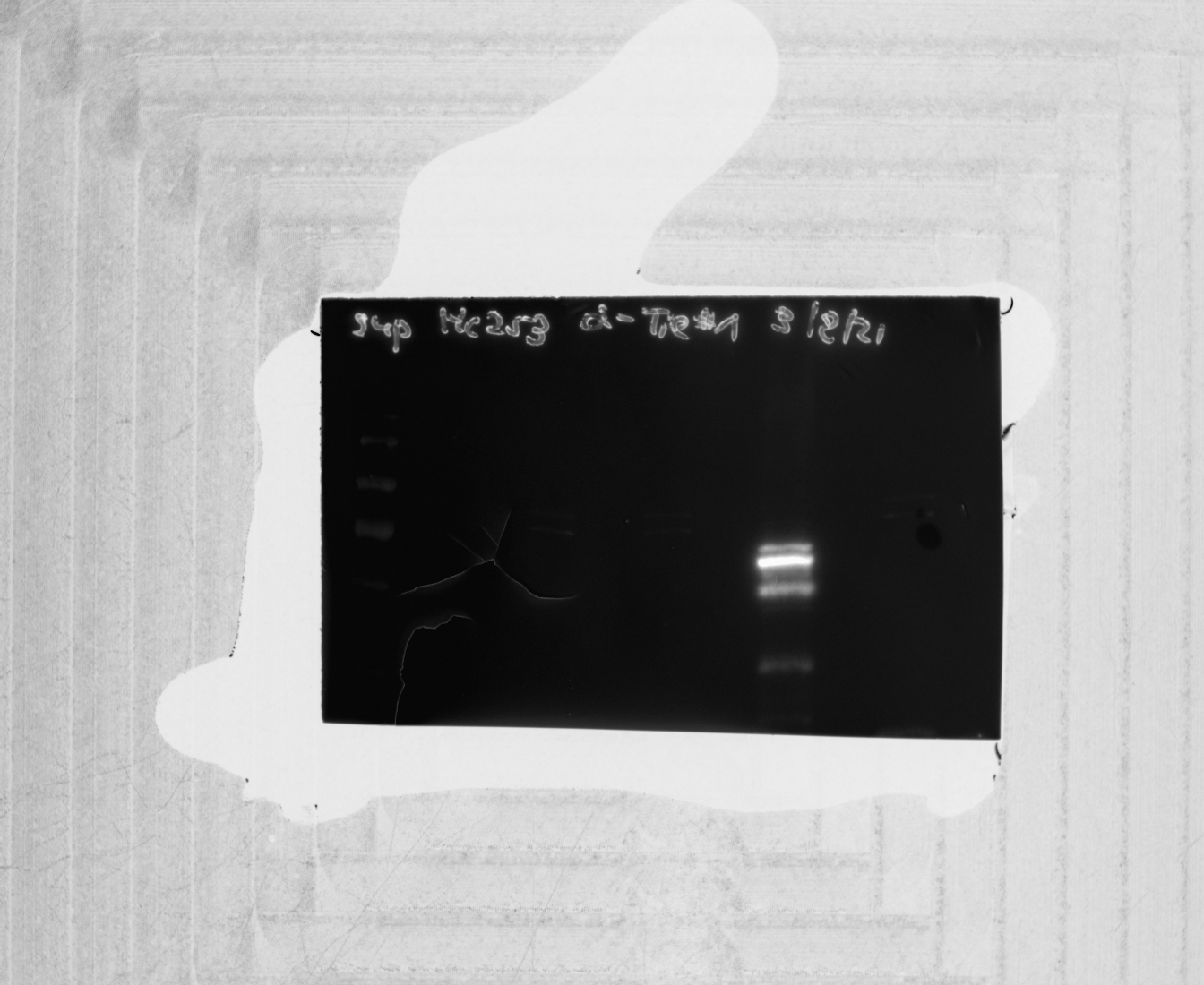

Supplement: Figure 7—source data 3. [file elife-82766-fig7-data3.zip › a-Tie2/supernatant/30-11-21 a-Tie2 sup wn 2 sec merge.Tif]

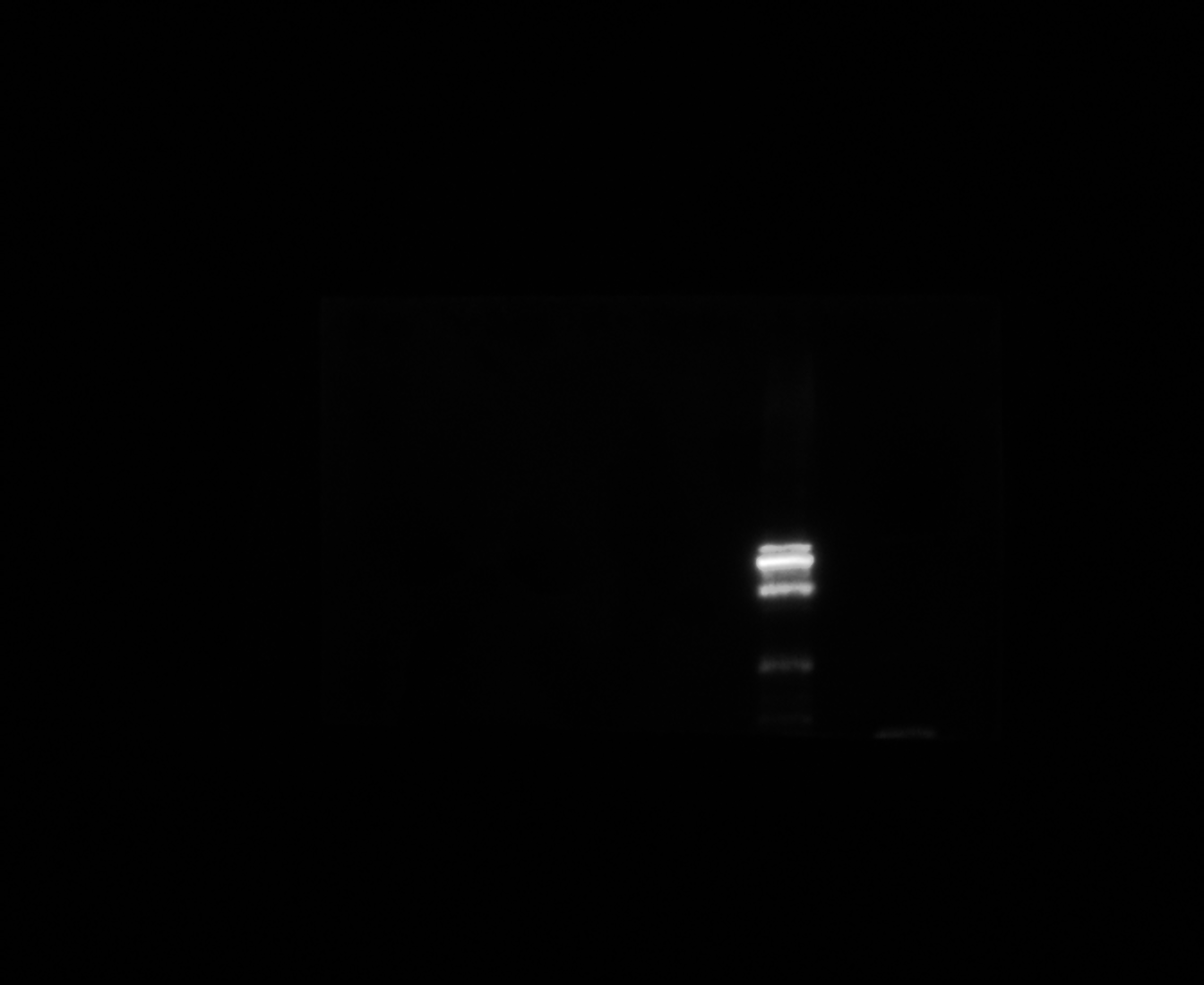

Supplement: Figure 7—source data 3. [file elife-82766-fig7-data3.zip › a-Tie2/supernatant/30-11-21 a-Tie2 sup wn 2 sec.Tif]

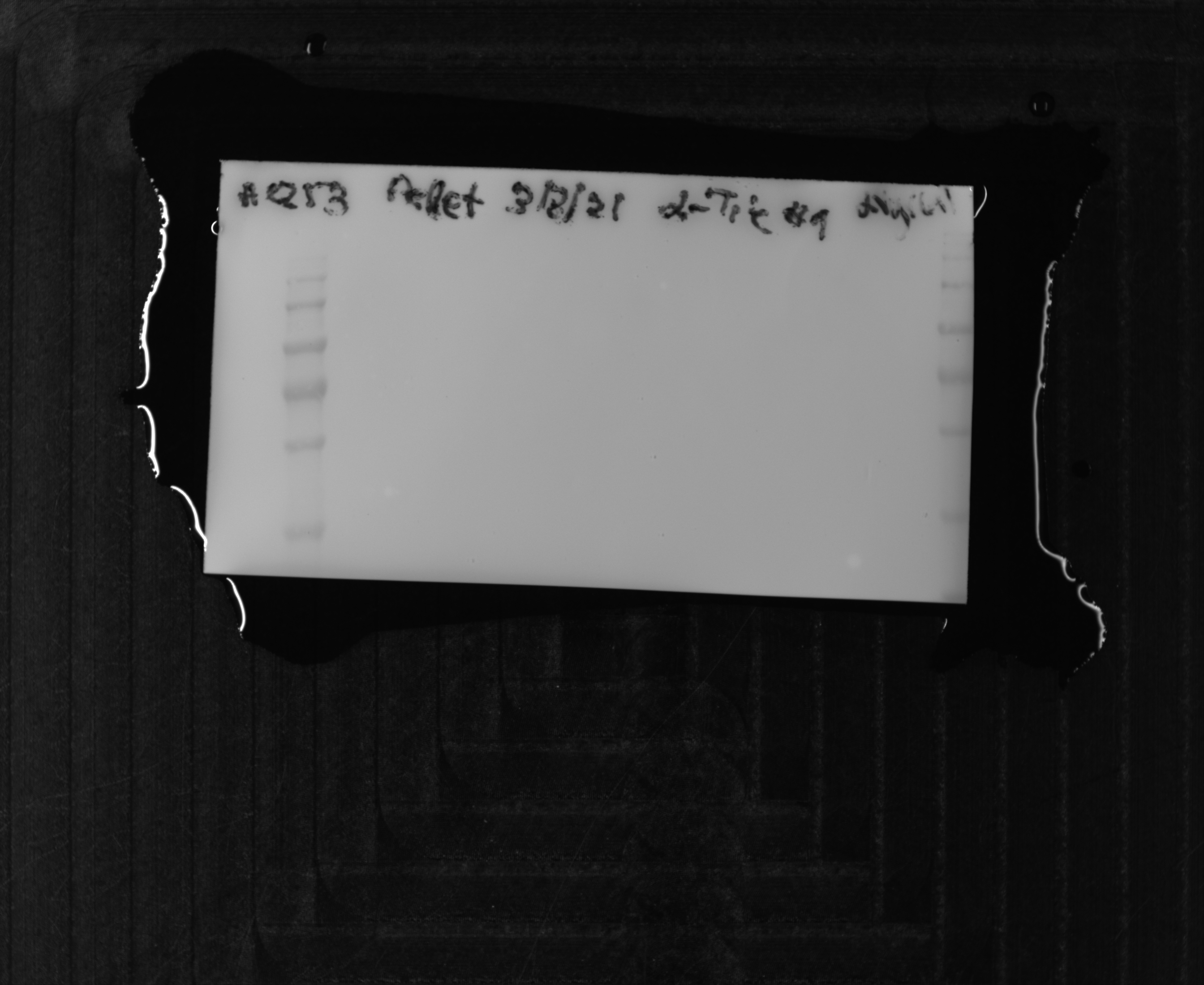

Supplement: Figure 7—source data 4. [file elife-82766-fig7-data4.zip › a-VgrG1/pellet/4-8-21 a-vgrg1 pellet CM 5 sec ladder.Tif]

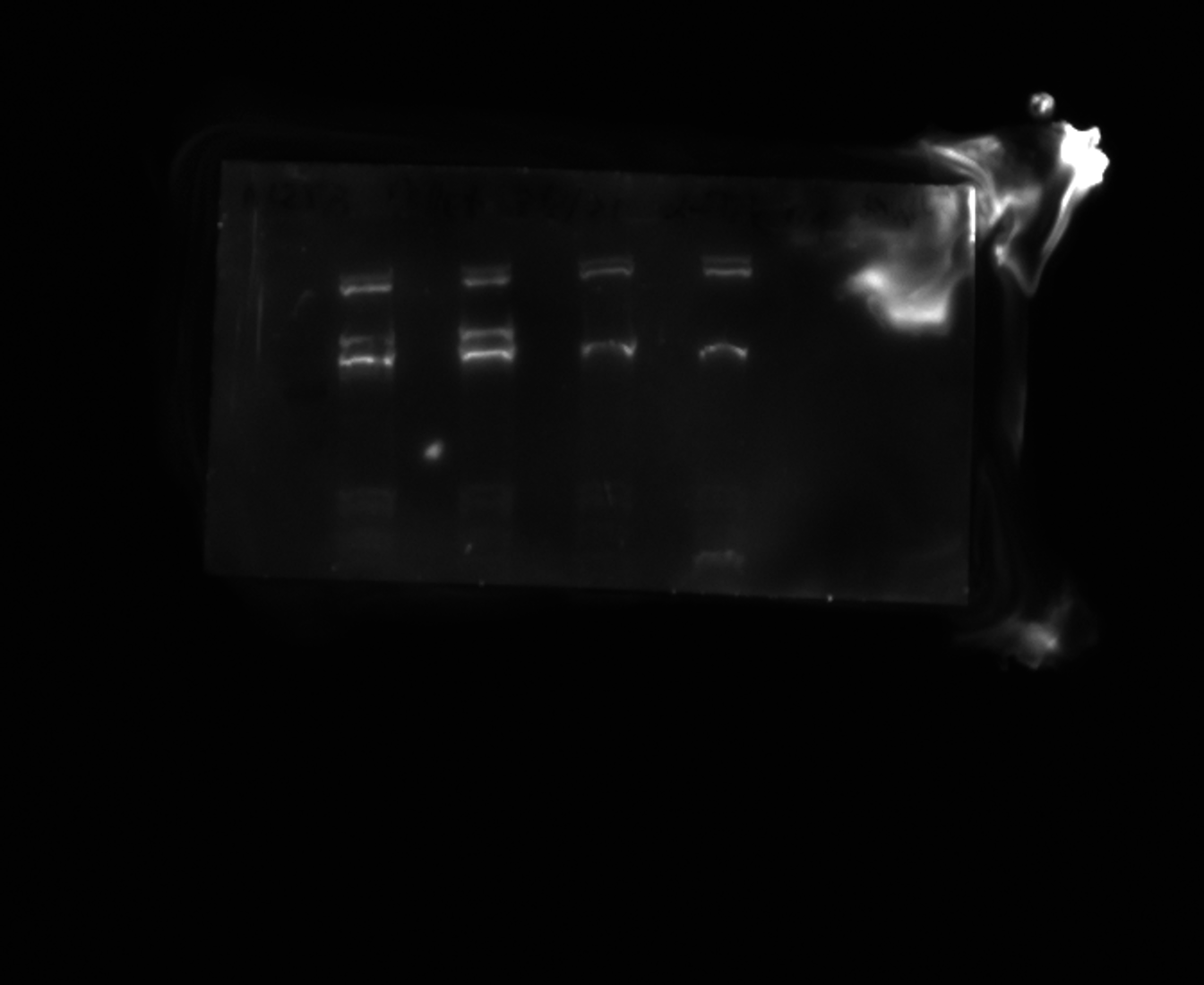

Supplement: Figure 7—source data 4. [file elife-82766-fig7-data4.zip › a-VgrG1/pellet/4-8-21 a-vgrg1 pellet CM 5 sec.Tif]

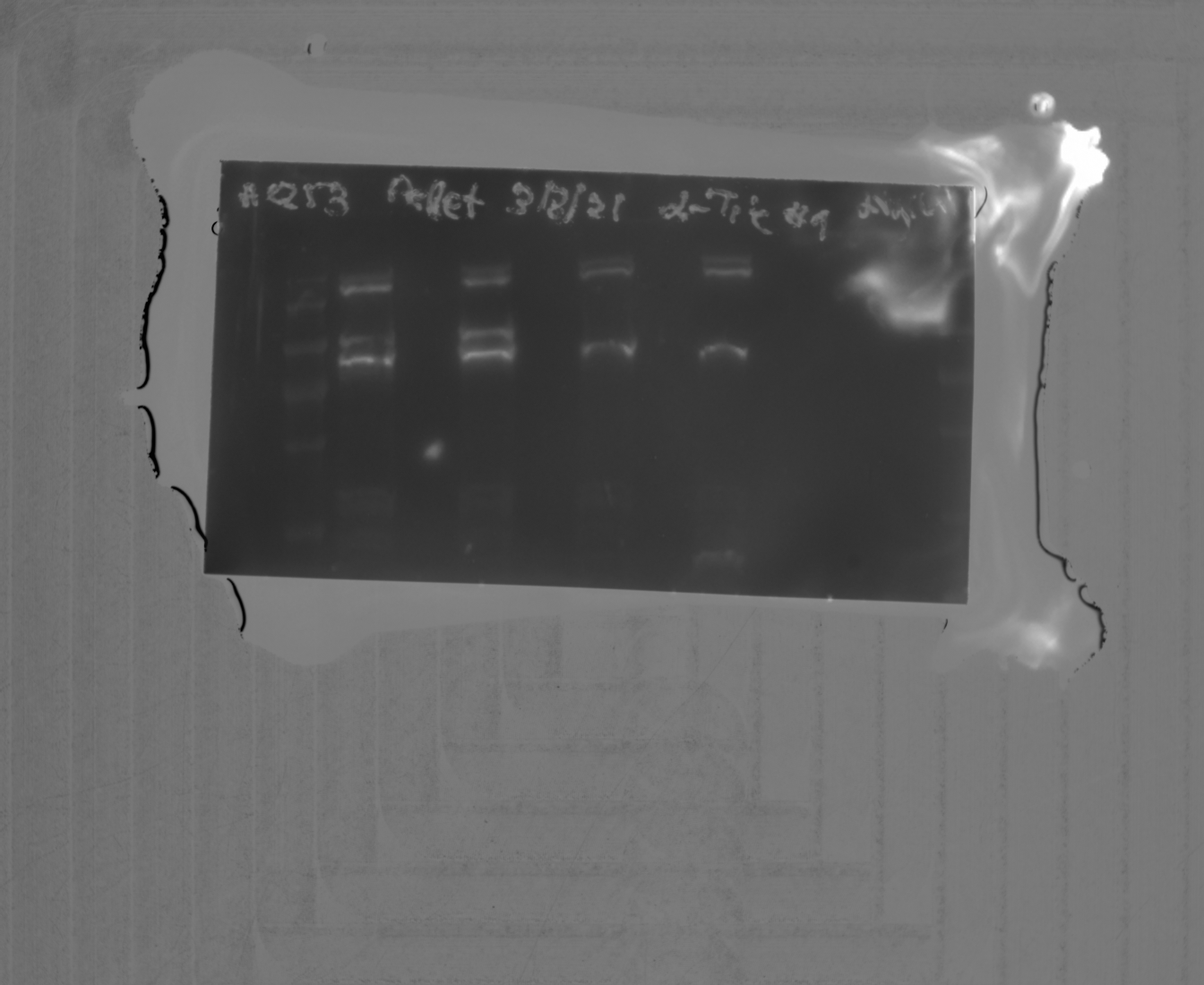

Supplement: Figure 7—source data 4. [file elife-82766-fig7-data4.zip › a-VgrG1/pellet/4-8-21 a-vgrg1 pellet CM 5 secmerge.Tif]

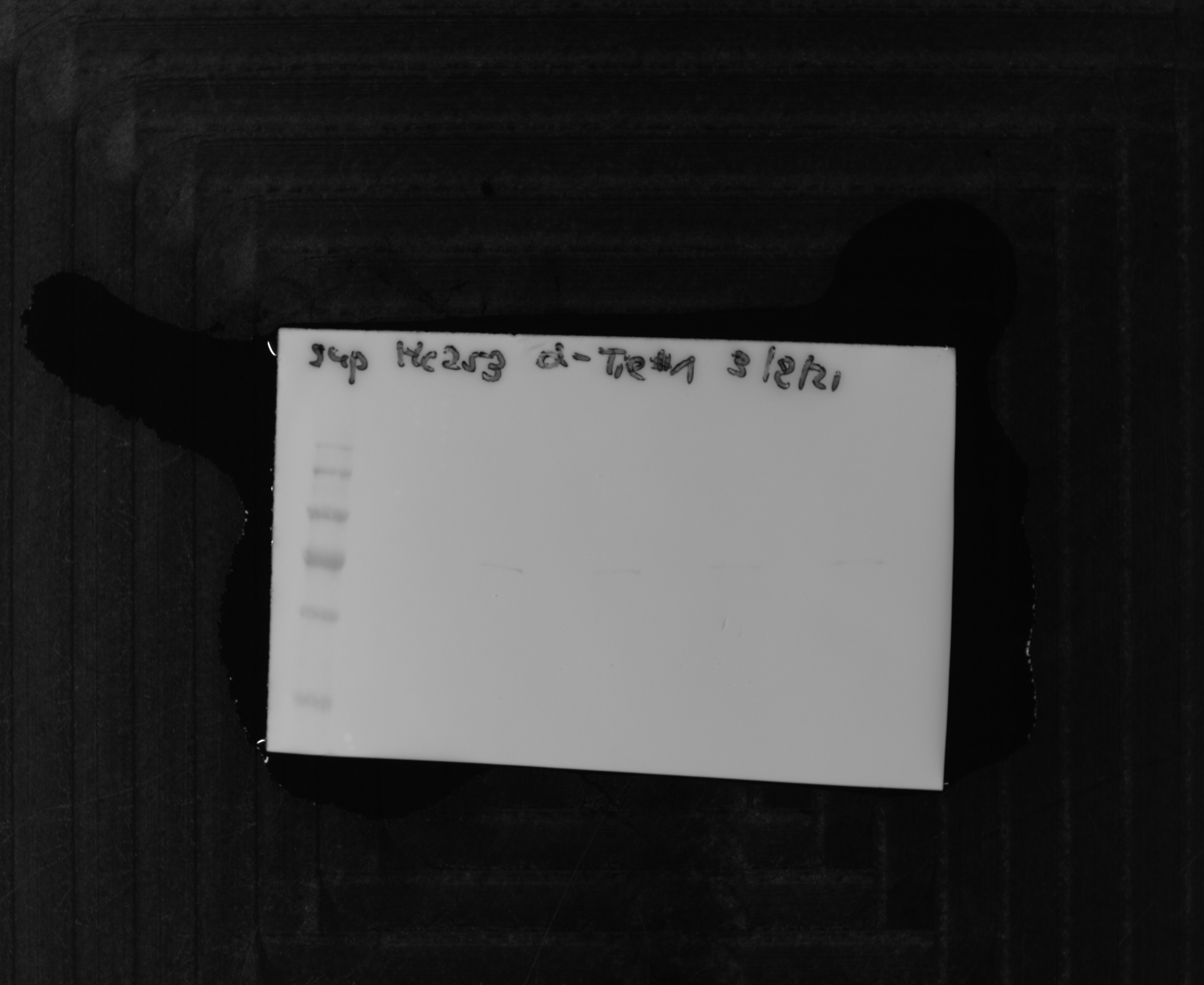

Supplement: Figure 7—source data 4. [file elife-82766-fig7-data4.zip › a-VgrG1/supernatant/4-8-21 a-vgrg1 sup WN 5 sec ladder.Tif]

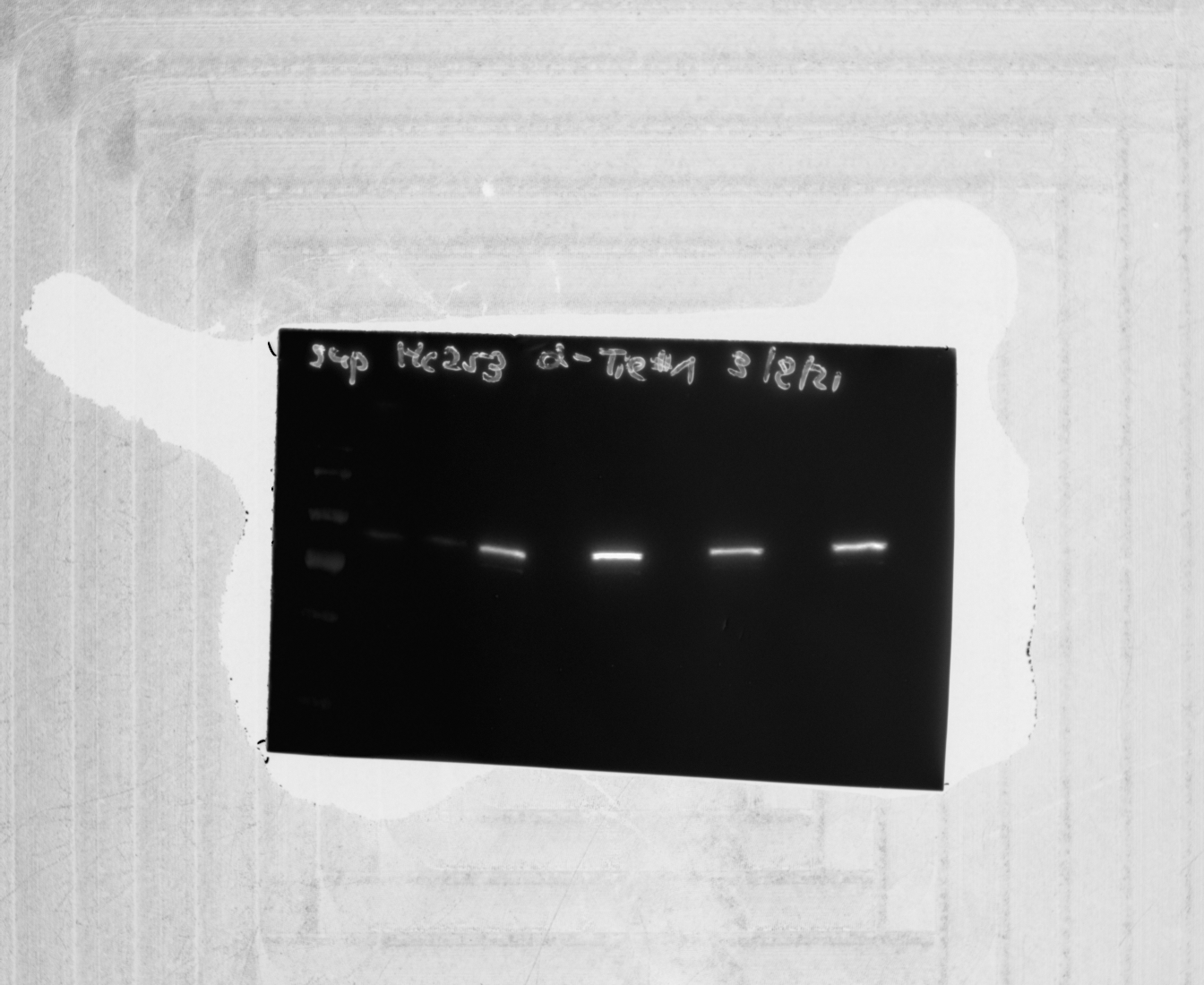

Supplement: Figure 7—source data 4. [file elife-82766-fig7-data4.zip › a-VgrG1/supernatant/4-8-21 a-vgrg1 sup WN 5 sec merge.Tif]

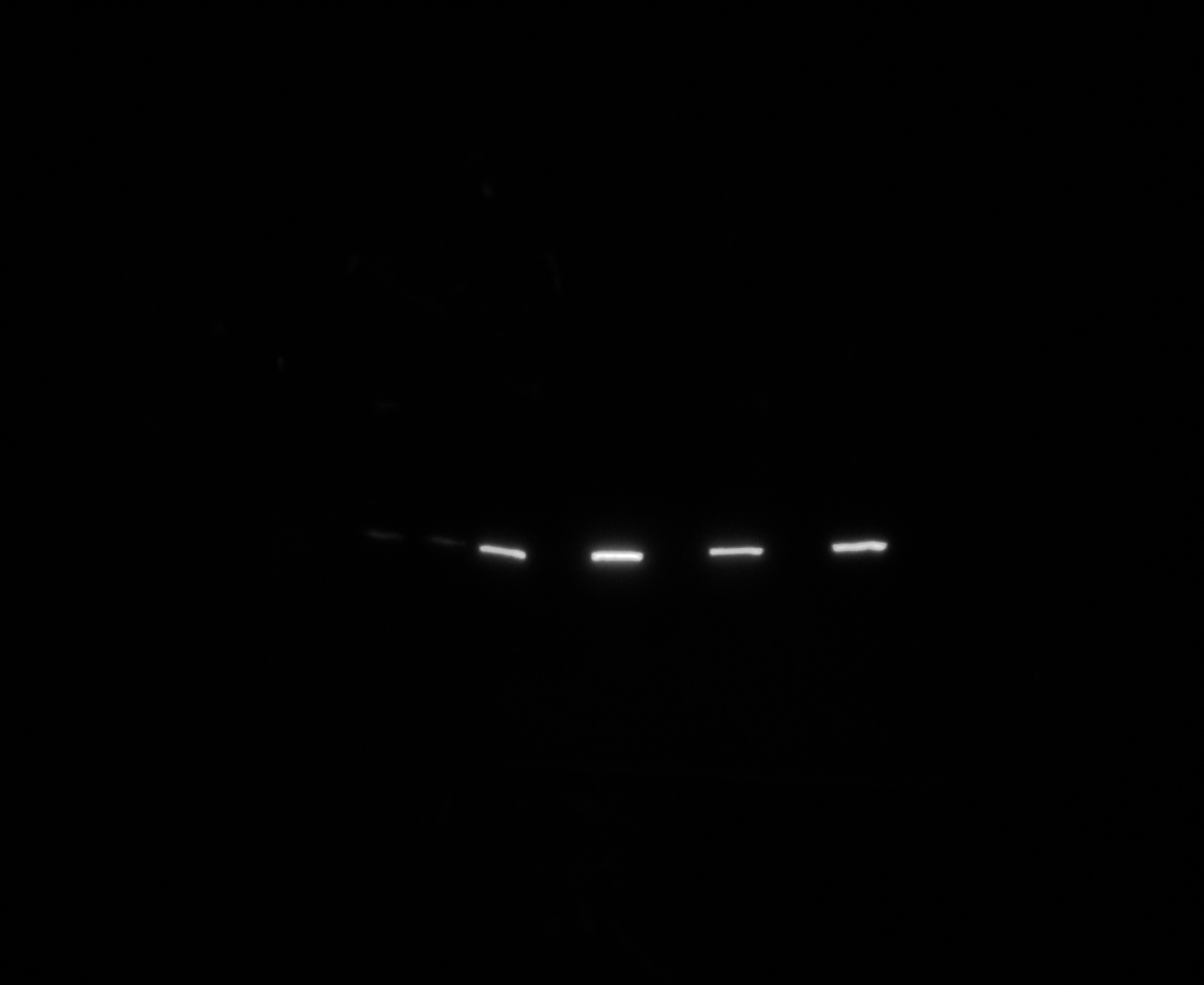

Supplement: Figure 7—source data 4. [file elife-82766-fig7-data4.zip › a-VgrG1/supernatant/4-8-21 a-vgrg1 sup WN 5 sec.Tif]

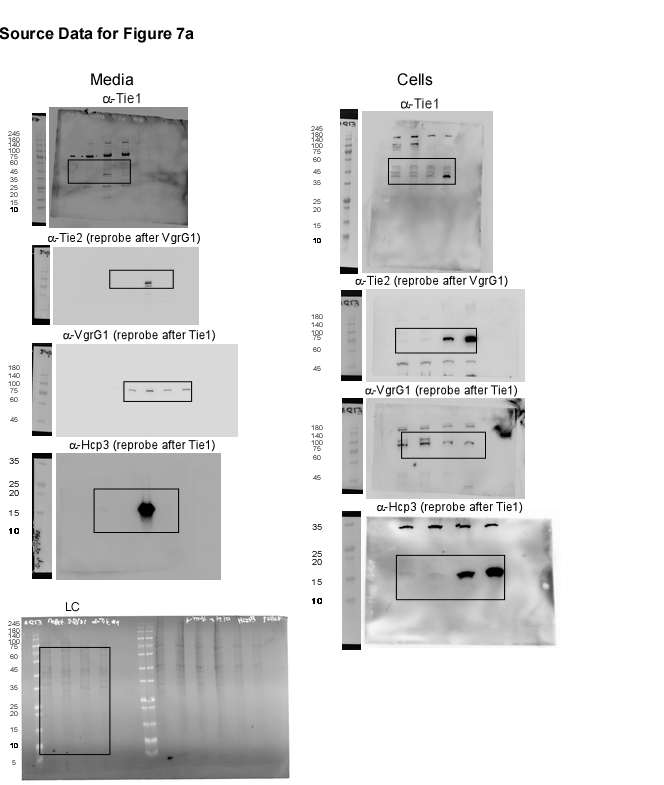

Supplement: Figure 7—source data 5. [file elife-82766-fig7-data5.zip › Figure 7a.tif]

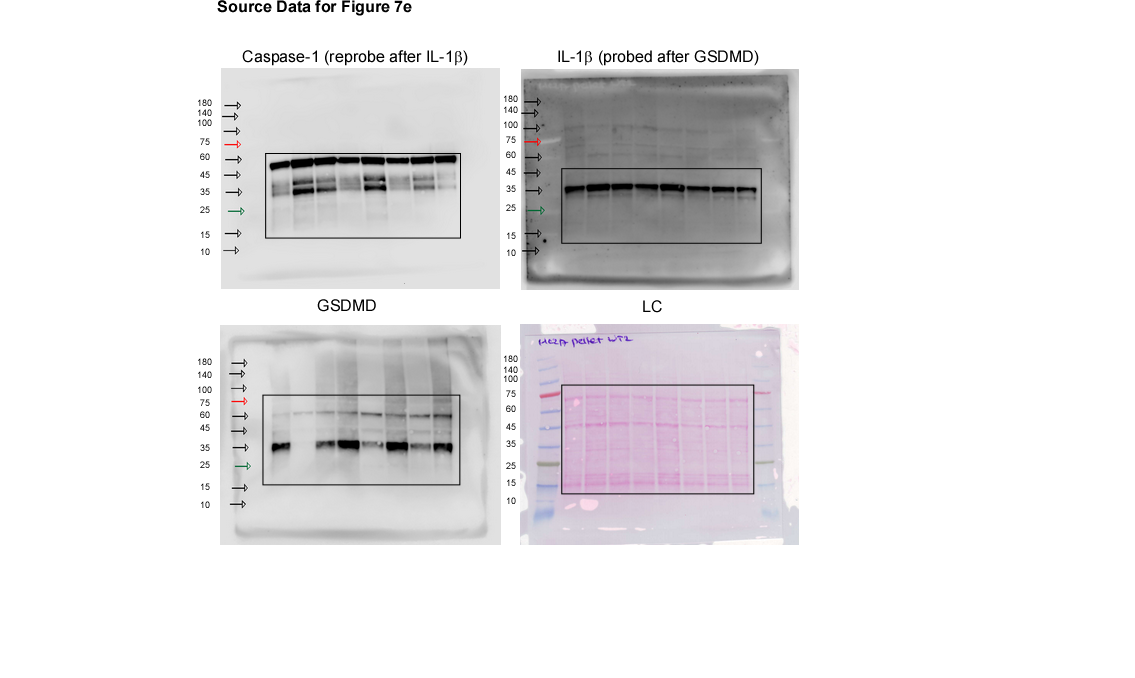

Supplement: Figure 7—source data 6. [file elife-82766-fig7-data6.zip › Figure 7e-source data 1/Figure 7e.tif]

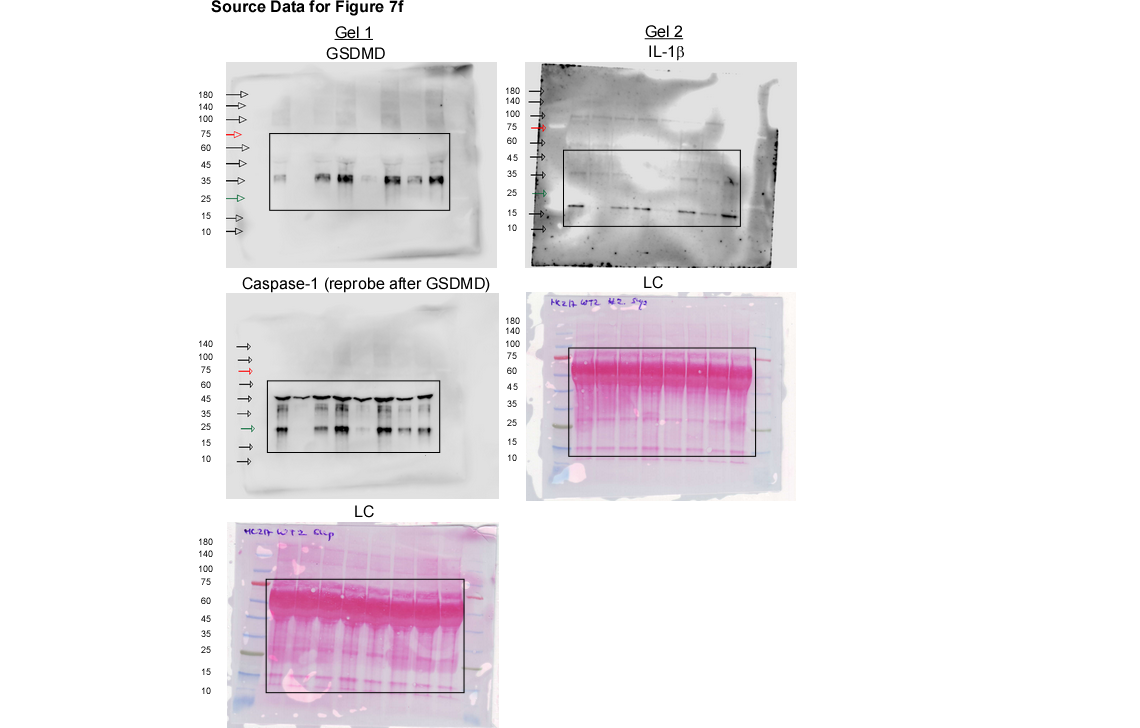

Supplement: Figure 7—source data 7. [file elife-82766-fig7-data7.zip › Figure 7f-source data 1/Figure 7f.tif]

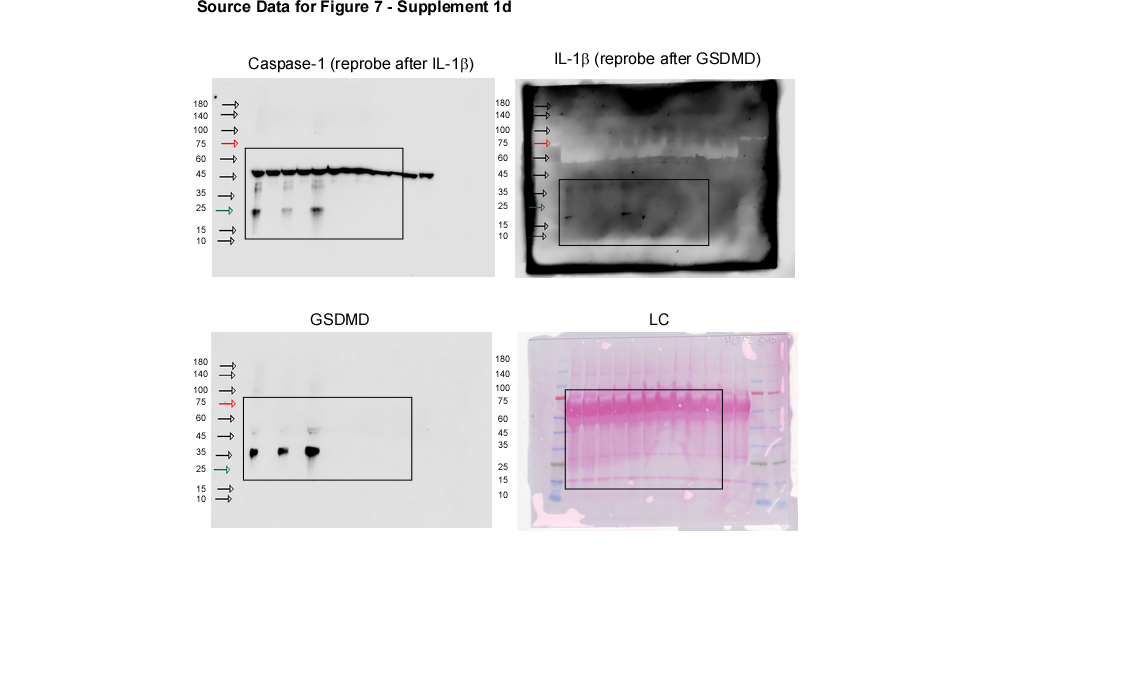

Supplement: Figure 7—figure supplement 1—source data 1. [file elife-82766-fig7-figsupp1-data1.zip › Figure 7-figure supplement 1d-source data 1/figure 7 - supplement 1d.tif]

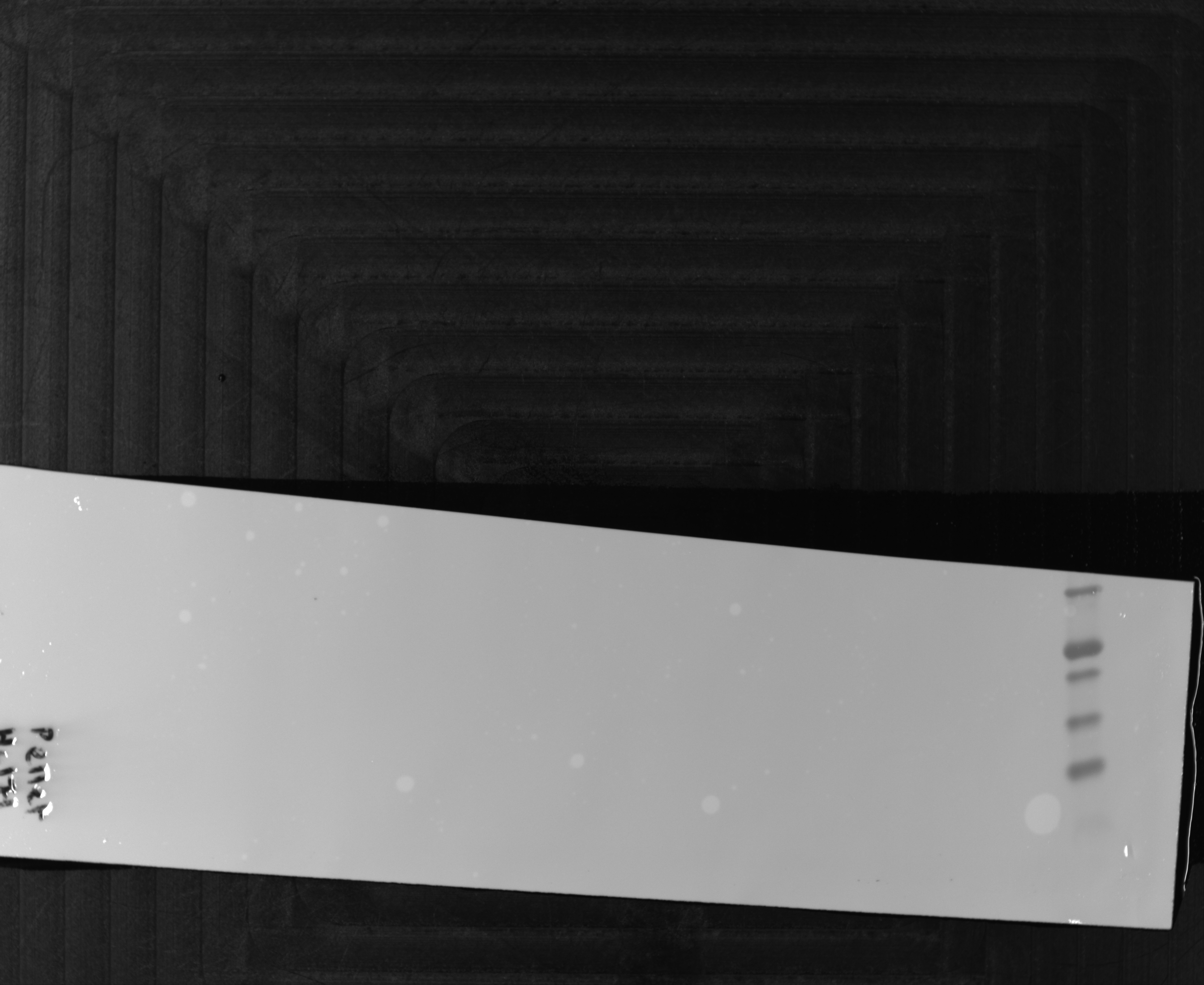

Supplement: Figure 7—figure supplement 1—source data 2. [file elife-82766-fig7-figsupp1-data2.zip › Figure 7-figure supplement 1e-source data 1/a-Hcp3/pellet/23112020 hcp3 CM 3 sec ladder.Tif]

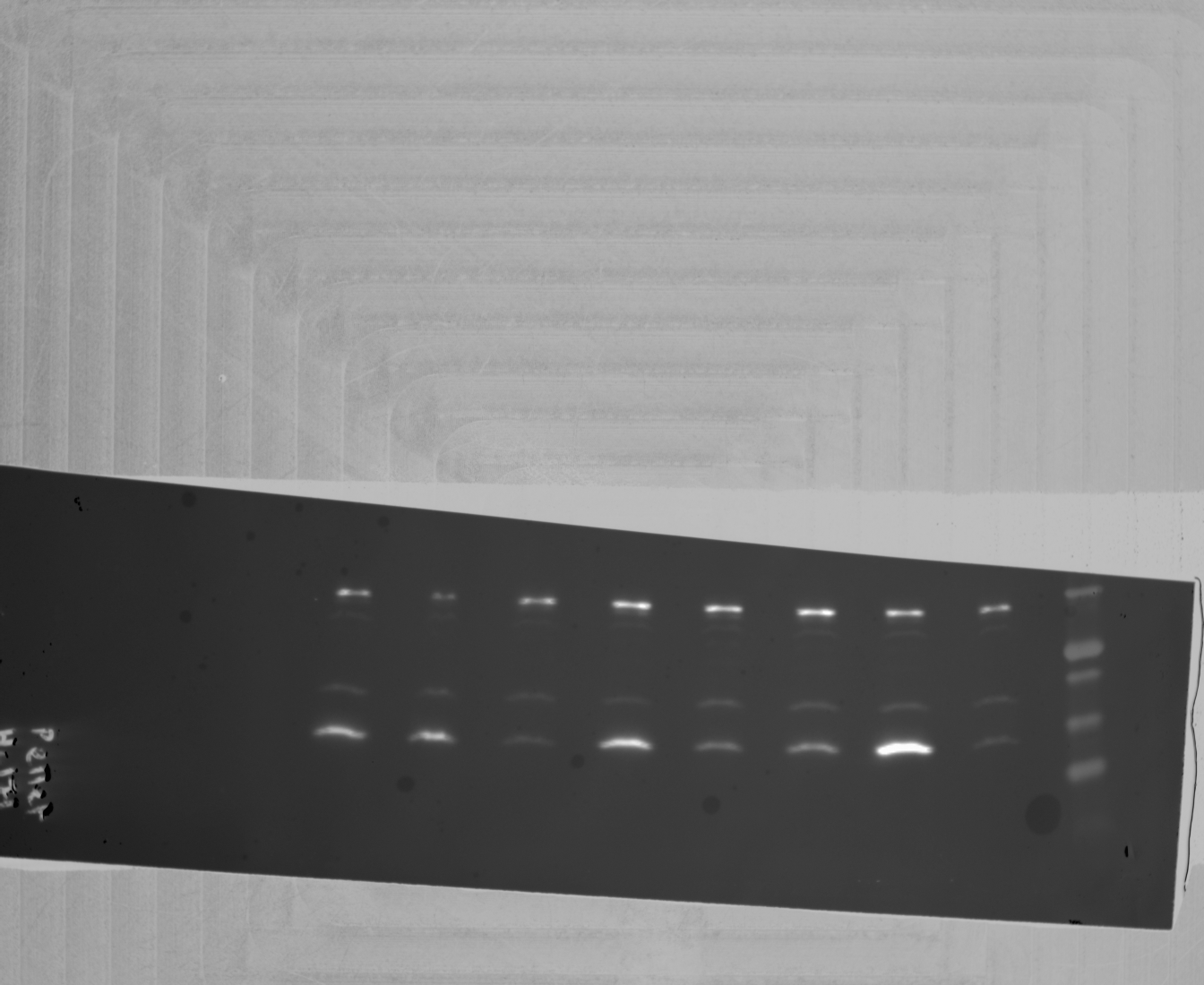

Supplement: Figure 7—figure supplement 1—source data 2. [file elife-82766-fig7-figsupp1-data2.zip › Figure 7-figure supplement 1e-source data 1/a-Hcp3/pellet/23112020 hcp3 CM 3 sec merge.Tif]

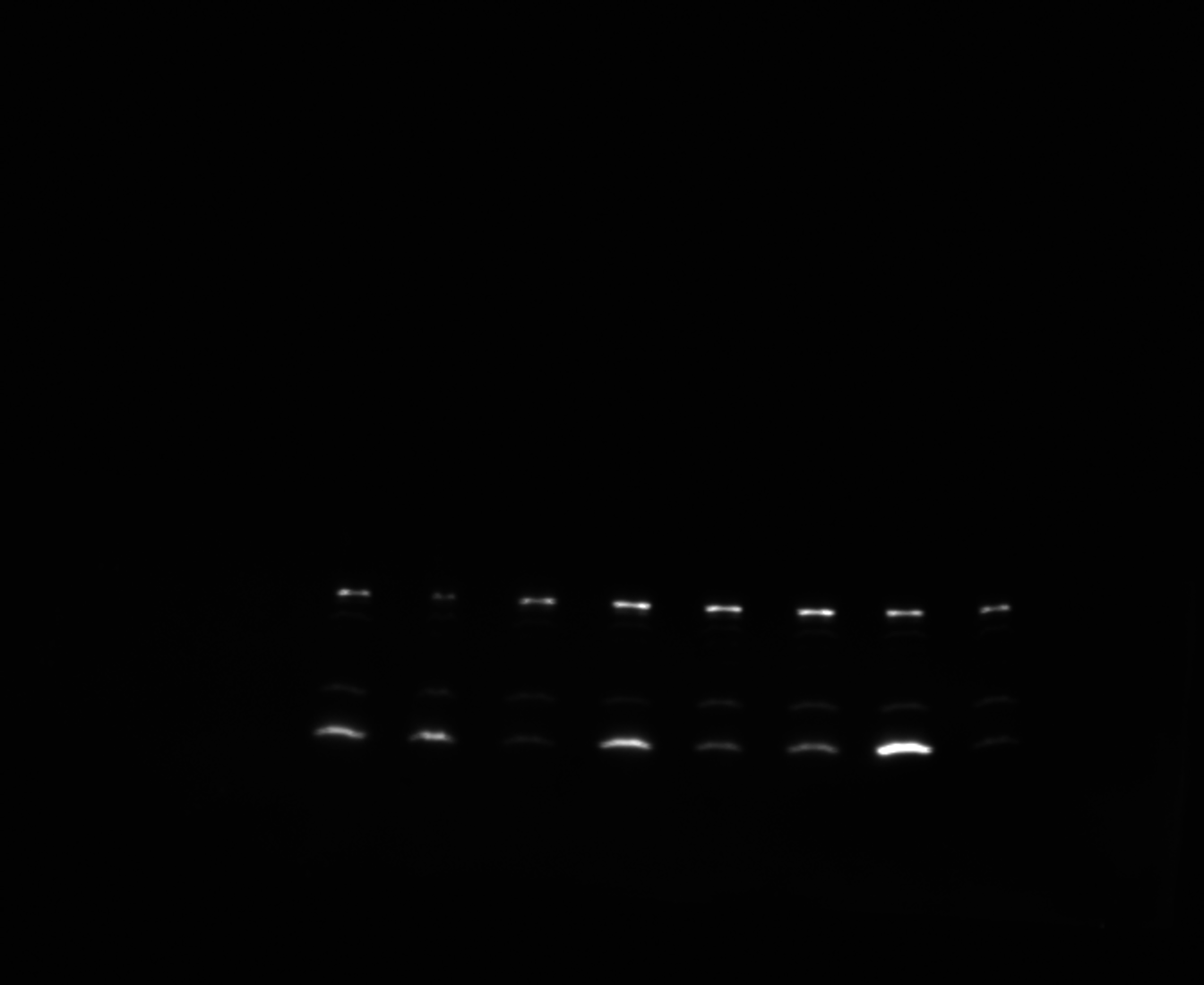

Supplement: Figure 7—figure supplement 1—source data 2. [file elife-82766-fig7-figsupp1-data2.zip › Figure 7-figure supplement 1e-source data 1/a-Hcp3/pellet/23112020 hcp3 CM 3 sec.Tif]

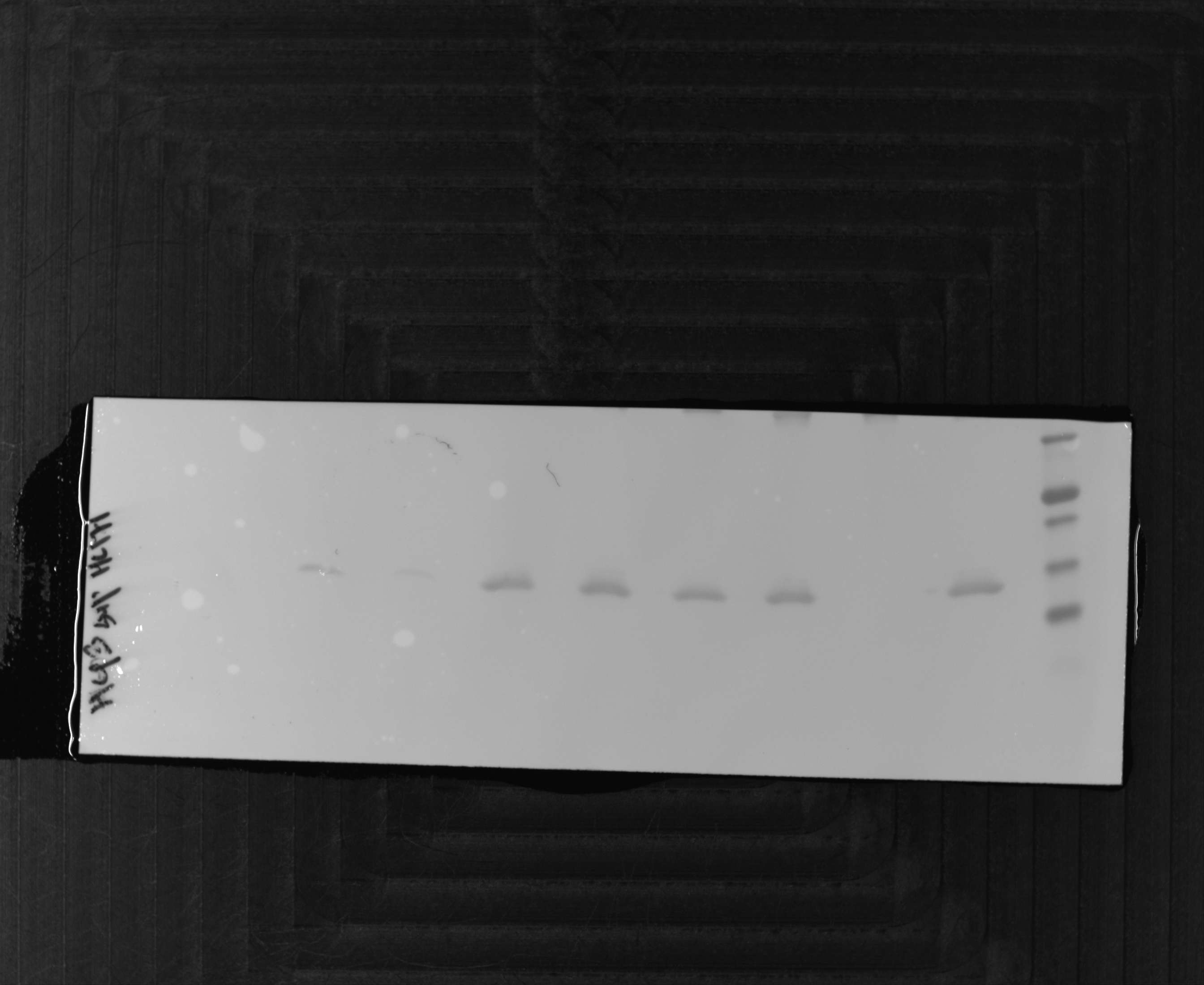

Supplement: Figure 7—figure supplement 1—source data 2. [file elife-82766-fig7-figsupp1-data2.zip › Figure 7-figure supplement 1e-source data 1/a-Hcp3/supernatant/23112020 hcp3 wn 3 sec sup ladder.Tif]

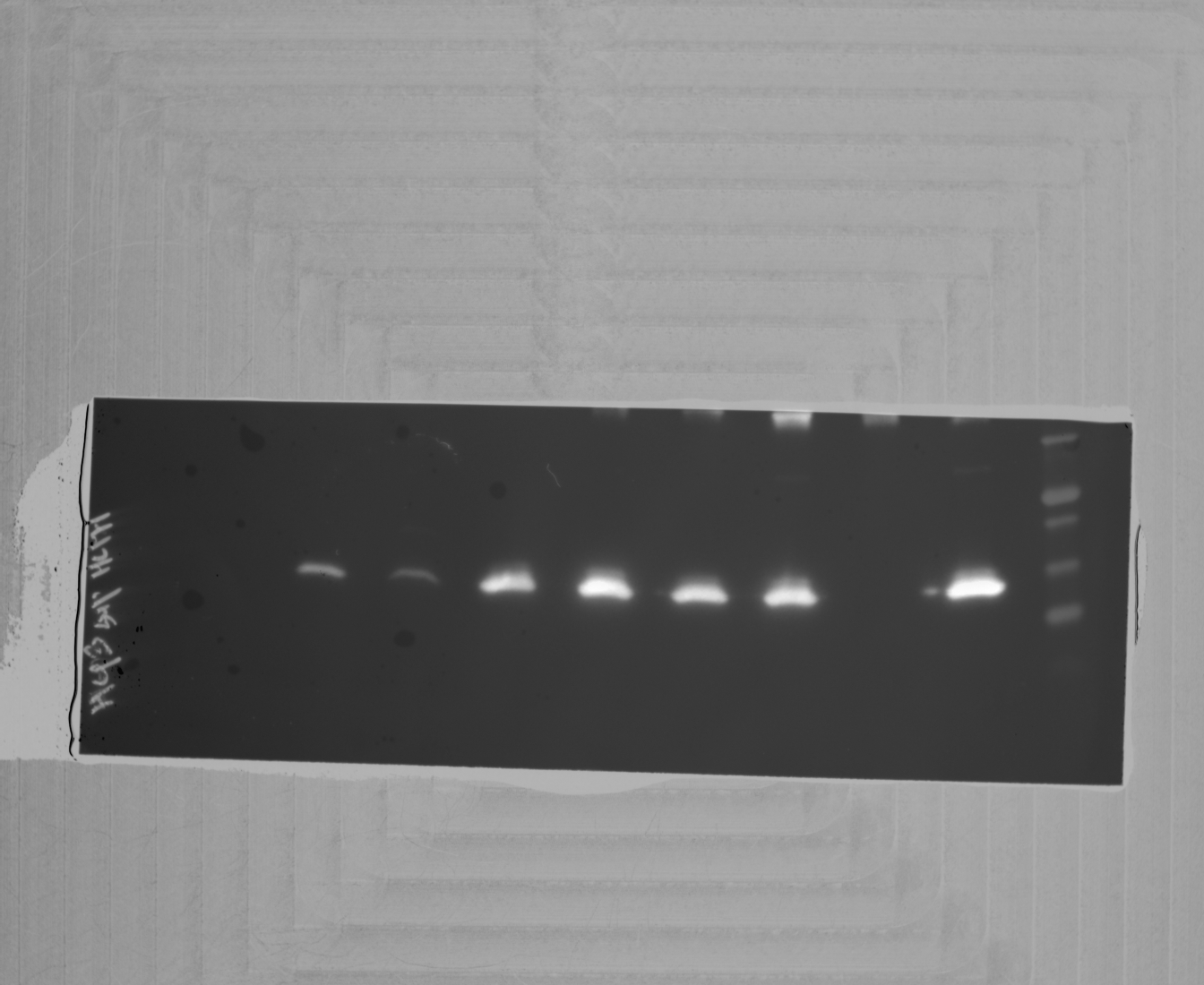

Supplement: Figure 7—figure supplement 1—source data 2. [file elife-82766-fig7-figsupp1-data2.zip › Figure 7-figure supplement 1e-source data 1/a-Hcp3/supernatant/23112020 hcp3 wn 3 sec sup merge.Tif]

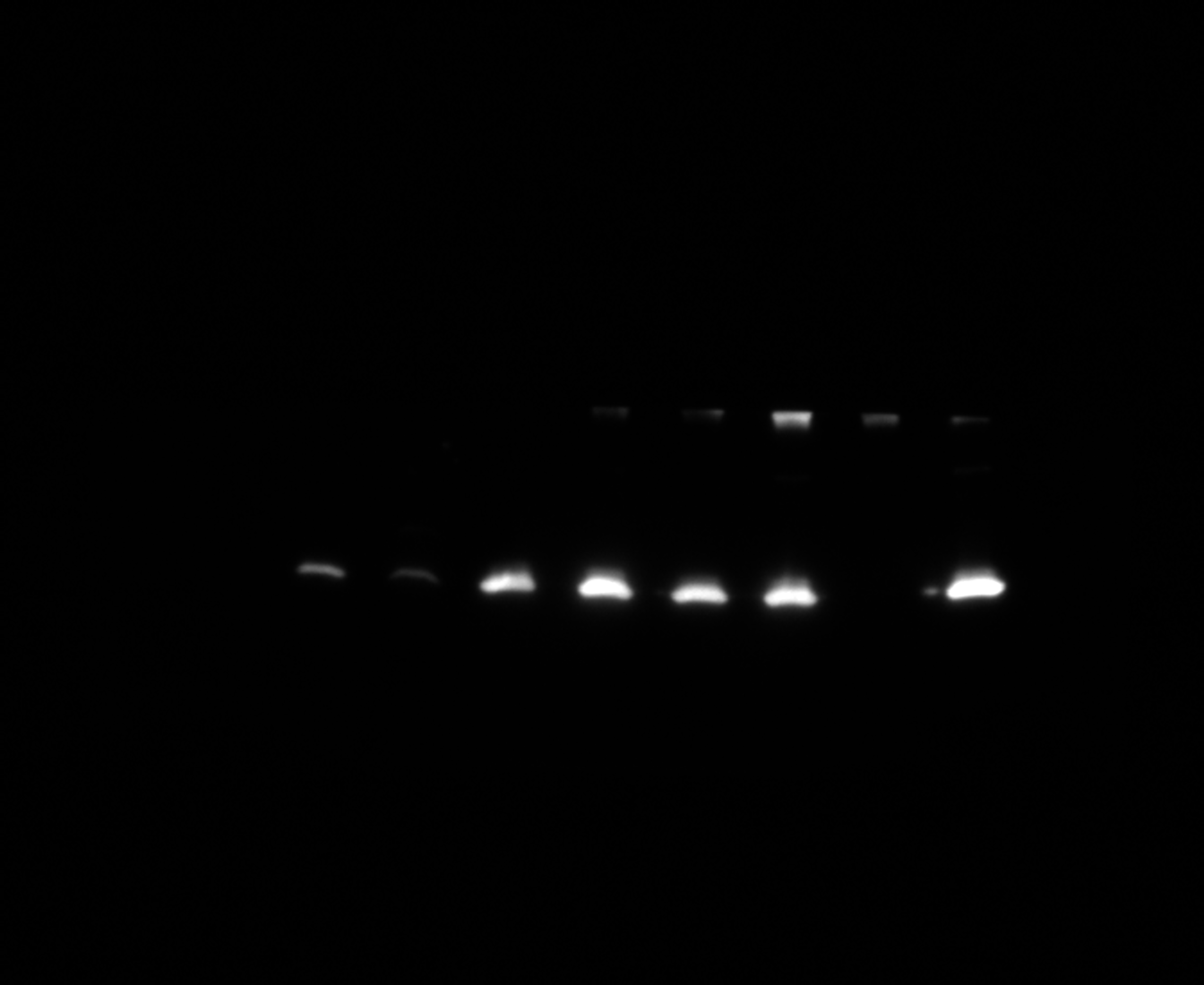

Supplement: Figure 7—figure supplement 1—source data 2. [file elife-82766-fig7-figsupp1-data2.zip › Figure 7-figure supplement 1e-source data 1/a-Hcp3/supernatant/23112020 hcp3 wn 3 sec sup.Tif]

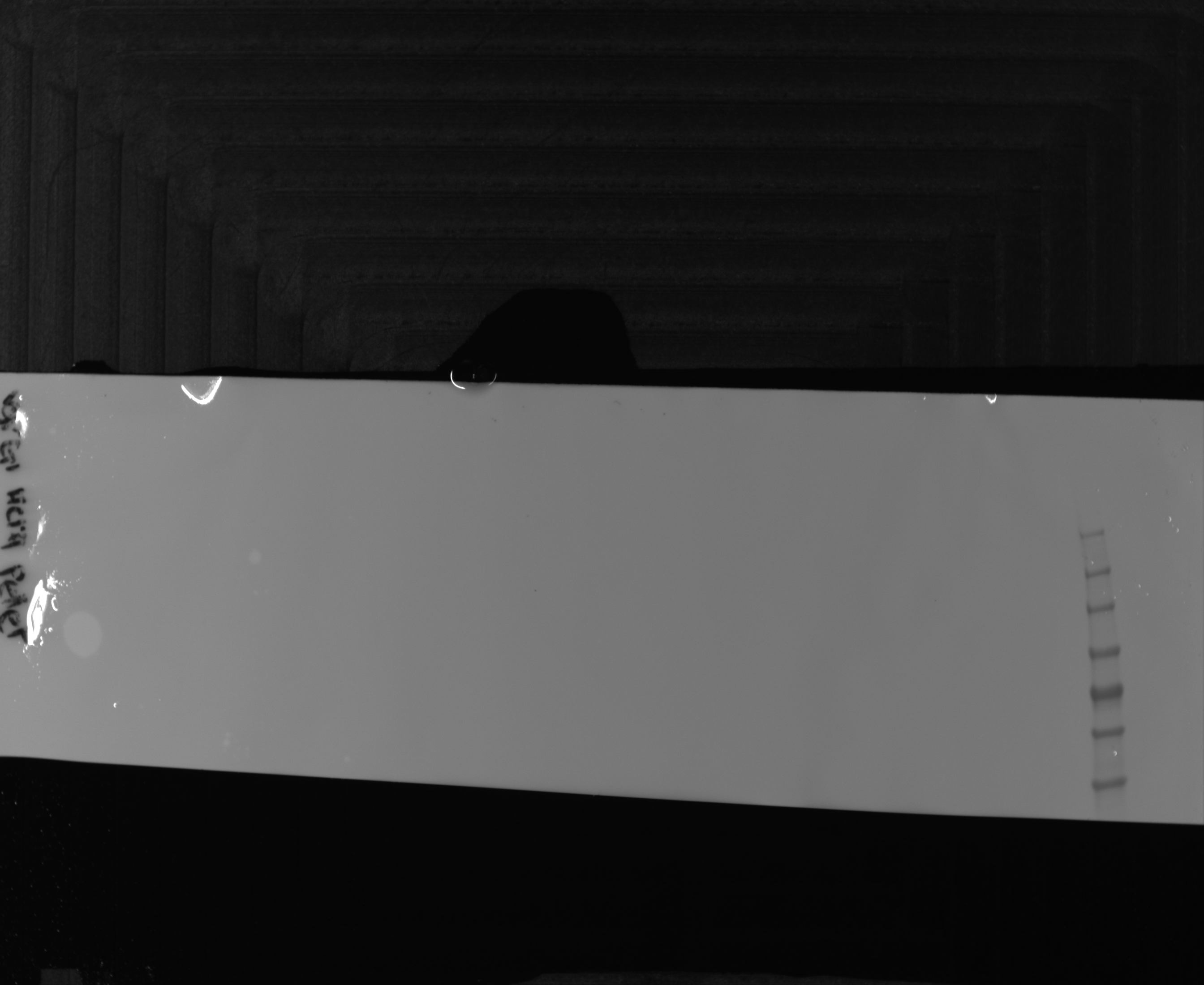

Supplement: Figure 7—figure supplement 1—source data 2. [file elife-82766-fig7-figsupp1-data2.zip › Figure 7-figure supplement 1e-source data 1/a-VgrG1/pellet/23112020 vgrg1 wn 1 20 min pellet ladder.Tif]

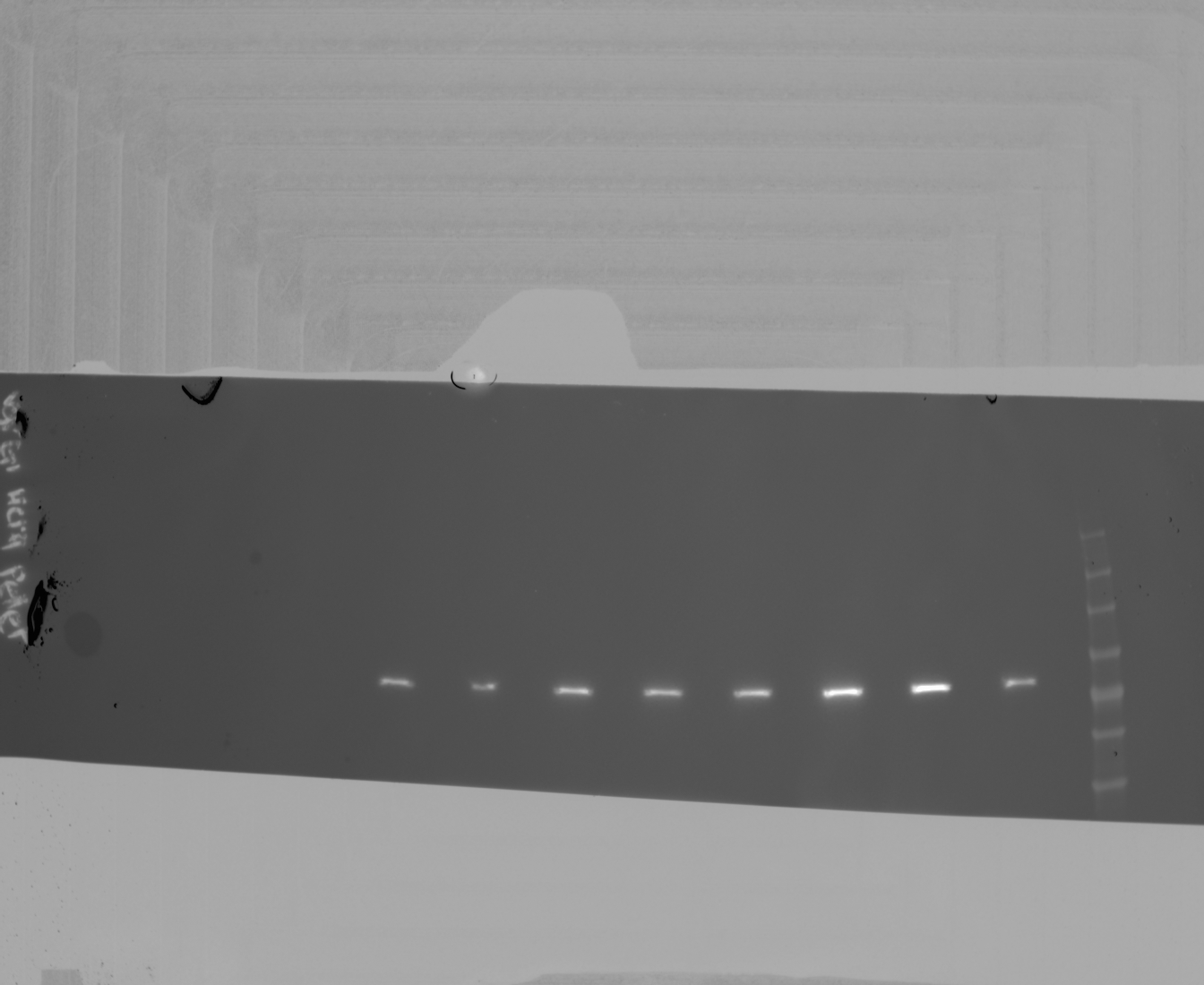

Supplement: Figure 7—figure supplement 1—source data 2. [file elife-82766-fig7-figsupp1-data2.zip › Figure 7-figure supplement 1e-source data 1/a-VgrG1/pellet/23112020 vgrg1 wn 1 20 min pellet merge.Tif]

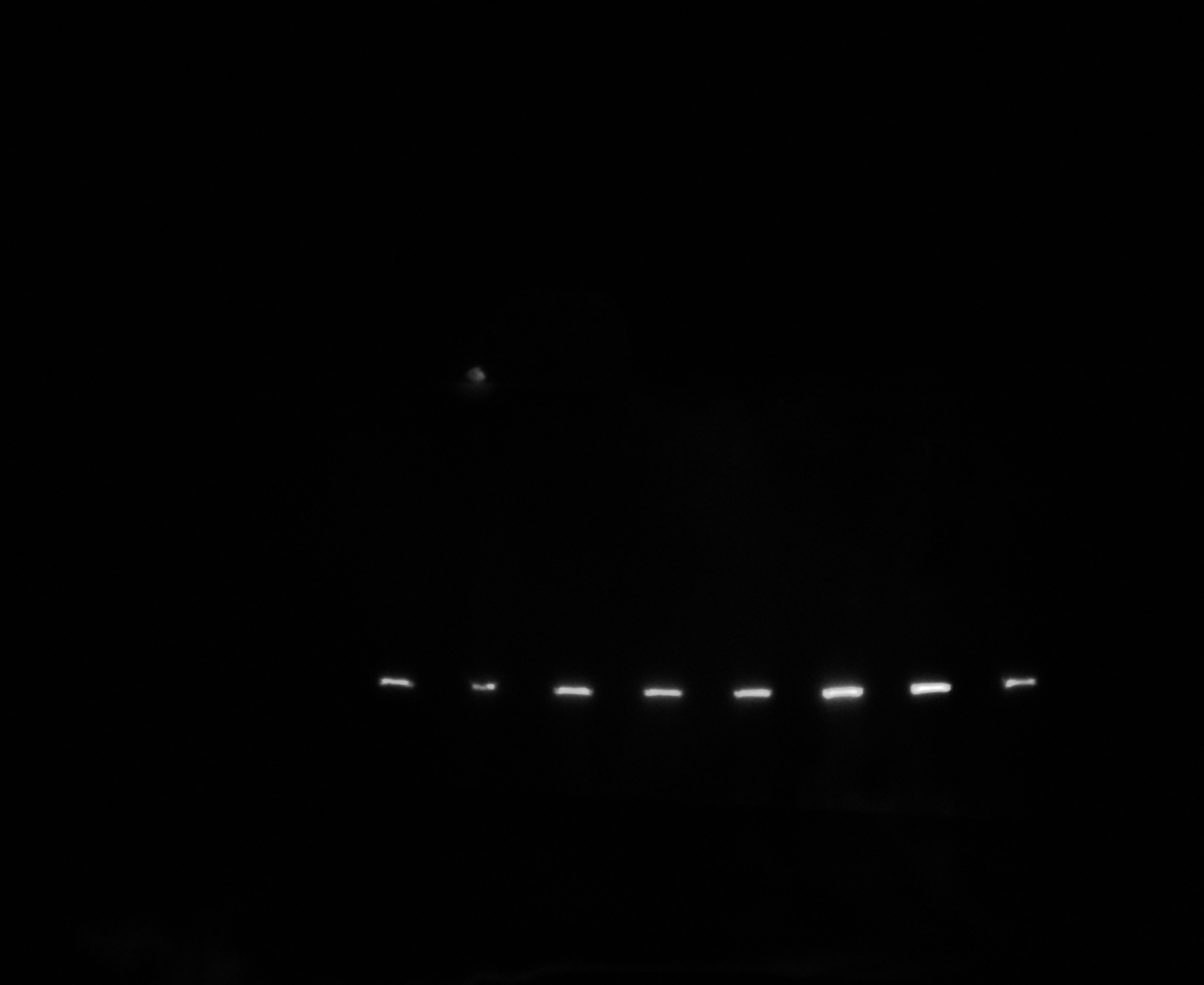

Supplement: Figure 7—figure supplement 1—source data 2. [file elife-82766-fig7-figsupp1-data2.zip › Figure 7-figure supplement 1e-source data 1/a-VgrG1/pellet/23112020 vgrg1 wn 1 20 min.Tif]

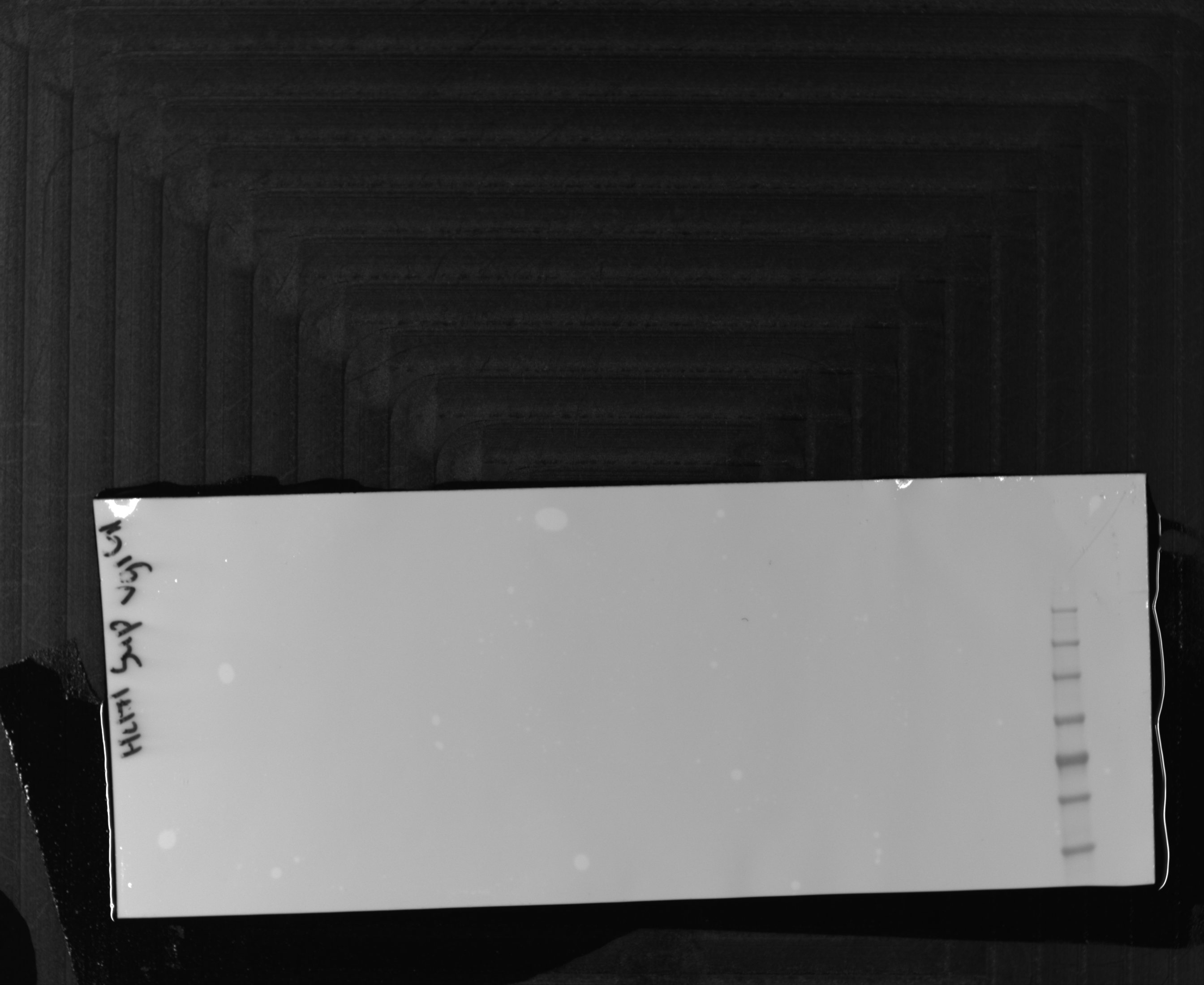

Supplement: Figure 7—figure supplement 1—source data 2. [file elife-82766-fig7-figsupp1-data2.zip › Figure 7-figure supplement 1e-source data 1/a-VgrG1/supernatant/23112020 vgrg1 wn 17 sec sup ladder.Tif]

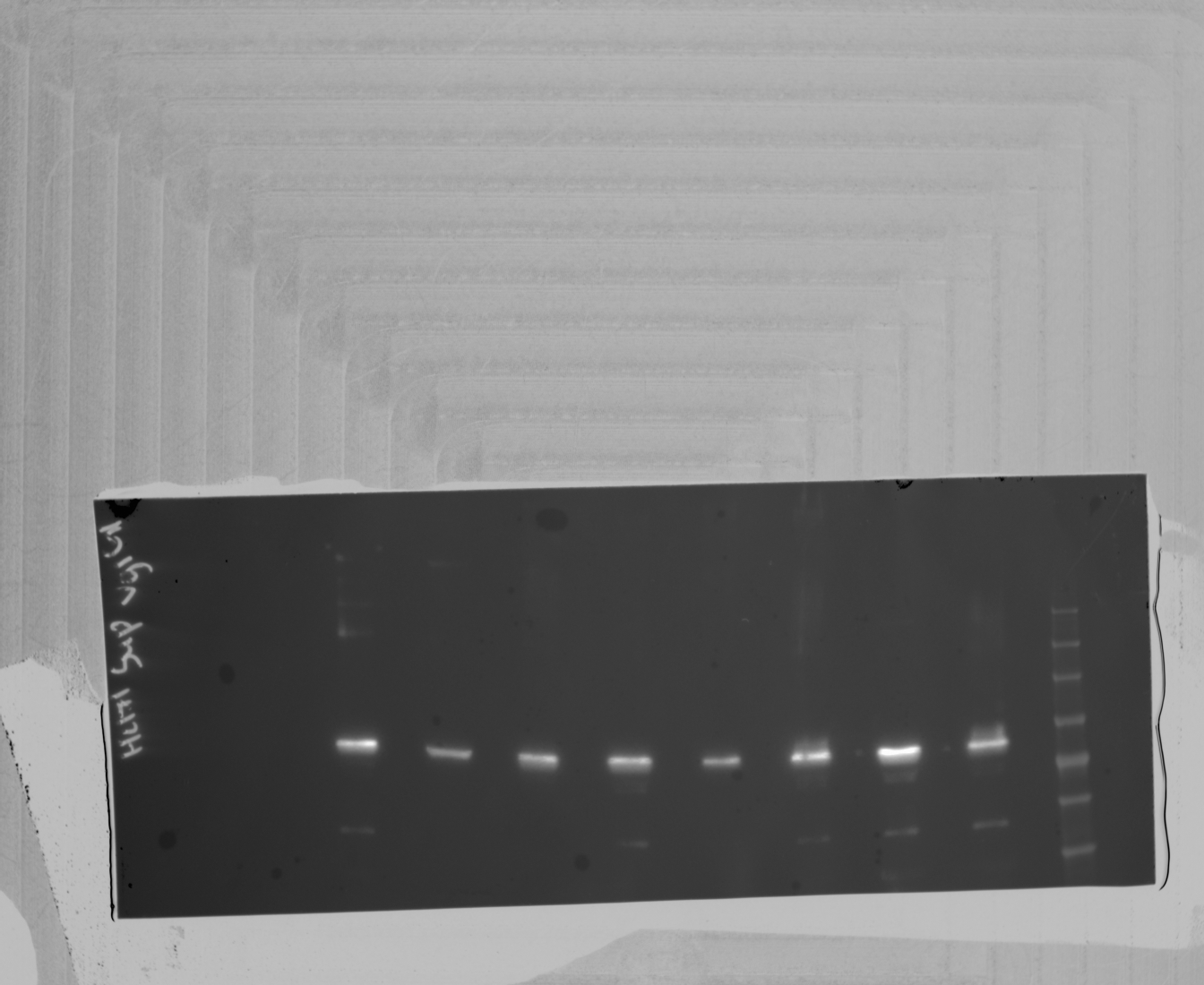

Supplement: Figure 7—figure supplement 1—source data 2. [file elife-82766-fig7-figsupp1-data2.zip › Figure 7-figure supplement 1e-source data 1/a-VgrG1/supernatant/23112020 vgrg1 wn 17 sec sup merge.Tif]

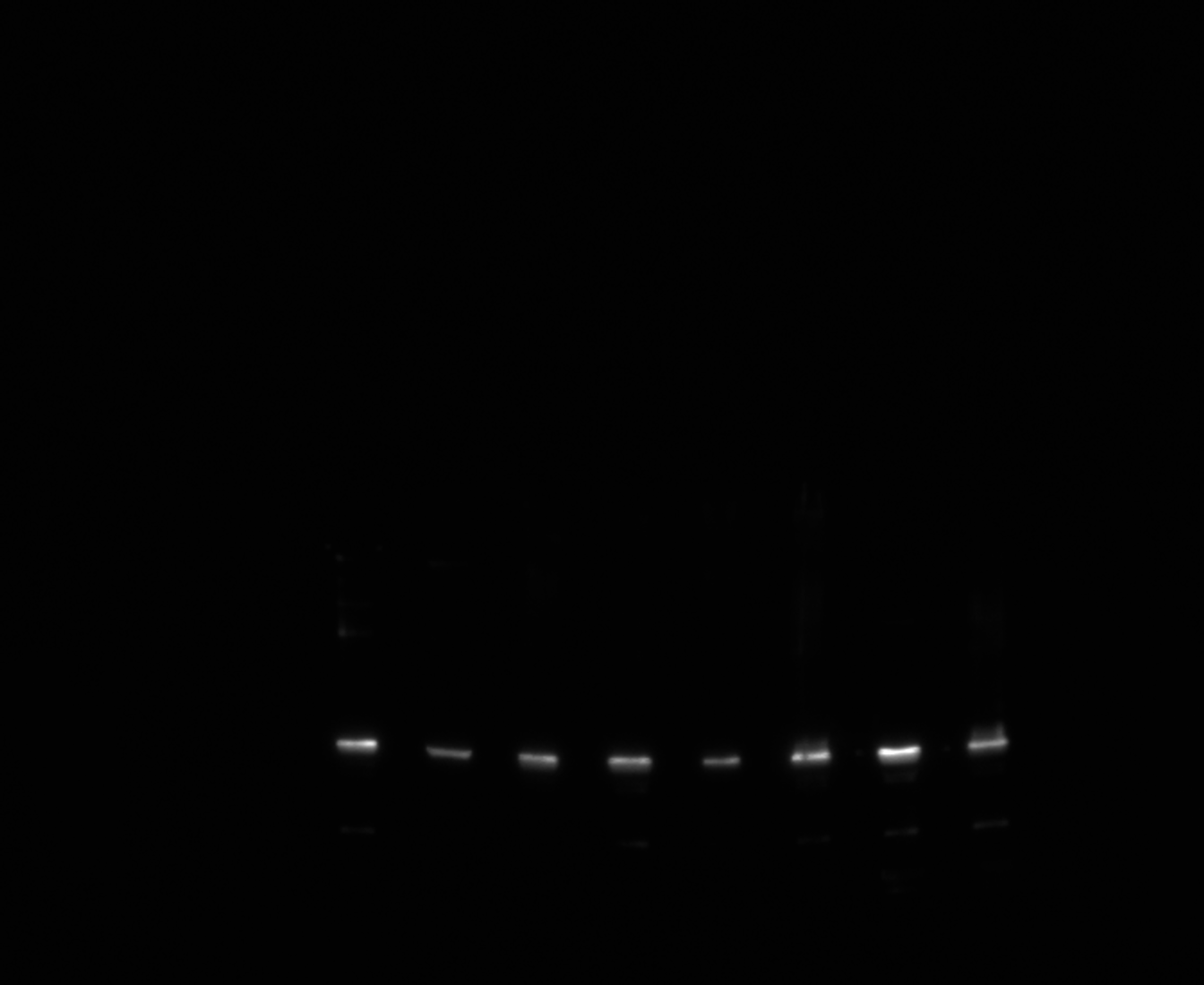

Supplement: Figure 7—figure supplement 1—source data 2. [file elife-82766-fig7-figsupp1-data2.zip › Figure 7-figure supplement 1e-source data 1/a-VgrG1/supernatant/23112020 vgrg1 wn 17 sec sup.Tif]

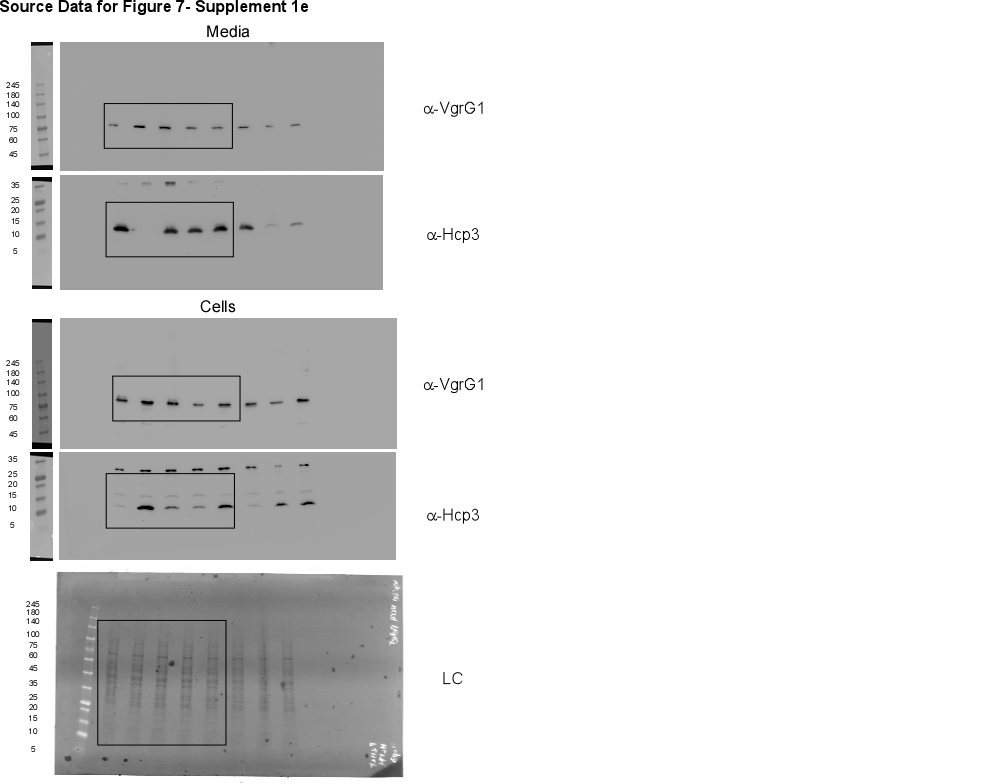

Supplement: Figure 7—figure supplement 1—source data 2. [file elife-82766-fig7-figsupp1-data2.zip › Figure 7-figure supplement 1e-source data 1/Figure 7- Supplement 1e.tif]
