# Supplementary material for: Post-phagocytosis activation of NLRP3 inflammasome by two novel T6SS effectors
Source: eLife. 2022 Sep 26;11:e82766. doi: 10.7554/eLife.82766 (PMC9545535; doi:10.7554/eLife.82766)
Supplement: Supplementary file 1. [file elife-82766-supp1.docx]

**Supplemental Table S1.** **A list of bacterial strains used in this study.**

| **Strain name** | **Genotype** | **Source** |
| --- | --- | --- |
| *Vibrio proteolyticus* ATCC 15338 | Wild-type | ATCC |
| T6SS1^-^ | *Vibrio proteolyticus* ATCC 15338 Δ*tssG1* | (Ray et al., 2017) |
| T6SS3^-^ | *Vibrio proteolyticus* ATCC 15338 Δ*tssL3* | This study |
| Δ*vprh* | *Vibrio proteolyticus* ATCC 15338 Δ*vprh* | (Ray et al., 2016) |
| Δ*vprh*/T6SS1^-^ | *Vibrio proteolyticus* ATCC 15338 Δ*vprh*/Δ*tssG1* | This study |
| Δ*vprh*/T6SS3^-^ | *Vibrio proteolyticus* ATCC 15338 Δ*vprh*/Δ*tssL3* | This study |
| Δ*vprh*/Δ*hns1* | *Vibrio proteolyticus* ATCC 15338 Δ*vprh*/ Δ*hns1* | This study |
| Δ*vprh*/Δ*hns1*/T6SS1^-^ | *Vibrio proteolyticus* ATCC 15338 Δ*vprh*/Δ*hns1*/Δ*tssG1* | This study |
| Δ*vprh*/Δ*hns1*/T6SS3^-^ | *Vibrio proteolyticus* ATCC 15338 Δ*vprh*/Δ*hns1*/Δ*tssL3* | This study |
| Δ*vprh*/Δ*hns1*/Δ*tie1* | *Vibrio proteolyticus* ATCC 15338 Δ*vprh*/Δ*hns1*/Δ*tie1* | This study |
| Δ*vprh*/Δ*hns1*/Δ*tie2* | *Vibrio proteolyticus* ATCC 15338 Δ*vprh*/Δ*hns1*/Δ*tie2* | This study |
| Δ*vprh*/Δ*hns1*/Δ*tie1*/Δ*tie2* | *Vibrio proteolyticus* ATCC 15338 Δ*vprh*/Δ*hns1*/*Δtie1*/Δ*tie2* | This study |
| *V. para* | *V. parahaemolyticus* RIMD 2210633 *ΔtdhAS* derivative (strain POR1) | (Park et al., 2004) |
| *Escherichia coli* XL-1 blue | XL-1 Blue | Purchased from Addgene |
