# Supplementary material for: Post-phagocytosis activation of NLRP3 inflammasome by two novel T6SS effectors
Source: eLife. 2022 Sep 26;11:e82766. doi: 10.7554/eLife.82766 (PMC9545535; doi:10.7554/eLife.82766)
Supplement: Supplementary file 2. [file elife-82766-supp2.docx]

**Supplemental Table S2. A list of plasmids used in this study.**

| **Plasmid name** | **Description** | **Comments** | **Source** |
| --- | --- | --- | --- |
| pDM4 | a CmR and oriV^R6K^-containing suicide vector | Used as a backbone to construct plasmids for gene deletions in *Vibrio* | (O’Toole et al, 1996) |
| pDM4:*vprh* | pDM4 containing 1 kb upstream and 1 kb downstream of *vprh* in its MCS | Used to delete *vprh* in *V. proteolyticus* | (Ray et al., 2016) |
| pDM4:*hns1* | pDM4 containing 1 kb upstream and 1 kb downstream of *hns1* in its MCS | Used to delete *hns1* in *V. proteolyticus* | This study |
| pDM4:*tssG1* | pDM4 containing 1 kb upstream and 1 kb downstream of *tssG1* in its MCS | Used to delete *tssG1* in *V. proteolyticus* | (Ray et al., 2017) |
| pDM4:*tssL3* | pDM4 containing 1 kb upstream and 1 kb downstream of *tssL3* in its MCS | Used to delete *tssL3* in *V. proteolyticus* | This study |
| pDM4:*tie1* | pDM4 containing 1 kb upstream and 1 kb downstream of the region corresponding to nucleotides 485-584 of *tie1* in its MCS | Used to delete a 100 bp region and inactivate *tie1* in *V. proteolyticus* | This study |
| pDM4:*tie2* | pDM4 containing 1 kb upstream and 1 kb downstream of *tie2* in its MCS | Used to delete *tie2* in *V. proteolyticus* | This study |
| pBAD/Myc-His^Kan^ | pBR322 ori-containing plasmid harboring a Kan^R^ cassette, araC, and an MCS following a P*bad* promoter | Used as a backbone to construct plasmids for arabinose-inducible gene expression | (Salomon et al., 2013) |
| pTssL3 | pBAD/Myc-His^Kan^ containing the *tssL3* ORF in its MCS, in frame with a C-terminal Myc-His tag | Used for arabinose-inducible expression of TssL3 | This study |
| pAts3 | pBAD/Myc-His^Kan^ containing the *ats3* ORF in its MCS, not fused to a C-terminal tag | Used for arabinose-inducible expression of Ats3 | This study |
| pTie1 | pBAD/Myc-His^Kan^ containing the *tie1* ORF in its MCS, not fused to a C-terminal tag | Used for arabinose-inducible expression of Tie1 | This study |
| pTie2 | pBAD/Myc-His^Kan^ containing the *tie2* ORF in its MCS, not fused to a C-terminal tag | Used for arabinose-inducible expression of Tie2 | This study |
| pTie1-2 | pBAD/Myc-His^Kan^ containing the ORFs of *tie1* and *tie2* in its MCS, not fused to a C-terminal tag | Used for arabinose-inducible expression of Tie1 and Tie2, together | This study |
| pBAD33.1 | p15A ori-containing plasmid carrying a CmR gene, araC, and an MCS following a Pbad promoter. | Used for selection in competition assay | Purchased from Addgene; (Chung and Raetz, 2010) |
