# Supplementary material for: Post-phagocytosis activation of NLRP3 inflammasome by two novel T6SS effectors
Source: eLife. 2022 Sep 26;11:e82766. doi: 10.7554/eLife.82766 (PMC9545535; doi:10.7554/eLife.82766)
Supplement: Supplementary file 3. [file elife-82766-supp3.docx]

**Supplemental Table S3. A list of primers used in this study.**

| **Primer name** | **Sequence (5’-3’)** | **Description** |
| --- | --- | --- |
| VprHNS1_UP_F_SacI | CAGCGAGCTCAAGACACTGGACACGGTAG | Used to amplify 1 kb upstream of *hns1* to construct pDM4:*hns1* |
| VprHNS1_UP_R_BamH | CAACGGATCCGACCATTCCTATGAATTTAATAAAGTC |  |
| VprHNS1_DN_F_BamHI | CACCGGATCCTCGTAAGATTGGTTTAAAAAAGG | Used to amplify 1 kb downstream of *hns1* to construct pDM4:*hns1* |
| VprHNS1_DN_R_SalI | CAACGTCGACCTATCGTTACCTGTGCAAC |  |
| VprTssL3_UP_F_SpeI | CACCACTAGTTGAAGACAGCCGTTTGCG | Used to amplify 1 kb upstream of *tssL3* to construct pDM4:*tssL3* |
| VprTssL3_UP_R_HindIII | CAACAAGCTTAACTACCTACCTGATCAC |  |
| VprTssl3_DN_F_HindIII | CAGGAAGCTTTATGATCAAAAAAATTCTTG | Used to amplify 1 kb downstream of *tssL3* to construct pDM4:*tssL3* |
| VprTssL3_DN_R_SphI | CAACGCATGCCCGGTTTTCACGCCCGATG |  |
| VPR_ Tie1 _SacI_UP_F | CAAAGAGCTCGCGCAGACTTACGTTAAG | Used to amplify 1 kb upstream of nucleotide 455 in *tie1* to construct pDM4:*tie1* |
| VPR_ Tie1 _XbaI_Up_R | CACCTCTAGATTTTGTAGAATCGTTCGCCCTGG |  |
| VPR_ Tie1 _XbaI_DN_F | CACCTCTAGATGAGCTGGGCATCAAGGCTGG | Used to amplify 1 kb downstream of nucleotide 584 in *tie1* to construct pDM4:*tie1* |
| VPR_Tie1 _SalI_DN_R | CAAAGTCGACTTTGCAAATTTTGCAGCAAACG |  |
| VPR_Tie2_UP_F_SacI | CATTGAGCTCACGGCCGGTGAATTTAACG | Used to amplify 1 kb upstream of *tie2* to construct pDM4:*tie2* |
| VPR_Tie2_UP_R_HindIII | CAACAAGCTTAGGCCTTTCCTTTTTATTAACGTG |  |
| VPR_Tie2_DN_F_HindIII | CACGAAGCTTTCCTTGCCAACATAGCGG | Used to amplify 1 kb downstream of *tie2* to construct pDM4:*tie2* |
| VPR_Tie2_DN_R_SalI | CAGCGTCGACAATCTATAACACTCACCG |  |
| tssL3_F_pBADfix | GCTAACAGGAGGAATTAACCATGGCAGGACTTTTTAACG | Used to amplify *tssL3* to construct pTssL3 |
| tssL3_R_pBADfix | TTTTGTTCGGGCCCAAGCTTTTTCTGCGCTCTTCTTATCG |  |
| Tie1_F_pBADfix | GCTAACAGGAGGAATTAACCATGATAAATGATTTACAAAATGCC | Used to amplify *tie1* to construct pTie1and pTie1-2 |
| Tie1_R_pBADfix | TTTTGTTCGGGCCCAAGCTTGAATGTGCTCAGAATGTCCTGC | Used to amplify *tie1* to construct pTie1 |
| Tie2_F_pBADfix | TTTTGTTCGGGCCCAAGCTTTCACGCGGCTTCCGGGTGTG | Used to amplify *tie2* to construct pTie2 |
| Tie1_Tie2_R_pBADfix | TTTTGTTCGGGCCCAAGCTTCGCGGCTTCCGGGTGTGGAGGCAGTAC | Used to amplify *tie2* to construct pTie2 and pTie1-2 |
| Ats3_F_pBADfix | GCTAACAGGAGGAATTAACCATGGAAAGAAAATCTATAACACTC | Used to amplify *Ats3* to construct pAts3 |
| Ats3_R_pBADfix | TTTTGTTCGGGCCCAAGCTTTTAGCGCTGATAGCGTTTG |  |
